# iSNO-PseAAC: Predict Cysteine S-Nitrosylation Sites in Proteins by Incorporating Position Specific Amino Acid Propensity into Pseudo Amino Acid Composition

**DOI:** 10.1371/journal.pone.0055844

**Published:** 2013-02-07

**Authors:** Yan Xu, Jun Ding, Ling-Yun Wu, Kuo-Chen Chou

**Affiliations:** 1 Department of Information and Computer Science, University of Science and Technology Beijing, Beijing, China; 2 Institute of Applied Mathematics, Academy of Mathematics and Systems Science, Chinese Academy of Sciences, Beijing, China; 3 Gordon Life Science Institute, San Diego, California, United States of America; Russian Academy of Sciences, Institute for Biological Instrumentation, Russian Federation

## Abstract

Posttranslational modifications (PTMs) of proteins are responsible for sensing and transducing signals to regulate various cellular functions and signaling events. S-nitrosylation (SNO) is one of the most important and universal PTMs. With the avalanche of protein sequences generated in the post-genomic age, it is highly desired to develop computational methods for timely identifying the exact SNO sites in proteins because this kind of information is very useful for both basic research and drug development. Here, a new predictor, called iSNO-PseAAC, was developed for identifying the SNO sites in proteins by incorporating the position-specific amino acid propensity (PSAAP) into the general form of pseudo amino acid composition (PseAAC). The predictor was implemented using the conditional random field (CRF) algorithm. As a demonstration, a benchmark dataset was constructed that contains 731 SNO sites and 810 non-SNO sites. To reduce the homology bias, none of these sites were derived from the proteins that had 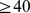 pairwise sequence identity to any other. It was observed that the overall cross-validation success rate achieved by iSNO-PseAAC in identifying nitrosylated proteins on an independent dataset was over 90%, indicating that the new predictor is quite promising. Furthermore, a user-friendly web-server for iSNO-PseAAC was established at http://app.aporc.org/iSNO-PseAAC/, by which users can easily obtain the desired results without the need to follow the mathematical equations involved during the process of developing the prediction method. It is anticipated that iSNO-PseAAC may become a useful high throughput tool for identifying the SNO sites, or at the very least play a complementary role to the existing methods in this area.

## Introduction

**Figure 1:**
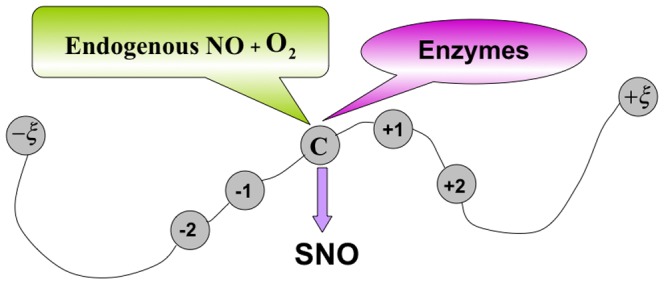
A schematic illustration to show the S-nitrosylation (SNO) site of a protein segment. The protein segment contains 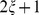 residues, where C (cysteine) is located at the center of the peptide and all the other amino acids are depicted as an open circle with a number to indicate their sequential positions, respectively.

The post-translational modifications (PTMs) play a key role in providing proteins with structural and functional diversity, as well as in regulating cellular plasticity and dynamics. As illustrated in **Fig. 1**, the PTMs are covalent processing events that change the properties of a protein by proteolytic cleavage for adding a modifying group to one or more amino acids [1]. One of the most important and universal PTMs is S-nitrosylation (SNO). Recent reports have indicated that SNO can modulate protein stability and activities [2], [3], as well as play an important role in a variety of biological processes, including cell signaling, transcriptional regulation, apoptosis, and chromatin remodeling [4].

Meanwhile, increasing evidences have indicated that SNO also plays an important role in various major diseases [5], such as cancer [6], Parkinson's [7], [8], Alzheimer's [9], and Amyotrophic Lateral Sclerosis (ALS) [10].

Therefore, identifying the SNO sites in proteins is very important to both basic science and drug development.

Many experimental methods have been developed for identifying SNO sites, such as BST (biotin switch assay) [11], SNOSID [2], [12], and SNO-RAC [13]. These methods have indeed provided very useful information in this area. Unfortunately, as pointed out by Seth and Stamler [14], experimental identification of SNO sites with a site-directed mutagenesis strategy is laborious and low-throughput due to the labile nature and the low-abundance of SNO. Particularly, with the avalanche of protein sequences generated in the postgenomic age, it is highly desired to develop computational method for timely and reliably identifying the SNO sites in proteins.

Actually, some computational methods have been proposed in this regard. For instance, based on a benchmark dataset consisting of 65 positive and 65 negative samples, Gross and co-workers [15] developed a computational method called SNOSID for identifying the SNO sites in proteins. A few years later, based on 549 experimentally verified SNO sites in 363 proteins, Xue et al [16] proposed a different method called GRS-SNO for the same purpose. Shortly afterwards, Li et al. [17] tried to improve the prediction performance by introducing the SVM (support vector machine) algorithm. Recently, Li et al. [18] proposed a predictor by means of the nearest neighbor algorithm (NNA) with the maximum relevance minimum redundancy (mRMR) approach. Each of the aforementioned methods has its own merit and did play a role in stimulating the development of this area although bearing various limits. For example, no web-server has been provided for the most recent method [18], and hence its usage is quite limited, particularly for the majority of experimental scientists.

The present study was initiated in an attempt to develop a new and more powerful method to identify the SNO sites in proteins in hopes that it may become a useful tool for both basic research and drug development in the relevant areas.

As summarized in [19] and demonstrated in a series of recent publications (see, e.g., [20], [21], [22], [23]), to establish a really useful statistical predictor for a protein or DNA system based on the sequence information, we usually need to consider the following procedures: (i) construct or select a valid benchmark dataset to train and test the predictor; (ii) formulate the protein or DNA sequence samples with a feature vector that can truly reflect the intrinsic correlation with the target to be predicted; (iii) introduce or develop a powerful algorithm (or engine) to operate the prediction; (iv) properly perform cross-validation tests to objectively evaluate the anticipated prediction accuracy; (v) establish a user-friendly web-server for the predictor that is accessible to the public. Below, let us describe how to deal with these procedures one by one.

## Materials and Methods

### 1. Benchmark Dataset

The benchmark dataset used in this study was derived from the dbSNO (http://dbsno.mbc.nctu.edu.tw/), a database that integrates the experimentally verified cysteine SNO sites in 1,757 proteins from different species [24]. To reduce the redundancy and avoid homology bias, we randomly picked 438 proteins in which none had 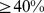 pairwise sequence identity to any other. Based on these proteins and the annotations in the dbSNO database, a total of 731 experimentally verified SNO sites were collected. Meanwhile, to construct a corresponding negative dataset, a total of 810 experimentally verified non-SNO sites were randomly collected from the 438 proteins as well. The corresponding peptide fragments for the 731 SNO sites and 810 non-SNO sites were derived from UniProt database (release 2012_08), as can be generally formulated by
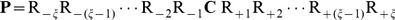
(1)where the subscript 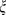 is an integer, 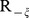 is the 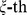 downstream amino acid residue from cysteine (C), 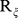 the 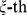 upstream amino acid residue, and so forth. Hereafter let us call a peptide as SNO or non-SNO peptide if its center is a SNO or non-SNO site, respectively. In the current study, we choose 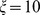. If the upstream or downstream in a protein was less than 10, the lacking residues were filled with the dummy code X. Thus, the benchmark dataset 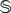 can be formulated as
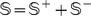
(2)where the positive dataset 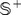 contains 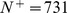 SNO peptide fragments, while the negative dataset 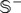
contains 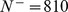 non-SNO peptide fragments (cf. **Eq.1**), respectively. For reader's convenience, their sequences as well as the corresponding sites and protein codes are given in Supporting Information S1.

### 2. Sample Formulation or Feature Vector

To develop a sequence-based predictor for identifying the attribute of a protein or peptide, one of the keys is to formulate its sequence with an effective mathematical expression that can truly reflect the intrinsic correlation with the attribute to be predicted [25]. The most straightforward method to formulate the sample of a protein or peptide is to use its entire amino acid sequence. To identify its attribute, the tools for computing amino acid sequence similarity, such as BLAST [26], [27], were utilized to search the database for those targets that have high sequence similarity to the query protein or peptide. Subsequently, the attribute annotations of the target proteins or peptides thus found were used to infer the attribute for the query protein or peptide. Unfortunately, this kind of straightforward sequential model, although containing the entire sequence information, failed to work when the query protein or peptide did not have any significant sequence similarity to the attribute-known proteins or peptides.

To avoid the above difficulty, which is inherent to the sequential model, various non-sequential or discrete models to formulate protein or peptide samples were proposed in hopes to enhance the prediction power.

Among the discrete models, the simplest one is the amino acid (AA) composition or AAC [28]. However, if using AAC to represent a peptide sample, its sequence-order or position-specific information would be totally lost, and hence might considerably limit the prediction quality.

To avoid completely losing the sequence-order information, the pseudo amino acid composition (PseAAC) was proposed to represent the sample of a protein or peptide [29], [30]. The idea of PseAAC has been widely used in bioinformatics, proteomics, and system biology [25], such as predicting protein structural class [31], predicting metalloproteinase family [32], predicting protein subcellular localization [33], predicting DNA-binding proteins [21], identifying allergenic proteins [34], identify recombination spots [35], identifying bacterial virulent proteins [36], predicting protein folding rate [37], predicting GABA(A) receptor proteins [38], predicting protein supersecondary structure [39], predicting cyclin proteins [40], classifying amino acids [41], predicting enzyme family class [42], identifying risk type of human papillomaviruses [43], identifying protein quaternary structural attributes [44], identifying GPCRs and their types [45], and discriminating outer membrane proteins [46], among many others (see a long list of references cited in [19]). Because of its wide and increasing usage, in 2012 a powerful software called “PseAAC-Builder” (http://www.pseb.sf.net) [47] was established for generating various special modes of PseAAC for protein or peptide sequences.

According to a recent review [19], the general form of PseAAC for a protein or peptide 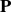 is formulated by
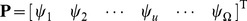
(3)where the subscript 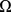 is an integer, and its value as well as the components 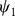, 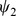, … will depend on how to extract the desired information from the amino acid sequence of 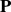 (cf. **Eq.1**). Below, let us describe how to extract useful information from the benchmark dataset 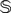 to define the peptide samples concerned via **Eq.3**.

It is obvious from **Eq.1** that when 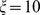, the corresponding peptide contains 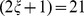 amino acid residues. Since the residue at the center of the sequence is always C, we can omit it. Thus, for the convenience of formulation, **Eq.1** can be reduced to
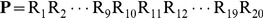
(4)Also, as mentioned above, besides the 20 native amino acids, the sequence may also contain a dummy amino acid X. Here, let us use the numerical codes 1, 2, 3, …, 20 to represent the 20 native amino acids according to the alphabetic order of their single letter codes, and use 21 to represent the dummy amino acid X.

Thus, we can introduce the following 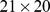 matrix, the so-called “Position Specific Amino Acid Propensity” (PSAAP) matrix [48], to define the components of **Eq.3**

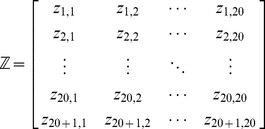
(5)where the element
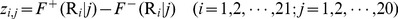
(6)where 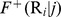 is the occurrence frequency of the 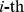 amino acid (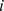 = 1,2,
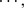
21) in the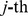 column in the positive benchmark dataset 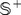 that can be easily derived using the method described in [49] from the sequences in the Supporting Information S1, while 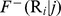 is the corresponding occurrence frequency but derived from the negative benchmark dataset 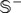.

Thus, the components in **Eq.3** can be uniquely defined by
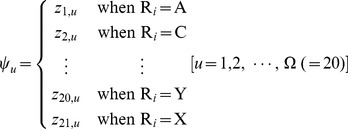
(7)Since the components of the feature vector in **Eq.3** are now derived from the benchmark dataset 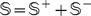, its correlation with SNO sites and non-SNO sites are self-evident.

### 3. Operation Engine

In this study, the “Conditional Random Field” (CRF) algorithm [50] was adopted to operate the prediction. It is a discriminative probabilistic model that inherits the advantages of “Maximum Entropy Markov Models” (MEMMs), often used for labeling and segmenting sequence data. The CRF operation engine has been quite successfully utilized in various areas of bioinformatics and computational proteomics, such as gene prediction [51], SNP array analysis [52], and protein structure [53].

In this study, the CRF software was downloaded from the web-site at http://www.di.ens.fr/~mschmidt/Software/crfChain.html. When used in the current study, the input of CRF is the query peptide fragment 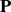 as formulated by the feature vector of **Eq.3** as well as Eqs. 5–7, and the output is 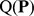, thus the query peptide is identified as
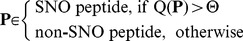
(8)where 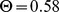 is a threshold obtained by optimizing the overall success rate for the peptides in the benchmark dataset 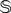 as done in [54].

**Figure 2:**
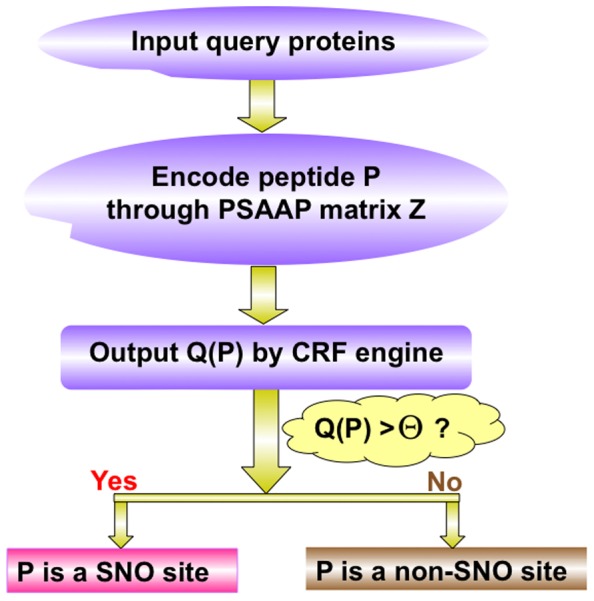
A flowchart to show the prediction process of iSNO-PseAAC.

The predictor thus established via the above procedures is called **iSNO-PseAAC**, which can be used to identify the nitrosylated proteins and their SNO sites. To provide an intuitive picture, a flowchart is provided in **Fig. 2** to illustrate the prediction process of **iSNO-PseAAC**.

## Results and Discussion

### 1. Four Different Metrics for Measuring the Prediction Quality

One of the important procedures in developing a useful statistical predictor [19] is to objectively evaluate its performance or anticipated success rate. To provide a more intuitive and easier-to-understand method to measure the prediction quality, here the criteria proposed in [55] was adopted. According to those criteria, the rates of correct predictions for the SNO peptides in dataset 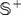 and the non-SNO peptides in dataset 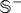 are respectively defined by
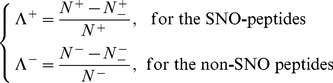
(9)where 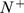 is the total number of the SNO peptides investigated while 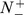 the number of the SNO peptides incorrectly predicted as the non-SNO peptides; 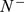 the total number of the non-SNO peptides investigated while 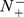 the number of the non-SNO peptides incorrectly predicted as the SNO peptides. The overall success prediction rate is given by [56]

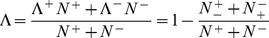
(10)It is obvious from **Eqs. 9** **–** **10** that, if and only if none of the SNO peptides and the non-SNO peptides are mispredicted, i.e., 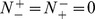 and 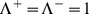, we have the overall success rate 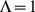. Otherwise, the overall success rate would be smaller than 1.

On the other hand, it is instructive to point out that the following equation is often used in literatures for examining the performance quality of a predictor
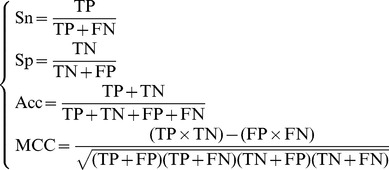
(11)where TP represents the true positive; TN, the true negative; FP, the false positive; FN, the false negative; Sn, the sensitivity; Sp, the specificity; Acc, the accuracy; MCC, the Mathew's correlation coefficient.

The relations between the symbols in **Eq.10** and those in **Eq.11** are given by
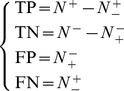
(12)Substituting **Eq.12** into **Eq.11** and also considering **Eq.10**, we obtain
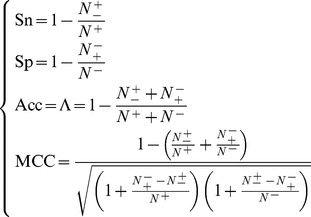
(13)From the above equation, we can see: when 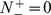 meaning none of the SNO peptides was mispredicted to be a non-SNO peptide, we have the sensitivity 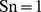; while 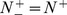 meaning that all the SNO peptides were mispredicted to be the non-SNO peptides, we have the sensitivity 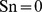. Likewise, when 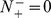 meaning none of the non-SNO peptides was mispredicted, we have the specificity 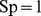; while 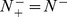 meaning all the non-SNO peptides were incorrectly predicted as the SNO peptides, we have the specificity 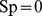. When 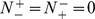 meaning that none of SNO peptides in the dataset 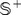
and none of the non-SNO peptides in 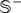 was incorrectly predicted, we have the overall accuracy 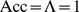; while 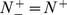 and 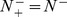 meaning that all the SNO peptides in the dataset 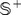 and all the non-SNO peptides in 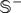 were mispredicted, we have the overall accuracy 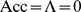. The MCC correlation coefficient is usually used for measuring the quality of binary (two-class) classifications. When 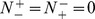 meaning that none of the SNO peptides in the dataset 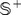 and none of non-SNO peptides in 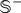 was mispredicted, we have 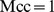; when 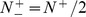 and 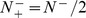 we have 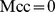 meaning no better than random prediction; when 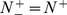 and 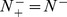 we have 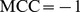 meaning total disagreement between prediction and observation. As we can see from the above discussion, it is much more intuitive and easier-to-understand when using **Eq.13** to examine a predictor for its sensitivity, specificity, overall accuracy, and Mathew's correlation coefficient.

### 2. Cross-Validation to Evaluate Success Rates

In statistical prediction, the following three cross-validation methods are often used to examine a predictor for its effectiveness in practical application: independent dataset test, subsampling (K-fold cross-validation) test, and jackknife test. However, as elaborated in [57] and demonstrated by Eqs.28–32 of [19], among the three cross-validation methods, the jackknife test is deemed the least arbitrary and most objective because it can always yield a unique result for a given benchmark dataset, and hence has been increasingly used and widely recognized by investigators to examine the accuracy of various predictor (see, e.g., [36], [45], [58], [59], [60], [61], [62]). However, to reduce computational time, here let us adopt the 10-fold cross-validation to examine the prediction quality as done by many investigators for PTM sites prediction [63], [64], [65], [66]. The cross-validations were performed 50 times for different subsampling combinations, followed by averaging their outcomes.

The results thus obtained on the benchmark dataset 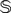 for the four metrics as defined in **Eq.13** are given in **Table 1**, where for facilitating comparison the corresponding results obtained by GPS-SNO [16] are also given. As can be seen from the table, the overall success, sensitivity and MCC rates achieved by **iSNO-PseAAC** are all significantly higher than those by the GPS-SNO predictor [16] regardless its threshold was set at “high”, “medium”, or “low”. As for the method proposed in [17] and the method recently proposed in [18], the former web-server was not working, while the latter had no web-server at all, and hence no corresponding data can be given in **Table 1** for comparison.

**Table 1:** The performance comparison of iSNO-PseAAC with other existing prediction methods^a^ in this area.

Predictor	Sn(%)	Sp(%)	Acc(%)	MCC
iSNO-PseAAC	67.01	68.15	67.62	0.3515
GPS-SNO^b^	18.88	89.63	56.07	0.1210
GPS-SNO^c^	28.04	81.98	56.39	0.1193
GPS-SNO^d^	45.01	73.33	59.90	0.1915

a The method proposed in [18] has no web-server provided, and the web-server in [17] did not work. Therefore, the rates for the two methods are unavailable.

b The method proposed in [16] when the threshold parameter was set “high”.

c The method proposed in [16] when the threshold parameter was set “medium”.

d The method proposed in [16] when the threshold parameter was set “low”.

### 3. Large-Scale Prediction in Identifying Nitrosylated Proteins

Listed in Supporting Information S2 are the predicted results by **iSNO-PseAAC** for a set of 461 independent nitrosylated proteins, none of which occurs in the 438 proteins used to train the current predictor. They were taken from Xue et al. [16] and known belonging to nitrosylated proteins as verified by experiments. As we can see from Supporting Information S3, of the 461 proteins, 416 were predicted containing at least one SNO sites meaning belonging nitrosylated proteins. The overall success rate was 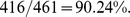


### 4. Web-Server Guide

For the convenience of the vast majority of experimental scientists, a web-server for **iSNO-PseAAC** was established. Below, let us give a step-by-step guide on how to use the web-server to get the desired results without the need to follow the mathematic equations that were presented just for the integrity in developing the predictor.

#### Step 1

**Figure 3:**
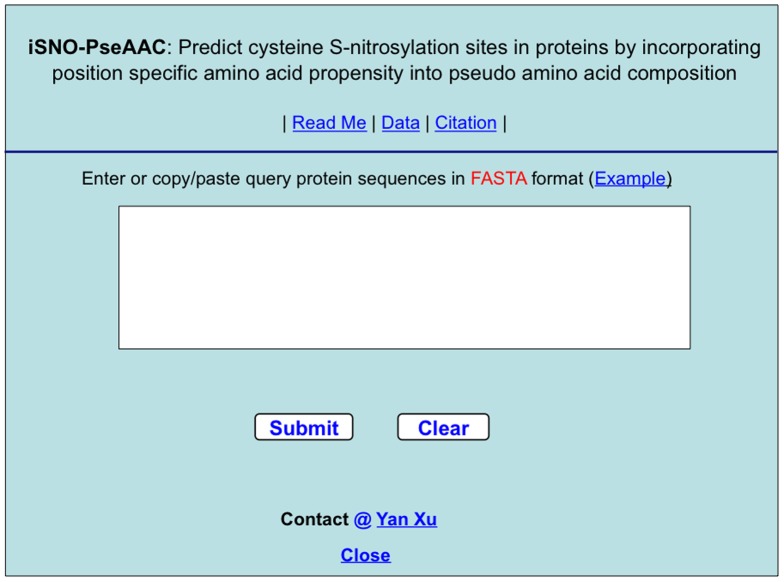
A semi-screenshot to show the top page of the iSNO-PseAAC web-server. Its website address is at http://app.aporc.org/iSNO-PseAAC/.

Open the web server at at http://app.aporc.org/iSNO-PseAAC/ and you will see the top page of the predictor on your computer screen, as show in **Fig. 3**. Click on the Read Me button to see a brief introduction about **iSNO-PseAAC** predictor and the caveat when using it.

#### Step 2

Either type or copy/paste the query protein sequences into the input box shown at the center of **Fig. 3**. The input sequence should be in the FASTA format. A sequence in FASTA format consists of a single initial line beginning with a greater-than symbol (“>”) in the first column, followed by lines of sequence data. The words right after the “>” symbol in the single initial line are optional and only used for the purpose of identification and description. All lines should be no longer than 120 characters and usually do not exceed 80 characters. The sequence ends if another line starting with a “>” appears; this indicates the start of another sequence. Example sequences in FASTA format can be seen by clicking on the Example button right above the input box.

#### Step 3

Click on the Submit button to see the predicted result. For example, if you use the query protein sequences in the Example window as the input, after clicking the Submit button, you will see on your screen the predicted SNO site positions and the corresponding sequences segments as formulated by **Eq.1**. All these results are fully consistent with the experimentally verified results. It takes about a few seconds for the above computation before the predicted results appear on the computer screen; the more number of query proteins and longer of each sequence, the more time it is usually needed.

#### Step 4

Click on the Citation button to find the relevant papers that document the detailed development and algorithm of **iSNO-PseAAC**.

#### Step 5

Click on the Data button to download the benchmark datasets used to train and test the **iSNO-PseAAC** predictor.

#### Caveat

To obtain the predicted result with the expected success rate, the entire sequence of the query protein rather than its fragment should be used as an input. A sequence with less than 50 amino acid residues is generally deemed as a fragment. Also, the size of your input for each submission should be less than 100K; if greater than 100K, please contact Yan Xu at xuyan@ustb.edu.cn.

## Supporting Information

Supporting Information S1The benchmark dataset 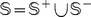, where the positive dataset 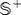 contains 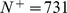 SNO sites while the negative dataset 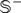 contains 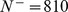 non-SNO sites.(PDF)

Supporting Information S2Predicted results by iSNO-PseAAC on an independent dataset of 461 proteins, which have been verified by experiments as nitrosylated proteins but none of which occurs in the 438 proteins used to train the current predictor. The overall success rate was 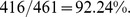

(PDF)

Supporting Information S3 The detailed SNO sites detected by iSNO-PseAAC on an independent dataset with 461 nitrosylated proteins, of which 416 were predicted containing at least one SNO site.
(PDF)
