# Supplementary material for: iSNO-PseAAC: Predict Cysteine S-Nitrosylation Sites in Proteins by Incorporating Position Specific Amino Acid Propensity into Pseudo Amino Acid Composition
Source: PLoS One. 2013 Feb 7;8(2):e55844. doi: 10.1371/journal.pone.0055844 (PMC3567014; doi:10.1371/journal.pone.0055844)
Supplement: Supporting Information S1 — The benchmark dataset , where the positive dataset contains SNO sites while the negative dataset contains non-SNO sites. (PDF) [file pone.0055844.s001.pdf]

Online Supporting Information S1. The benchmark dataset $\mathbb{S} = \mathbb{S}^{+} \cup \mathbb{S}^{-}$, where the positive dataset $\mathbb{S}^{+}$ contains $N^{+} = 731$ SNO sites while the negative dataset $\mathbb{S}^{-}$ contains $N^{-} = 810$ non-SNO sites. See the text of the paper for further explanation.

Positive dataset $\mathbb{S}^{+}$ contains 731 SNO sites and the corresponding peptide sequences

UniProt ID	Site	Sequence (cf. Eq.1 of the paper)
P21980	10	XMAEELVLERCDLELETNGRD
P21980	27	NGRDHHTADLCREKLVVRRGQ
P21980	98	WTATVVDQQDCTLSLQLTTPA
P21980	143	GHFILLFNAWCPADAVYLDSE
P21980	230	VGRVVSGMVNCNDDQGVLLGR
P21980	269	DILRRWKNHGCQRVKYGQCWV
P21980	277	HGCQRVKYGQCWVFAAVACTV
P21980	285	GQCWVFAAVACTVLRCLGIPT
P21980	336	DKSEMIWNFHCWVESWMTRPD
P21980	370	TPQEKSEGTYCCGPVPVRAIK
P21980	371	PQEKSEGTYCCGPVPVRAIKE
P21980	524	VSYNGILGPECGTKYLLNLNL
P21980	545	EPFSEKSVPLCILYEKYRDCL
P21980	620	QNPLPVALEGCTFTVEGAGLT
P21817	35	ATVLKEQLKLCLAAEGFGNRL
P21817	252	RLVYYEGGAVCTHARSLWRLE
P21817	314	SKAHTKATSFCFRISKEKLDV
P21817	810	KFLPPPGYAPCHEAVLPRERL
P21817	905	VRDDNKRLHPCLVDFHSLPEP
P21817	1039	ATKRSNRDSLCQAVRTLLGYG
P21817	1302	LPVQFHQHFRCTAGATPLAPP
P21817	1590	QSERKNPAPQCPPRLEMQMLM
P21817	2326	KGYPDIGWNPCGGERYLDFLR
P21817	2363	VVRLLIRKPECFGPALRGEGG
P21817	3193	VEKLRPALGECLARLAAAMPV
P21817	3635	SKQRRRAVVACFRMTPLYNLP
P29474	94	LSAQAQQDGPCTPRRCLGSLV
P29474	99	QQDGPCTPRRCLGSLVFPRKL
P29474	184	AKQAWRNAPRCVGRIQWGKLQ
P29474	201	GKLQVFDARDCRSAQEMFTYI
P29474	212	RSAQEMFTYICNHIKYATNRG
P29474	661	GLGSRAYPHFCAFARAVDTRL
P29474	802	QYQPGDHIGVCPPNRPGLVEA
P29474	853	GWVRDPRLPPCTLRQALTFFL
P29474	976	DGLGPLHYGVCSTWLSQLKPG
P29474	991	SQLKPGDPVPCFIRGAPSFRL
P29474	1048	QPTPMTLVFGCRCSQLDHLYR
P29474	1050	TPMTLVFGCRCSQLDHLYRDE
P29474	1114	LCLERGHMFVCGDVTMATNVL
P13639	41	GKSTLTDSLVCKAGIIASARA
P13639	67	TDTRKDEQERCITIKSTAISL
P13639	290	PEGKKLPRTFCQLILDPIFKV
P13639	369	PSPVTAQKYRCELLYEGPPDD
P13639	567	CLKDLEEDHACIPIKKSDPVV
P13639	591	ETVSEESNVLCLSKSPNKHNR

P13639	651	WDVAEARKIWCFGPDGTGPNI
P13639	728	GGQIIPTARRCLYASVLTAQP
P17987	76	EVEHPAAKVLCELADLQDKEV
P17987	147	IVNTDELGRDCLINAAKTSMS
P17987	236	PKRIVNAKIACLDFSLQKTKM
P17987	357	QAEEVVQERICDDELILIKNT
P17987	397	EMERSLHDALCVVKRVLESKS
P78527	25	LQETLSAADRCGAALAGHQLI
P78527	478	LAAKGPVLRNCISTVVHQGLI
P78527	795	MQPYYKDILPCLDGYLKTSAL
P78527	1499	KGIAPGDERQCLPSLDLSCKQ
P78527	1507	RQCLPSLDLSCKQLASGLLEL
P78527	2342	ERKNILEESLCELVAKQLKQH
P78527	3187	NIWDDIITNRCFFLSKIEEKL
P78527	3347	IANALSSEPACLAEIEEDKAR
P78527	4045	EKNWYPRQKICYAKRKLAGAN
P10599	62	VIFLEVDVDDCQDVASECEVK
P10599	69	VDDCQDVASECEVKCMPTFQF
P10599	73	QDVASECEVKCMPTFQFFKKG
P98170	12	TFNSFEGSKTCVPADINKEEE
P98170	90	VGRHRKVSPNCRFINGFYLEN
P98170	213	CGGKLKNWEPCDRAWSEHRRH
P98170	300	YALGEGDKVKCFHCGGGLTDW
P98170	303	GEGDKVKCFHCGGGLTDWKPS
P98170	327	WEQHAKWYPGCKYLLEQKGQE
P98170	351	NIHLTHSLEECLVRTTEKTPS
Q15149	950	MRGRLPLLAVCDYKQVEVTVH
Q15149	1098	RLMAEREYGSCSHHYQQLLQS
Q15149	1136	LKDIRLQLEACETRTVHRLRL
Q15149	3295	KSHRVPLDVACARGCLDEETS
Q15149	3299	VPLDVACARGCLDEETSRALS
Q15149	3336	PATYGELQQRCRPDQLTGLSL
Q15149	3667	NLTYRQLLERCVEDPETGLRL
P10809	237	YFINTSKGQKCEFQDAYVLLS
P10809	442	VEEGIVLGGGCALLRCIPALD
P10809	447	VLGGGCALLRCIPALDSLTPA
P68366	54	GGDDSFTTFFCETGAGKHVPR
P68366	295	QLSVAEITNACFEPANQMVKC
P68366	347	TKRSIQFVDWCPTGFKVGINY
P68366	376	GDLAKVQRAVCMLSNTTAIAE
P13010	235	YSFSESLRKLCVFKKIERHSI
P13010	249	KIERHSIHWPCRLTIGSNLSI
P13010	296	KEDIQKETVYCLNDDDETEVL
P13010	339	EQMKYKSEGKCFSVLGFCKSS
P13010	346	EGKCFSVLGFCKSSQVQRRFF
P13010	493	PNPRFQRLFQCLLHRALHPRE
Q00610	151	MFDRHSSLAGCQIINYRTDAK
Q00610	491	RANVPNKVIQCFAETGQVQKI
Q00610	736	DVHFKYIQAACKTGQIKEVER
Q00610	918	YCEKRDPHLACVAYERGQCDL
Q00610	926	LACVAYERGQCDLELINVCNE
Q00610	934	GQCDLELINVCNENSLFKSLS
P00533	190	FQNHLGSCQKCDPSCPNGSCW
P00533	291	PEGKYSFGATCVKKCPRNYVV
P00533	311	VTDHGSCVRACGADSYEMEED

P00533	329	EEDGVRKCKKCEGPCRKVCNG
P00533	470	DVIISGNKNLCYANTINWKKL
P07237	53	YLLVEFYAPWCGHCKALAPEY
P07237	56	VEFYAPWCGHCKALAPEYAKA
P07237	312	LEFFGLKKEECPAVRLITLEE
P07237	397	NVFVEFYAPWCGHCKQLAPIW
P07237	400	VEFYAPWCGHCKQLAPIWDKL
P27348	25	QAERYDDMATCMKAVTEQGAE
P27348	94	EKVESELRSICTTVLELLDKY
P27348	134	DYFRYLAEVACGDDRKQTIDN
P00558	50	IKAAVPSIKFCLDNGAKSVVL
P00558	99	LGKDVLFLKDCVGPEVEKACA
P00558	108	DCVGPEVEKACANPAAGSVIL
P21333	444	LEARGDSTYRCSYQPTMEGVH
P21333	717	LRVQVQDNEGCPVEALVKDNG
P21333	810	GQGDVSIGIKCAPGVVGPAEA
P21333	1260	EPAVDTSGVQCYGPGIEGQGV
P21333	2543	VFVDSLTKATCAPQHGAPGPG
P49327	634	GLSWEECKQRCPPGVVPACHN
P49327	642	QRCPPGVVPACHNSKDTVTIS
P49327	1118	EQQVPILEKFCFTPHTEEGCL
P49327	1127	FCFTPHTEEGCLSERAALQEE
P49327	1471	RREPGGNRLRCVLLSNLSSTS
P63244	138	TIKLWNTLGVCKYTVQDESHS
P63244	168	PNSSNPIIVSCGWDKLVKVWN
P63244	182	KLVKVWNLANCKLKTNHIGHT
P63244	249	LCFSPNRYWLCAATGPSIKIW
P06213	1083	NEASVMKGFTCHHVVRLLGVV
P06213	1165	FVHRDLAARNCMVAHDFTVKI
P06213	1261	DGGYLDQPDNCPERVTDLMRM
P06213	1272	PERVTDLMRMCWQFNPKMRPT
P07900	420	KVIRKNLVKKCLELFTELAED
P07900	481	GDEMVSLKDYCTRMKENQKHI
P07900	597	VVSNRLVTSPCCIVTSTYGWT
P07900	598	VSNRLVTSPCCIVTSTYGWTA
P18031	32	DIRHEASDFPCRVAKLPKNKN
P18031	92	ILTQGPLPNTCGHFWEMVWEQ
P18031	215	SPEHGPVVVHCSAGIGRSGTF
P35579	91	SKVEDMAELTCLNEASVLHNL
P35579	569	PKQLKDKADFCIIHYAGKVDY
P35579	896	QEQLQAETELCAEAEELRARL
P35579	917	TAKKQELEEICHDLEARVEEE
P46459	21	CPTDELSLTNCAVVNEKDFQS
P46459	91	SLYTFDKAKQCIGTMTIEIDF
P46459	250	FPPEIVEQMGCKHVKGILLYG
P46459	264	KGILLYGPPGCGKTLLARQIG
P12814	332	LHKPPKVQEKCQLEINFNTLQ
P12814	480	PSVNARCQKICDQWDNLGALT
P62826	112	PNWHRDLVRVCENIPIVLCGN
P62826	120	RVCENIPIVLCGNKVDIKDRK
P62736	219	EIVRDIKEKLCYVALDFENEM
P62736	259	VITIGNERFRCPETLFQPSFI
P62736	287	HETTYNSIMKCDIDIRKDLYA
P62736	376	EAGPSIVHRKCFXXXXXXXXX
P04350	12	REIVHLQAGQCGNQIGAKFWE

P04350	239	VSATMSGVTTCLRFPGQLNAD
P04350	303	MFDAKNMMAACDPRHGRYLTV
P04350	354	WIPNNVKTAVCDIPPRGLKMA
P55072	105	RLGDVISIQPCPDVKYGKRIH
P55072	535	TLLAKAIANECQANFISIKGP
P55072	572	IFDKARQAAPCVLFFDELDSI
P34932	34	ETIANEYSDRCTPACISFGPK
P34932	38	NEYSDRCTPACISFGPKNRSI
P34932	245	KFDEVLVNHFCEEFGKKYKLD
P34932	417	NSPAEEGSSDCEVFSKNHAAP
P31943	34	ADEVQRFFSDCKIQNGAQGIR
P31943	122	FVRLRGLPFGCSKEEIVQFFS
P31943	267	SDRFGRDLNYCFSGMSDHRYG
Q99832	364	GGERYNFFTGCPKAKTCTFIL
Q99832	450	KALEIIPRQLCDNAGFDATNI
P14866	260	SLNGADIYSGCCTLKIEYAKP
P14866	261	LNGADIYSGCCTLKIEYAKPT
P14866	452	NFMFGQKLNVCVSKQPAIMPG
P14866	581	NGPYPYTLKLCFSTAQHASXX
P50991	295	NLVKQIKKTGCNVLLIQKSIL
P50991	379	GSGKLLKITGCASPGKTVTIV
P50991	410	EAERSIHDALCVIRCLVKKRA
P04075	73	LLTADDRVNPCIGGVILFHET
P04075	240	KPNMVTPGHACTQKFSHEEIA
P04075	339	VKRALANSLACQGKYTPSGQA
P14618	152	ITLDNAYMEKCDENILWLDYK
P14618	423	AVGAVEASFKCCSGAIIVLTK
P14618	424	VGAVEASFKCCSGAIIVLTKS
P14618	474	HLYRGIFPVLCKDPVQEAWAE
P36873	127	NFFLLRGNHECASINRIYGFY
P36873	155	NIKLWKTFTDCFNCLPIAAIV
P36873	158	LWKTFTDCFNCLPIAAIVDEK
P36873	245	FLHKHDLDLICRAHQVVEDGY
P15121	299	LLSYNRNWRVCALLSCTSHKD
P15121	304	RNWRVCALLSCTSHKDYPFHE
P31749	224	KYSFQTHDRLCFVMEYANGGE
P31749	296	GHIKITDFGLCKEGIKDGATM
P31749	310	IKDGATMKTFCGTPEYLAPEV
P35754	8	XXXMAQEFVNCKIQPGKVVVF
P35754	79	TVPRVFIGKDCIGGCSDLVSL
P35754	83	VFIGKDCIGGCSDLVSLQQSG
P38646	487	DGQTQVEIKVCQGEREMAGDN
P38646	608	EFKDQLPADECNKLKEEISKM
P50570	27	QDAFSSIGQSCHLDLPQIAVV
P50570	86	SKTEHAEFLHCKSKKFTDFDE
P50570	607	YKDLRQIELACDSQEDVDSWK
P50995	294	AIKGVGTDEACLIEILASRSN
P50995	384	TDESKFNAVLCSRSRAHLVAV
Q12879	87	TDPKSLITHVCDLMSGARIHG
Q12879	320	FSYIPEAKASCYGQMERPEVP
Q12879	399	VWPRYKSFSDCEPDDNHLSIV
O00429	367	TSELCGGARICYIFHETFGRT
O00429	644	RKLSAREQRDCEVIERLIKSY
Q06830	52	FFYPLDFTFVCPTEIIAFSDR
Q06830	173	FQFTDKHGEVCPAGWKPGSDT

O43175	281	ALVDHENVISCPHLGASTKEA
O43175	369	QGTSLKNAGNCLSPAVIVGLL
P36578	96	RSGQGAFGNMCRGGRMFAPTK
P36578	208	NRRRIQRRGPCIIYNEDNGII
P36578	250	LAPGGHVGRFCIWTESAFRKL
O95573	450	LLGGNIRLLLCGGAPLSATTQ
O95573	561	FEDENGQRWLCTGDIGEFEPD
O95573	573	GDIGEFEPDGCLKIIDRKKDL
P09382	43	LNLGKDSNNLCLHFNPRFNAH
P09382	61	NAHGDANTIVCNSKDGGAWGT
P30050	17	PNEIKVVYLRCTGGEVGATSA
P30050	141	EILGTAQSVGCNVDGRHPHDI
P30050	162	IDDINSGAVECPASXXXXXXX
P46782	155	RRVNQAIWLLCTGAREAAFRN
P46782	172	AFRNIKTIAECLADELINAAK
P61247	139	TTDGYLLRLFCVGFTKKRNNQ
P61247	201	DSIGKDIEKACQSIYPLHDVF
P62888	52	KAKLVILANNCPALRKSEIEY
P62888	92	GTACGKYYRVCTLAIIDPGDS
P63167	24	SEEMQQDSVECATQALEKYNI
P63167	56	FDKKYNPTWHCIVGRNFGSYV
P62879	25	RNQIRDARKACGDSTLTQITA
P62879	204	PDGRTFVSGACDASIKLWDVR
P62879	317	VLAGHDNRVSCLGVTDDGMAV
P61081	47	DINELNLPKTCDISFSDPDDL
P61081	65	DDLLNFKLVICPDEGFYKSGK
P11142	574	DEDKQKILDKCNEIINWLDKN
P11142	603	EHQQKELEKVCNPIITKLYQS
P52272	114	LMDAEGKSRGCAVVEFKMEES
P52272	676	WKMLKDKFNECGHVLYADIKM
P52272	694	IKMENGKSKGCGVVKFESPEV
O75369	604	IEYNDQNDGSCDVKYWPKEPG
O75369	660	AYGPGLEKSGCIVNNLAEFTV
O75369	1326	SPFKVAVTEGCQPSRVQAQGP
Q92945	176	QINKIQQDSGCKVQISPDSGG
Q92945	436	EMTFSIPTHKCGLVIGRGGEN
P23229	86	RANRTGGLYSCDITARGPCTR
P23229	131	SQGPGGKVVTCAHRYEKRQHV
P23229	541	PSGICLQVKSCFEYTANPAGY
Q09666	1833	EAEVPDVDLECPDAKLKGPKF
Q09666	1900	GVEVPDVELECPDAKLKGPKF
Q09666	2806	DVSGPKVDVECPDVNIEGPEG
O00170	208	AAAKYYDAIACLKNLQMKEQP
O00170	238	QQITPLLLNYCQCKLVVEEYY
O00170	240	ITPLLLNYCQCKLVVEEYYEV
Q96EK6	113	LIIEHKFIHSCAKRGRVEDVV
Q96EK6	128	RVEDVVVSDECRGKQLGKLLL
Q96EK6	157	KLNCYKITLECLPQNVGFYKK
Q96AG4	48	LPKATILDLSCNKLTTLPSDF
Q96AG4	59	NKLTTLPSDFCGLTHLVKLDL
Q96AG4	131	DPVLAKVAGDCLDEKQCKQCA
Q09161	73	NYKSKILRLLCTVARLLPEKL
Q09161	477	DIVPPTFSALCPANPTCIYKY
Q09161	483	FSALCPANPTCIYKYGDESSN
Q15366	54	GARINISEGNCPERIITLAGP

Q15366	109	TLRLVVPASQCGSLIGKGGCK
Q15366	158	IAGIPQSIIECVKQICVVMLE
P20618	82	YKLTDKTVIGCSGFHGDCLTL
P20618	89	VIGCSGFHGDCLTLTKIIEAR
P20618	224	DVYTGDALRICIVTKEGIREE
O43765	129	ELNPANAVYFCNRAAAYSKLG
O43765	148	LGNYAGAVQDCERAICIDPAY
O43765	153	GAVQDCERAICIDPAYSKAYG
P07814	92	LEFSATKLSSCDSFTSTINEL
P07814	105	FTSTINELNHCLSLRTYLVGN
P07814	856	PKAKINEAVECLLSLKAQYKE
P50990	148	KAHEILPNLVCCSAKNLRDID
P50990	149	AHEILPNLVCCSAKNLRDIDE
P50990	430	AKQITSYGETCPGLEQYAIKK
Q15185	40	NFEKSKLTFSCLGGSDNFKHL
Q15185	75	KHKRTDRSILCCLRKGESGQS
Q15185	76	HKRTDRSILCCLRKGESGQSW
P55060	344	FEDQNTLTSICEKVIVPNMEF
P55060	387	SDIDTRRRAACDLVRGLCKFF
P55060	939	AQSLHKLSTACPGRVPSMVST
P27695	65	PSGKPATLKICSWNVDGLRAW
P27695	93	WVKEEAPDILCLQETKCSENK
P27695	310	IRSKALGSDHCPITLYLALXX
O14920	179	YAKELDQGSLCTSFVGTLQYL
O14983	344	RSLPSVETLGCTSVICSDKTG
O14983	349	VETLGCTSVICSDKTGTLTTN
O15519	254	YKMKSKPLGICLIIDCIGNET
O15519	259	KPLGICLIIDCIGNETELLRD
O94760	222	LTVPDDIAANCIYLNIPNKGH
O94760	274	ELEKVDGLLTCCSVLINKKVD
P00403	196	ATRPGVYYGQCSEICGANHSF
P00403	200	GVYYGQCSEICGANHSFMPIV
P05089	168	DVPGFSWVTPCISAKDIVYIG
P05089	303	VNTAVAITLACFGLAREGNHK
P08670	328	EYRRQVQSLTCEVDALKGTNE
P10415	158	VAFFEFGGVMCVESVNREMSP
P10415	229	TLLSLALVGACITLGAYLGHK
P19838	61	KQRGFRFRYVCEGPSHGGLPG
P22460	331	ADPFFIVETTCVIWFTFELLV
P22460	346	TFELLVRFFACPSKAGFSRNI
P35247	35	TYSHRTMPSACTLVMCSSVES
P35247	40	TMPSACTLVMCSSVESGLPGR
P48039	127	NITGIAINRYCYICHSLKYDK
P48039	130	GIAINRYCYICHSLKYDKLYS
P68104	234	SGTTLLEALDCILPPTRPTDK
P68104	411	IVDMVPGKPMCVESFSDYPPL
Q05586	744	AVLEFEASQKCDLVTTGELFF
Q05586	798	LDKTWVRYQECDSRSNAPATL
Q16665	520	SPEPNSPSEYCFYVDSDMVNE
Q16665	800	SGLPQLTSYDCEVNAPIQGSR
P02730	201	QHSSLETQLFCEQGDGGTEGH
P02730	317	LLHSLEGFLDCSLVLPPTDAP
P07355	133	TDEDSLIEIICSRTNQELQEI
Q92769	262	MYQPSAVVLQCGADSLSGDRL
Q92769	274	ADSLSGDRLGCFNLTVKGHAK

Q99836	216	SIASELIEKRCRRMVVVVSDD
Q9UL62	553	ETRAIDEPNNCKGIRCEKQNN
Q9UL62	558	DEPNNCKGIRCEKQNNAFSTL
Q9UQM7	6	XXXXXMATITCTRFTEEYQLF
Q9UQM7	280	WISHRSTVASCMHRQETVDCL
Q9Y272	11	MKLAAMIKKMCPSDSELSIPA
P30046	24	RVPAGLEKRLCAAAASILGKP
P31949	13	KISSPTETERCIESLIAVFQK
P43243	806	IPKTGFYCKLCSLFYTNEEVA
P45880	47	LVKLDVKTKSCSGVEFSTSGS
P45880	76	TGTLETKYKWCEYGLTFTEKW
Q9BUJ2	377	ALYPHVLVKNCAVEFNFGQRA
Q9BUJ2	487	WDVLIQQATQCLNRLIQIAAR
P62070	55	PTIEDSYTKQCVIDDRAARLD
P62070	183	RVIRKFQEQECPPSPEPTRKE
P62701	41	PSTGPHKLRECLPLIIFLRNR
P62701	181	DFIKFDTGNLCMVTGGANLGR
Q9NQC3	1101	YSNSALGHVNCTIKELRRLFL
O75390	211	DSMDLIAKLPCVAAKIYRNLY
P04083	324	FYQKMYGISLCQAILDETKGD
P04083	343	GDYEKILVALCGGNXXXXXXX
P46777	76	YARIEGDMIVCAAYAHELPKY
P46777	100	VGLTNYAAAYCTGLLLARRLL
O43707	499	HNVNTRCQKICDQWDALGSLT
O43707	793	GALGPEEFKACLISLGYDVEN
Q99439	61	QKGLKDGTILCTLMNKLQPGS
Q99439	164	FDDATMKAGQCVIGLQMGTNK
P04843	477	PAAEARMKVACITEQVLTLVN
Q9P2E9	892	KRLDEVSRELCHTQSSHASLR
Q9P2E9	1323	AEFEEAQTSACRLQEELEKLR
P04406	152	NSLKIISNASCTTNCLAPLAK
P04406	247	TANVSVVDLTCRLEKPAKYDD
P20810	408	AAPAPVSEAVCRTSMCSIQSA
P20810	661	IDALSGDLDSCPSTTETSQNT
P13489	38	QQCQVVRLDDCGLTEARCKDI
P13489	248	KLGDVGMAELCPGLLHPSSRL
P22314	234	VTKDNPGVVTCLDEARHGFES
P22314	632	QDPPEKSIPICTLKNFPNAIE
Q08211	12	GDVKNFLYAWCGKRKMTPSYE
Q08211	1029	NALIHKSSVNCPFSSQDMKYP
P62258	97	QMVETELKLICCDILDVLDKH
P62258	98	MVETELKLICCDILDVLDKHL
P63104	94	EKIETELRDICNDVLSLLEKF
P63104	189	YEILNSPEKACSLAKTAFDEA
P50579	436	SKYLMALKNLCDLGIVDPYPP
P50579	448	LGIVDPYPPLCDIKGSYTAQF
O15382	342	VREVFGSGTACQVCPVHRILY
O15382	345	VFGSGTACQVCPVHRILYKDR
Q86VP6	571	ATPYIKDLFTCTIKRLKAADI
Q86VP6	1134	FLMLVRLSTLCPSAVLQRLDR
O75828	226	RKADRILVNACCPGPVKTDMD
O75828	227	KADRILVNACCPGPVKTDMDG
Q16543	308	YESLPEELQKCFDVKDVQMLQ
Q16543	336	PTDAKYHMQRCIDSGLWVPNS
Q92879	61	RSQNPPQSKGCCFVTFYTRKA

Q92879	62	SQNPPQSKGCCFVTFYTRKAA
Q8TCG1	92	QLAVDIETRDCLQNTYNLNSV
Q8TCG1	108	NLNSVLAGVVCRSSHTDSVFL
Q9UBF2	296	APAVSVLQLFCSSPKPALRYA
Q9UBF2	325	AMKHPSAVTACNLDLENLITD
Q9H9Q2	110	TIVSLASRMKCIPYSVLLKDL
Q9H9Q2	240	EMEQQLAERECPPHAEQRQPT
P17844	200	YCRACRLKSTCIYGGAPKGPQ
P17844	234	TPGRLIDFLECGKTNLRRTTY
Q9NR33	84	FVETIAKDAYCCAQQGKRKTL
Q9NR33	85	VETIAKDAYCCAQQGKRKTLQ
P43897	71	FVNCKKALETCGGDLKQAEIW
P43897	240	VLGKYGALVICETSEQKTNLE
P30042	176	FHQAGKPIGLCCIAPVLAAKV
P30042	177	HQAGKPIGLCCIAPVLAAKVL
P10768	176	YKSVSAFAPICNPVLCPWGKK
P10768	181	AFAPICNPVLCPWGKKAFSGY
P21266	39	DTSYEEKRYTCGEAPDYDRSQ
P21266	208	IAAYLQSDQFCKMPINNKMAQ
P49915	449	PGPGLAIRVICAEEPYICKDF
P49915	456	RVICAEEPYICKDFPETNNIL
O94927	349	RQVLILGLRRCCLWTELKALH
O94927	350	QVLILGLRRCCLWTELKALHD
Q14103	126	YFSKFGEVVDCTLKLDPITGR
Q14103	226	DNKTNKRRGFCFITFKEEEPV
P61978	184	TQTTIKLFQECCPHSTDRVVL
P61978	185	QTTIKLFQECCPHSTDRVVLI
Q8TEX9	42	LRAPAALPALCDLLASAADPQ
Q8TEX9	708	VFEEVFKLLECPHLNVRKAAH
P40926	212	GKTIIPLISQCTPKVDFPQDQ
P40926	285	CSFVKSQETECTYFSTPLLLG
P28482	65	KISPFEHQTYCQRTLREIKIL
P28482	166	LLNTTCDLKICDFGLARVADP
P54886	606	KVTRLVRDSKCEYPAACNALE
P54886	612	RDSKCEYPAACNALETLLIHR
P12004	81	NLTSMSKILKCAGNEDIITLR
P12004	162	SHIGDAVVISCAKDGVKFSAS
Q29RF7	1079	SKTNEKLYTVCDVALCVINSK
Q29RF7	1084	KLYTVCDVALCVINSKSALCN
Q9Y3B4	74	YEDIFDAKNACDHLSGFNVCN
Q9Y3B4	83	ACDHLSGFNVCNRYLVVLYYN
Q9NPH0	175	NIFRNLESTRCLLAGLFQCQK
Q9NPH0	183	TRCLLAGLFQCQKEGPIIIHT
Q08752	275	RAKLQPIALSCVLNIGACKLK
Q08752	282	ALSCVLNIGACKLKMSNWQGA
P60900	154	DEEQGPQVYKCDPAGYYCGFK
P60900	161	VYKCDPAGYYCGFKATAAGVK
O00231	202	SARTTANAIYCPPKLQATLDM
O00231	289	YAGRQTEALKCVAQASKNRSL
P55036	37	QAQQDAVNIVCHSKTRSNPEN
P55036	87	TVQPKGKITFCTGIRVAHLAL
P26599	250	SLDGQNIYNACCTLRIDFSKL
P26599	251	LDGQNIYNACCTLRIDFSKLT
Q06203	339	TPAALAYAGKCGLPYVEVLCK
Q06203	348	KCGLPYVEVLCKNRYVGRTFI

Q92600	91	TLTAHQSNRVCNALALLQCVA
Q92600	99	RVCNALALLQCVASHPETRSA
P27635	71	SSEALEAARICANKYMVKSCG
P27635	195	VAEKRLIPDGCGVKYIPNRGP
P62913	72	IRRNEKIAVHCTVRGAKAEEI
P62913	150	SIADKKRRTGCIGAKHRISKE
P50914	42	DQNRALVDGPCTQVRRQAMPF
P50914	54	QVRRQAMPFKCMQLTDFILKF
Q8NHW5	27	IIQLLDDYPKCFIVGADNVGS
Q8NHW5	119	AAARAGAIAPCEVTVPAQNTG
Q8N0X7	499	KVSQFLVDGVCTVANCVGKEL
Q8N0X7	504	LVDGVCTVANCVGKELAPHVK
P49458	39	LKYRHSDGNLCVKVTDDLVCL
P49458	48	LCVKVTDDLVCLVYKTDQAQD
P31948	26	SVGNIDDALQCYSEAIKLDPH
P31948	62	GDYQKAYEDGCKTVDLKPDWG
Q15527	29	RLQTDARKVRCILTGHELPCR
Q15527	38	RCILTGHELPCRLPELQVYTR
P41250	466	IEIVGCADRSCYDLSCHARAT
P41250	471	CADRSCYDLSCHARATKVPLV
P54577	250	DVKKKLKKAFCEPGNVENNGV
P54577	501	LQADFKISEECIAQWKQTNFM
P48643	181	TTLGSKVVNSCHRQMAEIAVN
P48643	407	EAKRSLHDALCVIRNLIRDNR
P49368	173	KAISRWSSLACNIALDAVKMV
P49368	213	KIPGGIIEDSCVLRGVMINKD
O95801	367	VKALTPAFLVCVGSSPFCKNF
O95801	374	FLVCVGSSPFCKNFLRGRKVY
P09936	152	AHDAVAQEGQCRVDDKVNFHF
P09936	220	GEVRFSAVALCKAAXXXXXXX
P21283	225	VLSEDQDSYLCNVTLFRKAVD
P21283	376	YYPYVYYKIDCNLLEFKXXXX
O00220	336	VTVQSPGEAQCLLGPAEAEGS
P00750	118	CQCPEGFAGKCCEIDTRATCY
P00966	132	GNDQVRFELSCYSLAPQIKVI
P01009	256	KRLGMFNIQHCKKLSSWVLLM
P01111	118	DVPMVLVGNKCDLPTRTVDTK
P02766	10	XMASHRLLLLCLAGLVFVSEA
P04271	85	MAFVAMVTTACHEFFEHEXXX
P05062	269	RTVPAAVPGICFLSGGMSEED
P05109	42	DDLKKLLETECPQYIRKKGAD
P05976	181	LMAGQEDSNGCINYEAFVKHI
P10242	130	KHLKGRIGKQCRERWHNHLNP
P10620	50	TRKVFANPEDCVAFGKGENAK
P11926	360	YYSSSIWGPTCDGLDRIVERC
P14780	99	ATLKAMRTPRCGVPDLGRFQT
P20337	184	QAFERLVDAICDKMSDSLDTD
P23284	202	KPLKDVIIADCGKIEVEKPFA
P23975	351	RDALLTSSINCITSFVSGFAI
P27816	635	PETVTGTGKKCSLPAEEDSVL
P29350	453	LPHAGPIIVHCSAGIGRTGTI
P30556	289	IVDTAMPITICIAYFNNCLNP
P35557	371	TTDCDIVRRACESVSTRAAHM
P40227	406	RAVKNAIDDGCVVPGAGAVEV
P42574	163	GKPKLFIIQACRGTELDCGIE

P43235	139	PVKNQGQCGSCWAFSSVGALE
P45983	116	IVMELMDANLCQVIQMELDHE
P47712	151	CPDLRFSMALCDQEKTFRQQR
P48200	178	CRGQTTCRGSCDSGELGRNSG
P51787	445	EKMLTVPHITCDPPEERRLDH
P68036	86	HPNIDEKGQVCLPVISAENWK
P69905	105	DPVNFKLLSHCLLVTLAAHLP
Q00987	77	YDEKQQHIVYCSNDLLGDLFG
Q04206	38	KQRGMRFRYKCEGRSAGSIPG
Q16623	145	NATQSDYRERCKGRIQRQLEI
P18206	85	PPAFIKVENACTKLVQAAQML
P53621	245	NESKAWEVDTCRGHYNNVSCA
Q8WX93	964	TANQEYKVSSCEQRLISEIEY
Q99683	869	GKAADIWSLGCTIIEMATGKP
Q9GZT4	113	YIVVPQTAPDCKKLAIQAYGA
Q9Y696	234	AYSRDEFTNTCPSDKEVEIAY
P23297	86	VVLVAALTVACNNFFWENSXX
P14854	54	MTAKGGDISVCEWYQRVYQSL
P26641	194	FPNTNRWFLTCINQPQFRAVL
P30048	229	FQYVETHGEVCPANWTPDSPT
P31327	600	YALGGLGSGICPNRETLMDLS
P41567	69	LVKAFKKKFACNGTVIEHPEY
P62241	100	LVRTKTLVKNCIVLIDSTPYR
P62280	131	QIGDIVTVGECRPLSKTVRFN
Q8NBI5	226	PLPPNYSYGLCPGNGTTKEEK
P61163	34	GFAGDQIPKYCFPNYVGRPKH
Q8NBU5	137	KGVLLYGPPGCGKTLIAKATA
Q13557	373	NGDFEAYTKICDPGLTAFEPE
Q9NX47	46	RGSTKWVHQACLQRWVDEKQR
P84095	157	KQIHAVRYLECSALQQDGVKE
O14979	177	YLSRFGEVVDCTIKTDPVTGR
O43390	292	PDDKKKNRGFCFLEYEDHKSA
P30101	244	KFIQENIFGICPHMTEDNKDL
A6NMY6	133	TDEDSLIEIICSRTNQELQEI
O15355	13	AYLSQPNTVKCSGDGVGAPRL
P67936	154	EERAEVSELKCGDLEEELKNV
O00299	191	LPKLHIVQVVCKKYRGFTIPE
P23396	97	YAEKVATRGLCAIAQAESLRY
P30044	100	FGVPGAFTPGCSKTHLPGFVE
P49411	290	ERGILKKGDECELLGHSKNIR
O60443	156	QVLEGRNEVLCVLTQKITTMQ
Q9Y277	65	SGNLETKYKVCNYGLTFTQKW
P78347	475	KLRKMVDQLFCKKFAEALGST
Q07065	100	AAAAASSSASCSRRLGRALNF
Q8TAQ2	145	NCLSRPNIFLCPEIEPKLLGK
Q14498	478	NSAQGNVYVKCPSIAAAIAAV
P16615	560	EWGSGSDTLRCLALATHDNPL
Q14315	1103	GGLGLTVEGPCEAKIECQDNG
P09651	43	HFEQWGTLTDCVVMRDPNTKR
P41091	236	AQLKYNIEVVCEYIVKKIPVP
P42704	930	SARLQWFCDRCVANNQVETLE
P30153	390	VRLNIISNLDCVNEVIGIRQL
P17174	46	LGVGAYRTDDCHPWVLPVVKK
P00505	187	QGYRYYDPKTCGFDFTGAVED
P53396	229	AKVDATADYICKVKWGDIEFP

Q99798	592	LQILIKVKGKCTTDHISAAGP
P55263	160	SLIANLAAANCYKKEKHLDLE
P05141	160	AEREFRGLGDCLVKIYKSDGI
O00468	405	CPEPCRFNAVCLSRRGRPRCS
O95433	207	ARPVGVKIPTCKITLKETFLT
Q12904	161	DVSRLDLRIGCIITARKHPDA
Q13155	143	LSLLVLHRLLCEHFRVLSTVH
P14550	134	PFPKNADGTICYDSTHYKETW
Q01433	230	QRVTISGEEKCGVPFTDLLDA
P39687	123	ENLKSLDLFNCEVTNLNDYRE
Q99873	262	VEDLTFTSPFCLQVKRNDYVH
O14744	22	SRVSSGRDLNCVPEIADTLGA
Q9NVM4	262	FSKQVSSSAACHSRRFEPLTS
P08133	114	AISGIGTDEKCLIEILASRTN
P20073	298	TNQEIREIVRCYQSEFGRDLE
P36405	118	AELLEEEKLSCVPVLIFANKQ
Q9H0F7	148	VTSVKVSQLLCLENIKDKPWH
Q6NXE6	484	VAKAALRDLGCHVELRELWTG
P17405	120	ARVGSVAIKLCNLLKIAPPAV
Q99700	301	EGVFKTYSPKCDLVLDAAHEK
O95816	162	DQKFQSIVIGCALEDQKKIKR
O95861	243	ASAYVFASPGCKKWDTCAPEV
Q01518	93	ERALLVTASQCQQPAENKLSD
Q14444	226	DLLEGKEKPVCGTTYKVLKEI
P42772	74	LLLLHGAEPNCADPATLTRPV
Q4G0N4	393	NRVFSSSRQRCFSSKVCVRSR
Q9P1F3	39	KFGVLFRDDKCANLFEALVGT
Q6P1X6	98	YEAAFPFLSPCGRERNFLRCE
Q9UNE7	199	DDSHVRAQQACIEAKHDKYMA
Q15417	59	QLGLKDGIILCELINKLQPGS
P23528	147	ANCYEEVKDRCTLAEKLGGSA
P53618	248	NPSERARFIRCIYNLLQSSSP
P48444	441	HDSRRNTLEWCLPVIDAKNKS
Q7L5N1	143	PSDIHVHKQVCEIIESPLFLK
O60716	394	SNAAAYLQHLCYRNDKVKTDV
Q96EP5	124	KIFVGGIPHNCGETELREYFK
P61962	109	RVGETETRLECLLNNNKNSDF
Q9BW61	25	NFSRFHADSVCKASNRRPSVY
O15523	315	GQQIRDLERGCHLLVATPGRL
P26196	324	AYVTERQKVHCLNTLFSRLQI
P60981	147	ANGPEDLNRACIAEKLGGSLI
Q3LXA3	404	ALDRAAGDGDCGTTHSRAARA
O43143	190	SLPGPKRGVACTQPRRVAAMS
O60610	1227	GPRQANRKAGCAVTSLLASEL
Q9UDY4	175	RVSLEEIYSGCTKRMKISRKR
Q99615	175	MDRALEFAPACHRFKILKAEC
Q14181	198	AGNISLKVLGCPEALTGSYKS
Q16555	504	VPRGLYDGPVCEVSVTPKTVT
Q5VYK3	1257	GQRTIAALLPCLLDKGMMSTV
Q13144	618	MDSPLDSSRYCALLLPLLKAW
Q9BY44	119	LQLYDVKTGTCLKSFIQKKMQ
Q14152	478	ERAIVDAARHCDLQVRIDHTS
Q13347	144	DNNEPYMKIPCNDSKITSAVW
O75822	207	KKITNSLTVLCSEKQKQEKQS
Q15369	11	MDGEEKTYGGCEGPDAMYVKL

Q9NPA8	40	KELLRAKLIECGWKDQLKAHC
P62495	335	NLDIMRYVLHCQGTEEEKILY
P15170	453	RPRFVKQDQVCIARLRTAGTI
P13804	53	AATRLGGEVSCLVAGTKCDKV
Q13868	68	GSVERVNKLICVKALKTRYIG
P23610	110	QERDARQRLVCPAAYGEPLQA
Q9NYY8	377	SKVVLDNIHGCPLRIMINILQ
Q7L8L6	670	KAAVPLGGFLCNVADKSGAME
P22087	99	EPHRHEGVFICRGKEDALVTK
Q9Y3I1	286	RLQLLPESFICKEKLGENVAN
P37268	6	XXXXXMEFVKCLGHPEEFYNL
Q16658	397	VFRGEHGFIGCRKVTGTLDAN
Q9C0B1	104	VSRILIGNPGCTYKYLNTRLF
Q96I24	109	QISRIQAESGCKIQIASESSG
P60520	15	FKEDHSLEHRCVESAKIRAKY
Q92616	1535	AYCAPKQLSSCLPNIVPKLTE
Q9HC38	221	AAAFGRIAFSCPQKELPDLED
Q9H4A6	280	QLLDLDPEVECLKANTNEVLW
P62993	32	GDILKVLNEECDQNWYKAELN
P48507	72	LVREFPDVLECTVSHAVEKIN
P09211	48	TWQEGSLKASCLYGQLPKFQD
Q99714	214	PLLTSLPEKVCNFLASQVPFP
P08397	114	LPPGFTIGAICKRENPHDAVV
Q00839	562	LNTLLQRAPQCLGKFIEIAAR
P00492	106	TVDFIRLKSYCNDQSTGDIKV
P19367	813	ILQQLGLNSTCDDSILVKTVC
Q13907	172	NPDPNEIKSYCYVSKEELKEL
P60842	66	SAIQQRAILPCIKGYDVIAQA
Q9H0C8	190	VISVEKTVKRCLLDTFKHTDE
P12268	140	VFEAKARHGFCGIPITDTGRM
P29218	184	MVLSNMEKLFCIPVHGIRSVG
Q9H2U2	180	VCEIGSKILSCGEVIHVKILG
O75153	753	LKDAAAFLLSCQIPGLVKDCM
P54819	92	LIEKNLETPLCKNGFLLDGFP
Q14003	370	TFEFLMRITFCPDKVEFLKSS
P12277	254	GNMKEVFTRFCTGLTQIETLF
P23919	31	TQSRKLVEALCAAGHRAELLR
P05455	232	EMKSLEEKIGCLLKFSGDLDD
Q04760	61	RVLGMTLIQKCDFPIMKFSLY
Q9H9A6	264	KLRFLPEFPSCSLLKELHVGE
O95372	56	TIRLPHVKYICPHAPRIPVTL
P23368	441	EEAYTLTEGRCLFASGSPFGP
Q9BQG0	1031	TGPVRPRHQACLLLQKTLSMR
P25205	148	RPKVVRSVHYCPATKKTIERR
P33992	482	AGITTTLNSRCSVLAAANSVF
Q13257	106	VLERWQFDIECDKTAKDDSAP
P14174	81	AQNRSYSKLLCGLLAERLRIS
P45984	163	KPSNIVVKSDCTLKILDFGLA
Q7Z6M4	219	VQQVTKILHSCPSVLREDLGQ
Q9BXJ9	721	CMIRLFNTAVCESKDLSDTVR
Q15274	96	RVAEVRGPAHCLLLGERVALN
O60502	896	RILEFYSKLGCFEIAKMEGFP
P55769	30	KKLLDLVQQSCNYKQLRKGAN
Q15233	145	RGKQLRVRFACHSASLTVRNL
Q8TAT6	188	GKFVALENISCKIKSGCEGHL

Q08J23	93	YKSHAKEILHCLKNKYFKELE
O75694	974	AFQERLNSYKCITDTLQELVN
P19338	543	FEDAKEALNSCNKREIEGRAI
P32322	262	SLLINAVEASCIRTRELQSMA
Q8NC51	11	MPGHLQEGFGCVVTNRFDQLF
Q9Y536	62	CFHRIIPGFMCQGGDFTRHNG
Q4VXU2	339	GGHSKGFGFVCFSSPEEATKA
Q99497	53	PVQCSRDVVICPDASLEDAKK
Q9UHG3	258	WAVEGGNKLVCSGLLQASKSN
Q9Y2S7	143	YYQVLIDARDCPHISQRSQTE
P30086	168	YELRAPVAGTCYQAEWDDYVP
P61758	113	TRFLLADNLYCKASVPPTDKM
P18669	153	ADLTEDQLPSCESLKDTIARA
Q9Y237	45	GNAVKVRHILCEKHGKIMEAM
Q9NYY3	26	KMCEQALGKGCGADSKKKRPP
P67775	266	VVTIFSAPNYCYRCGNQAAIM
O43447	131	GCQFFITCSKCDWLDGKHVVF
Q9Y570	312	FRGLSNLFLSCPIPKLLLLAG
P53041	404	GRSISKRGVSCQFGPDVTKAF
P30041	91	AWSKDINAYNCEEPTEKLPFP
O43395	37	EPTVVTAALNCVGKGMDKKKA
P62333	193	LARAVASQLDCNFLKVVSSSI
P49721	91	ANFTRRNLADCLRSRTPYHVN
O00232	255	KHYRAIYDTPCIQAESEKWQQ
Q9UNM6	182	YYKDALRFLGCVDIKDLPVSE
O00233	216	TRWAGKGLLGCNIIPLQRXXX
P22102	298	EFNCRFGDPECQVILPLLKSD
P31939	434	AVKYTQSNSVCYAKNGQVIGI
P11172	174	QAHGIRLHSVCTLSKMLEILE
P17812	362	VRYHEAWQKLCSAHGVLVPGG
P61106	26	IIGDMGVGKSCLLHQFTEKKF
P62820	126	NVNKLLVGNKCDLTTKKVVDY
P63000	178	VFDEAIRAVLCPPPVKKRKRK
P46060	169	GKILAAALTECHRKSSAQGKP
Q86SE5	51	IFSKYGKIVGCSVHKGYAFVQ
Q9BQ04	31	LFEQYGKVLECDIIKNYGFVH
P35250	255	FINSENVFKVCDEPHPLLVKE
P40937	238	GKVTEETVYTCTGHPLKSDIA
Q5UIP0	312	DNFALNPDILCSAKRLKLLMQ
Q06587	398	FWKVSRPLELCYAPTKDPKXX
O76021	197	SKNLSREINDCIGGTVLNISK
P62829	28	LGLPVGAVINCADNTGAKNLY
P83731	6	XXXXXMKVELCSFSGYKIYPG
P62910	91	NVKELEVLLMCNKSYCAEIAH
P62987	91	SLRQLAQKYNCDKMICRKCYA
Q96EL3	21	RPVKQVRVQFCPFEKNVESTR
Q5JTH9	317	MLTLLKDLLPCFPEGLVKSCS
Q14684	197	DQNLKFIDPFCKIAAKTKDHT
Q14690	89	SLCEGMRILGCVKEVNELELV
P15880	229	IDDCYTSARGCTATLGNFAKA
P62857	27	VLGRTGSQGQCTQVRVEFMDD
P62753	12	KLNISFPATGCQKLIEVDDER
P82933	330	VDRLGKHDVTCTVSGGGRSAQ
O94979	60	LSDPSLDMKSCATFSSSHRYH
Q99961	147	QNFIDPLQNLCEKDLKEIQHH

Q9GZT3	48	HFAQFGHVRRCILPFDKETGF
Q7KZF4	440	ETVPAFSERTCATVTIGGINI
O95219	172	LLRIASHPILCRDKIFYLFLT
P00441	7	XXXXMATKAVCVLKGDGPVQG
Q9UHB9	562	KPLVERFETFCLDPSLVTKQA
Q9BXP5	640	YPNEDEMPNRCGIIHVRGPMP
Q05519	79	DSPLPVSSRVCFVKFHDPDSA
O15400	28	ISSNIQKITQCSVEIQRTLNQ
Q9Y2Z0	88	NSTAMLRKGICEYHEKNYAAA
P54136	369	DGRKIVFVPGCSIPLTIVKSD
P26639	107	SWKTTPYQIACGISQGLADNT
P52657	98	IKVDKVKIVACDGKNTGSNTT
Q9UI30	33	LRLQATEVRICPVEFNPNFVA
Q12931	501	RAGTRNIYYLCAPNRHLAEHS
Q15631	225	RGFNKETAAACVEKXXXXXXX
Q14166	361	RPGVLLNQFPCENLLTVKDCL
Q9Y6I9	68	GETGRLFTESCSISPKLRSIA
P61088	87	HPNVDKLGRICLDILKDKWSP
Q14139	465	SRLLTFNPTYCALKELNDEER
Q9Y3C8	116	TAKMYRGGKICLTDHFKPLWA
O94874	708	THSMLHAPGRCVPQIIAFLNS
Q99536	50	AAAASPPLLRCLVLTGFGGYD
Q9NP79	155	ARWKATYIHNCLKNGETPQAG
O14980	528	VTVIKDLLGLCEQKRGKDNKA
Q9BSK1	152	KSQLNDLQKICAGGKPHECSV
P68871	94	GTFATLSELHCDKLHVDPENF

Negative dataset $\mathbb{S}^{-}$ contains 810 non-SNO sites and the corresponding peptide sequences

UniProt ID	Site	Sequence (cf. Eq.1 of the paper)
P21333	2378	VHSPSGALEECYVTEIDQDKY
Q6NXE6	201	EADLTCSGIRCVRHACLKHEQ
P34932	13	VVGIDLGFQSCYVAVARAGGI
Q29RF7	971	VKERRAHARQCLLKNISIRRE
Q14166	370	PCENLLTVKDCLASIARRAGG
O14980	199	VKSKHLKDSMCNEFSQIFQLC
O75369	2431	IEGPSKVKMDCQETPEGYKVM
P48507	46	PSTHSEELHDCIQKTLNEWSS
P17405	431	HIIGHIPPGHCLKSWSWNYYR
P29218	8	XXXMADPWQECMDYAVTLARQ
Q9UL62	808	SFNLGCKKKTCHGPPLIRTMP
P21266	91	LRYIARKHNMCGETEEEKIRV
P21333	1645	RAVPTGDASKCTVTVSIGGHG
P14780	373	SEGRGDGRLWCATTSNFDSDK
Q86VP6	237	SMSTTRTYIQCIAAISRQAGH
P62333	228	MFNYARDHQPCIIFMDEIDAI
Q08211	415	SVVIIRGATGCGKTTQVPQFI
P17812	30	ASSVGTILKSCGLHVTSIKID
P12814	774	GTLGPEEFKACLISLGYDIGN
P17844	89	SKEITVRGHNCPKPVLNFYEA
P36873	172	AAIVDEKIFCCHGGLSPDLQS

P48643	253	VEDAKIAILTCPFEPPKPKTK
P16615	70	LLVRILLLAACISFVLAWFEE
P53396	764	SEVQFGHAGACANQASETAVA
O00299	89	KIEEFLEAVLCPPRYPKLAAL
Q09161	44	LICKVGEKSACSLESNLEGLA
P14866	151	LVEFEDVLGACNAVNYAADNQ
Q06587	84	VTALRSGNKECPTCRKKLVSK
P00750	519	CQGDSGGPLVCLNDGRMTLVG
P54577	442	RGVESQGMLLCASIEGINRQV
P67775	50	TKESNVQEVRCPVTVCGDVHG
P16615	349	VETLGCTSVICSDKTGTLTTN
O00468	844	RGIVTDGRSGCTPCSCDPQGA
P21817	2237	KMVTSCCRFLCYFCRISRQNQ
P49327	1828	IRDGVVRPLKCTVFHGAQVED
Q9H0C8	367	SAADARYEAACNRLANKAVQR
P12004	62	LRSEGFDTYRCDRNLAMGVNL
P22460	564	RKVSGSRGSFCKAGGTLENAD
Q9NYY8	472	TYSSLNHVYKCQNKEQFVEVM
O00468	490	AVKNGQAACECLQACSSLYDP
P63000	157	KEIGAVKYLECSALTQRGLKT
Q9BXJ9	214	GLYREALEHLCTYEKQICDKL
P63000	18	VVGDGAVGKTCLLISYTTNAF
P35557	382	ESVSTRAAHMCSAGLAGVINR
Q9NVM4	343	LYLVAHHDDYCVWYSLQRTSP
Q5UIP0	2298	TESVYPPLVNCVAPVDIILPQ
Q8TEX9	566	GEPMRPLAEECCQLGLGLCDQ
Q8TEX9	797	LKPPGRLAELCGVLKAVLQRK
P50991	252	EKAKIGLIQFCLSAPKTDMDN
Q9NQC3	464	IKDRSGAYITCAPFNPAATES
Q9H4A6	84	REGYTSFWNDCISSGLRGCML
P78527	232	GCLKGLSSLLCNFTKSMEEDP
O94979	787	PNIMQLRDRLCRAQGEPVAGH
Q5JTH9	344	MTLSHVLVTACAMQAFHSLFH
Q8TCG1	752	EKKNKDLQITCDSLNKQIETV
P48039	146	DKLYSSKNSLCYVLLIWLLTL
Q08211	1099	KLQISHEAAACITGLRAAMEA
Q5JTH9	799	KKAYRVLEEVCASPQGPGALF
Q15149	1119	LEQGAQEESRCQRCISELKDI
P30153	447	EFFDEKLNSLCMAWLVDHVYA
P21817	3216	LEPQLNEYNACSVYTTKSPRE
P06213	268	FYLDGRCVETCPPPYYHFQDW
P21333	1353	SPFQVPVTEGCDPSRVRVHGP
P31939	363	ILSKKKNGNYCVLQMDQSYKP
P23610	190	ALQALGEAASCQLLARDYTGA
P53621	693	LQGNHQIVEMCYQRTKNFDKL
P13489	45	LDDCGLTEARCKDISSALRVN
O75694	234	GRIFLAGKDGCLYEVAYQAEA
Q99700	852	DSFIENSSSNCTSGSSKPNSP
P06213	315	VIHNNKCIPECPSGYTMNSSN
P00966	331	VYTGFWHSPECEFVRHCIAKS
Q14690	557	GFIIRVKDYGCIVKFYNNVQG
P29218	218	ADAYYEMGIHCWDVAGAGIIV
Q4G0N4	58	GQGQPRELAGCGSRADGGFRP
O60716	450	DQDNKIAIKNCDGVPALVRLL
Q06203	503	GLECFEKSGHCTACLTGKYPV

P16615	447	VGEATETALTCLVEKMNVFDT
O14983	318	PEGLPAVITTCLALGTRRMAK
P21817	3650	PLYNLPTHRACNMFLESYKAA
P00750	10	XMDAMKRGLCCVLLLCGAVFV
Q9GZT4	269	IFTVTEDEIKCATQLVWERMK
O00468	553	GALCEAETGRCVCPSECVALA
Q06587	64	MLKNTMTTKECLHRFCSDCIV
P21817	2436	YAALIDLLGRCAPEMHLIQAG
P16615	417	YDGLVELATICALCNDSALDY
P55072	209	NEVGYDDIGGCRKQLAQIKEM
Q29RF7	583	LELLISPTCSCKQADICVREI
P21817	3170	DVQVSCYRTLCSIYSLGTTKN
Q7L8L6	233	SMLDVYETKCCHQVWEMNMDQ
Q9UBF2	280	AASAIIHLPNCTARELAPAVS
O75694	1358	RFTNLCLDAVCGYLVELQSMS
P55263	182	NWMLVEKARVCYIAGFFLTVS
O00468	440	AQDGRTYDSDCWRQQAECRQQ
P21817	4877	SEDEDEPDMKCDDMMTCYLFH
Q7L8L6	345	FVMRKIGDLACANIQHLSSRS
P06213	186	KDDNEECGDICPGTAKGKTNC
Q9BQG0	338	DVIRHYGEHVCTAKLPKQFKF
Q7Z6M4	205	NDTVRLLKEKCLFTVQQVTKI
P45880	103	GTEIAIEDQICQGLKLTFDTT
Q09161	231	KPHPQEEYLDCLWAQIQKLKK
P07237	343	ELTAERITEFCHRFLEGKIKP
O00468	795	GLAGCPSACQCNPHGSYGGTC
Q9BSK1	303	TGDKPYKCSDCGRTFYFKSDL
P48200	320	VSLTLPEVVGCELTGSSNPFV
Q14181	554	LRYFVKDVLGCVCVNPGRLTK
P20618	71	FSIHTRDSPKCYKLTDKTVIG
P53618	888	LTPEKALSGYCGFMAANLYAR
P04350	127	LDVVRKEAESCDCLQGFQLTH
P31948	403	DAKLYSNRAACYTKLLEFQLA
O60502	663	MVKSFVQWLGCRSHSSAQFLI
P21817	65	QNVPPDLAICCFVLEQSLSVR
P35754	23	GKVVVFIKPTCPYCRRAQEIL
P78527	1525	LELAFAFGGLCERLVSLLLNP
P09936	201	DTLLKDAAKVCREFTEREQGE
Q9UHB9	344	ERLFESMLSECRDAIQVVREE
P23229	175	DDMDGGDWSFCDGRLRGHEKF
P23610	145	LGQPAAAAALCLELAAALRDL
O43143	114	GHAGHTSLPQCINPFTNLPHT
P06213	35	AAGHLYPGEVCPGMDIRNNLT
Q13144	247	GVEVRYDLLDCHISICSPQVA
O43175	295	GASTKEAQSRCGEEIAVQFVD
Q9BUJ2	532	EGFQRKAIVICPTDEDLKDRT
Q9BXJ9	238	ETKGELLLQLCRLEDAADVYR
P31939	325	FGDFVALSDVCDVPTAKIISR
P30044	204	NVEPDGTGLTCSLAPNIISQL
Q12879	745	LNYKAGRDEGCKLVTIGSGYI
P35579	931	EARVEEEEERCQHLQAEKKKM
Q99536	209	GGVGMAAVQLCRTVENVTVFG
P23368	120	IVYTPTVGLACSQYGHIFRRP
O00468	296	GSDGADYPGECQLLRRACARQ
Q14139	1002	FLDPIMSTLMCDPVVLPSSRV

Q9NYY8	506	ENLLDAVYSFCLMNYFPLAPF
Q7KZF4	837	LLNVEHLSAGCPHVTLQFADS
Q04760	19	GGLTDEAALSCCSDADPSTKD
Q00839	450	AGRPLFPHVLCHNCAVEFNFG
P61962	336	PDWIAICYNNCLEILRVXXXX
P78527	3912	SHFASSHALICISHWILGIGD
Q13144	95	ATGVQETFVFCCWKAAQIKEH
Q92600	252	NPRAREALRQCLPDQLKDTTF
Q7L8L6	60	CHSAKKVKNICSTFSSRRILT
P42704	361	EDVALQILLACPVSKEDGPSV
O75828	4	XXXXXXXMSSCSRVALVTGAN
P62333	170	QRVGIIPPKGCLLYGPPGTGK
O14744	278	GTNHHSEKEFCSYLQYLEYLS
P35557	213	VNDTVATMISCYYEDHQCEVG
Q92616	1446	EGALFAFEMLCTMLGKLFEPY
O00468	1553	SGVGECGDHPCLPNPCHGGAP
Q14181	375	AVINHDRPDVCILFGPFLDAK
P13489	313	LLCETLLEPGCQLESLWVKSC
Q9Y2S7	4	XXXXXXXMAACTARRALAVGS
Q14003	6	XXXXXMLSSVCVSSFRGRQGA
O43175	225	GLLNDNTFAQCKKGVRVVNCA
Q9NVM4	407	TVLKPDSVCLCVSDGSLLSVL
P46459	599	FDDAYKSQLSCVVVDDIERLL
Q8TCG1	307	SKVLELLLAFCSVTQLRHMLT
Q5VYK3	1503	ADEEKSEKEECNLWTEVWQEN
O75369	2057	IQTEDLEDGTCKVSYFPTVPG
P21817	3304	SALPAGAPPPCTAVTSDHLNS
Q16543	54	QKEKEELDRGCRECKRKVAEC
Q01518	247	PPPPPVSTISCSYESASRSSL
Q9NVM4	366	NERVRQMRPVCDCQAHLLWNR
P35250	185	DKIIEPIQSRCAVLRYTKLTD
Q5JTH9	512	GRQAHPVMRKCLQSLCDLRLS
Q06587	51	SLHSELMCPICLDMLKNTMTT
Q8TEX9	810	LKAVLQRKTACQDTDEEEEEE
P11172	86	YTALPLATVICSTNQIPMLIR
Q99832	326	EEDLKRTMMACGGSIQTSVNA
P08397	247	VLHDPETLLRCIAERAFLRHL
Q7L5N1	266	ILREAYALCHCLPVLSTDKFK
Q9UBF2	732	SCTMKFTVRDCDPNTGVPDED
P00750	430	AQESSVVRTVCLPPADLQLPD
P15170	261	NPKKDIHFMPCSGLTGANLKE
P61163	222	EIVKAIKERACYLSINPQKDE
P35557	461	LVSAVACKKACMLGQXXXXXX
Q92769	417	SIRASDKRIACDEEFSDSEDE
Q06587	72	KECLHRFCSDCIVTALRSGNK
P31948	278	VYFEKGDYNKCRELCEKAIEV
Q15149	848	QTQWSWMLQLCCCIEAHLKEN
Q8TAQ2	91	FLDFKAGGSLCHILAAAYKFK
P21333	478	SPYTVTVGQACNPSACRAVGR
P21817	3525	KKMLPIGLNMCAPTDQDLITL
P55072	695	ADLTEICQRACKLAIRESIES
Q06203	306	DQMVYTVRYRCGQQLAIEAPV
Q8TEX9	962	ERHDRVRDNICGALARLLMAS
Q92616	1076	LCASSSGDDGCAFAEQEEVDV
O75369	783	GEGDVSVGIKCDARVLSEDEE

Q9NVM4	317	EMQWRDHWMQCVYFLPQEEPV
O15382	147	PSFDKLELLECIRRLIEVDKD
P19367	834	GVVSRRAAQLCGAGMAAVVDK
Q9BY44	80	LHSFDLLKAVCLEFSPKNTVL
P78527	1742	GTPRFNNYVDCMKKFLDALEL
P07814	123	VGNSLSLADLCVWATLKGNAA
P08397	261	RAFLRHLEGGCSVPVAVHTAM
P06213	219	CWTHSHCQKVCPTICKSHGCT
O00468	705	DEDSEDGPCVCDFSCQSVPGS
Q9UHB9	247	VEEISPNIRYCAYNIGDQSAI
Q9H0C8	242	ANLGDSRAILCRYNEESQKHA
Q00987	374	KTIVNDSRESCVEENDDKITQ
P13489	85	CVLQGLQTPSCKIQKLSLQNC
Q9Y570	347	GKFQMQVLPQCGHAVHEDAPD
P13010	13	RSGNKAAVVLCMDVGFTMSNS
Q8N0X7	123	LYPEFPPKDMCEKLPEPQSFS
O75153	847	LSAAISHFLNCFLSSYPNPVA
Q96EL3	49	SEKVRSTNLNCSVIADVRHDG
P09211	170	LLIHEVLAPGCLDAFPLLSAY
O14920	618	IYTQLSKTVVCKQKALELLPK
Q9NYY3	553	TVHYYAELGQCSVFPATDAPE
P49327	630	MAAVGLSWEECKQRCPPGVVP
P29350	327	NAKTYIASQGCLEATVNDFWQ
P22314	706	VLQRPQTWADCVTWACHHWHT
O60716	618	ANNTGPHAASCFGAKKGKDEW
Q29RF7	154	NLAWVKSYNICFELEDCNEIF
P21817	64	AQNVPPDLAICCFVLEQSLSV
P07814	1480	GAPSMGAKSLCIPFKPLCELQ
Q9Y6I9	165	LDTYIKERKLCAYPRLEIYQE
Q14690	1473	PQAQKRGGRECRESGSEQERV
Q5VYK3	1806	KKLEESKQWECLTSECRVLLI
P05455	18	EKMAALEAKICHQIEYYFGDF
P50579	290	VKDATNTGIKCAGIDVRLCDV
O60443	417	CCKLQIIPTLCHLLRALSDDG
P21333	1912	IEGPSKAEISCTDNQDGTCSV
Q5VYK3	383	TLSLQFVHHICITCPEIKIKP
P62987	99	YNCDKMICRKCYARLHPRAVN
P42574	148	KITNFFRGDRCRSLTGKPKLF
P08133	552	SLETRFMTILCTRSYPHLRRV
O00468	210	SPCPSVVAPVCGSDASTYSNE
Q9UL62	922	AAQSSECPLACSSSLHCASSI
P27348	237	LWTSDSAGEECDAAEGAENXX
O00468	2016	PKAYGTGFVGCLRDVVVGRHP
P31939	575	SAADKVVIEACDELGIILAHT
P48507	114	SSTRSAVDMACSVLGVAQLDS
P37268	147	EKYQTVIADICRRMGIGMAEF
P53621	85	IKVWNYKLRRCLFTLLGHLDY
P10242	347	RPHGDSAPVSCLGEHHSTPSL
P78527	1953	AAYNCAISVICCVFNELKFYQ
P14780	468	TPQPTAPPTVCPTGPPTVHPS
Q9UHG3	20	SSLLGLWLLLCSCGCPEGAEL
O94927	12	ELAQEARELGCWAVEEMGVPV
Q9NYY8	265	LRVTQERINECDEICLSVLST
P22314	494	LKNFAMIGLGCGEGGEIIVTD
P18206	1053	KRIRTNLLQVCERIPTISTQL

Q99683	120	SEALQSLREACETVGATLETL
Q14181	346	DFEQSMVLVACGPYTTSDSIT
Q08752	176	EVKGEKPAKLCVIAECGELKE
Q9H9Q2	157	QRNQLLEVDFCIGRDIRKKDI
P14618	165	NILWLDYKNICKVVEVGSKIY
P45984	213	KENVDIWSVGCIMGELVKGCV
Q9BXJ9	711	IDSSHPWLHECMIRLFNTAVC
Q14166	563	RAFTELFQVACAKPPPLGLCD
P06213	286	QDWRCVNFSFCQDLHHKCKNS
P23229	94	YSCDITARGPCTRIEFDNDAD
Q99683	643	FQIYFCTELHCKKFFEMVNTI
Q14166	612	QILEVNFNPDCERACRYHPTF
Q9BQG0	109	LLQSFEDLPLCSILQQIQEKY
P16615	404	EVHKDDKPVNCHQYDGLVELA
Q01433	342	VYTRREPDEHCSEVELPYPDL
P54136	615	ATAFTEFYDSCYCVEKDRQTG
Q9H2U2	161	DPHEKDKSTNCFGDNDPIDVC
P21333	2102	INTEDLEDGTCRVTYCPTEPG
O00429	300	NRLLMHHIRDCLPELKTRINV
P30042	244	NKVVTTPAFMCETALHYIHDG
Q99873	285	VAYFNIEFTRCHKRTGFSTSP
Q99497	46	AGLAGKDPVQCSRDVVICPDA
Q9UHG3	22	LLGLWLLLCSCGCPEGAELRA
P78527	1399	VQVMAHLPDVCVNLMKALKMS
P14618	49	PITARNTGIICTIGPASRSVE
Q16658	260	KDELFALEQSCAQVVLQAANE
O60443	407	PDSAAALLGTCCKLQIIPTLC
Q9NX47	68	NSTARVACPQCNAEYLIVFPK
P08397	211	RVGQILHPEECMYAVGQGALG
Q9UL62	178	IPRPHQIRCNCVECVSSSEVD
P00533	510	KATGQVCHALCSPEGCWGPEP
P17812	491	RFEVNPVWKKCLEEQGLKFVG
Q9BQG0	1046	KTLSMREVRSCFEDPEWKQLM
O14979	303	SRYHQIGSGKCEIKVAQPKEV
Q5VYK3	386	LQFVHHICITCPEIKIKPLGP
P21333	483	TVGQACNPSACRAVGRGLQPK
P00750	444	ADLQLPDWTECELSGYGKHEA
Q8WX93	462	VAEGQVVVLECRVRGAPPLQV
Q14315	2154	APSIATIGSTCDLNLKIPGNW
P19838	161	ETLEARMTEACIRGYNPGLLV
P30048	108	FFYPLDFTFVCPTEIVAFSDK
P34932	310	TMNRGKFLEMCNDLLARVEPP
P43235	221	MYNPTGKAAKCRGYREIPEGN
Q92616	907	IKNPFLSLAACVMPSRLKALG
P29350	480	MENISTKGLDCDIDIQKTIQM
P17812	176	FQFKVKRENFCNIHVSLVPQP
Q13557	65	HQKLEREARICRLLKHPNIVR
Q9NYY3	347	GFTPDRLSSSCCHTVPDFHLS
P00533	595	YIDGPHCVKTCPAGVMGENNT
Q8NBU5	229	LWDGLDTDHSCQVIVMGATNR
P06213	53	NLTRLHELENCSVIEGHLQIL
P21817	936	ETLKTLLALGCHVGMADEKAE
Q15369	74	EIPSHVLSKVCMYFTYKVRYT
P28482	40	YIGEGAYGMVCSAYDNVNKVR
Q5VYK3	1364	SVLGELVPRLCELIRSGVGLG

P31327	516	LGMGGQTALNCGVELFKRGVL
Q09161	409	YMRLDTMNTTCVDRFINWFSH
P06213	495	ALKTNGDQASCENELLKFSYI
O15355	164	EELLTRYGQNCHKGPPHSKSG
O43447	122	MANSGPSTNGCQFFITCSKCD
Q8TCG1	663	QADRLIAQHRCQRTQAETEAR
O00468	494	GQAACECLQACSSLYDPVCGS
Q99798	410	AKQALAHGLKCKSQFTITPGS
Q8WX93	306	GNPTPRVRWFCEGKELHNTPD
P55072	69	LKGKKRREAVCIVLSDDTCSD
P19367	217	VNDTVGTMMTCGYDDQHCEVG
Q12879	1412	RSSLRSTASYCSRDSRGHNDV
P16615	669	ELNPSAQRDACLNARCFARVE
P78527	2093	LEMDELNRHECMAPLTALVKH
Q99615	219	NADALYVRGLCLYYEDCIEKA
P23919	163	NGAFQERALRCFHQLMKDTTL
Q92616	932	HVTLRLLKPECVLDKSWCQEE
P35579	172	MQDREDQSILCTGESGAGKTE
Q99683	835	TSKRLAGINPCTETFTGTLQY
P16615	674	AQRDACLNARCFARVEPSHKS
Q6NXE6	471	IMQARSAHRDCEDVAKAALRD
Q9P2E9	1057	QAKEESEKQLCLIEAQTMEAL
P30153	317	KEFCENLSADCRENVIMSQIL
Q99798	27	VRQYHVASVLCQRAKVAMSHF
P10242	613	PCSSTWEPASCGKMEEQMTSS
P42704	413	HSFPLQFTLHCALLANKTDLA
P78347	121	TLRKTVEDYFCFCYGKALGKS
P35579	1650	LRKLQAQMKDCMRELDDTRAS
Q6NXE6	17	SRYSSGASIGCTPTSTQAKMV
Q13907	21	KQQVQLLAEMCILIDENDNKI
P53618	8	XXXMTAAENVCYTLINVPMDS
Q86VP6	301	YPHVSTIINICLKYLTYDPNY
Q6P1X6	5	XXXXXXMWPPCGTLRTLALAR
Q9NYY8	217	MFSHPAFNQLCEHMMREAKIM
Q9NPA8	50	CGWKDQLKAHCKEVIKEKGLE
O00468	270	ARSADGLTASCLCPATCRGAP
Q9UBF2	88	QSNDQTLRRMCYLTIKEMATI
Q4G0N4	12	TCYRGFLLGSCCRVAGGRAAA
P63244	153	QDESHSEWVSCVRFSPNSSNP
P08133	59	SRSNRQRQEVCQSYKSLYGKD
P68104	111	NMITGTSQADCAVLIVAAGVG
Q15149	4574	DVGAYSKYLTCPKTKLKISYK
P14174	57	LMAFGGSSEPCALCSLHSIGK
Q9UQM7	64	HQKLEREARICRLLKHPNIVR
Q5VYK3	1638	NVKYKIVAISCAADILKATKE
Q9UBF2	723	DPTAVAGSFSCTMKFTVRDCD
O00220	262	LLLVAVLIVCCCIGSGCGGDP
P11172	255	RLMQKKETNLCLSADVSLARE
Q00987	464	KTGHLMACFTCAKKLKKRNKP
P50991	414	SIHDALCVIRCLVKKRALIAG
P33992	397	KSQLLKFVEKCSPIGVYTSGK
Q13868	240	DREVISRLRNCIISLVTQRMM
Q96EK6	45	PGEGLVLRPLCTADLNRGFFK
P30153	512	LFCINVLSEVCGQDITTKHML
Q5UIP0	1155	LEKSSLSNNECGSLDKTSPEM

Q9NPH0	208	SEVLYPNYQSCWSLRQRTRGR
P29474	908	RRYEEWKWFRCPTLLEVLEQF
P21817	1150	RPWQPGDVVGCMIDLTENTII
P53396	519	VQGMLDFDYVCSRDEPSVAAM
O95573	166	QKPKTNIAIFCETRAEWMIAA
P29474	368	MSTEIGTRNLCDPHRYNILED
P11926	114	PPERIIYANPCKQVSQIKYAA
Q14003	184	WGIDETDVEACCWMTYRQHRD
Q5JTH9	901	FGSNQEEALQCYLVLIYPGLV
Q99873	93	LDVGSGTGILCMFAAKAGARK
Q99798	205	TPNGGGLGGICIGVGGADAVD
P27635	8	XXXMGRRPARCYRYCKNKPYP
O00220	274	IGSGCGGDPKCMDRVCFWRLG
O75369	1087	VEGPCEAKIECSDNGDGTCSV
P13489	323	CQLESLWVKSCSFTAACCSHF
P31327	761	KNVVSGKTSACFEPSLDYMVT
P34932	376	LNADEAVTRGCALQCAILSPA
Q9C0B1	308	LDDLNATHQHCVLAGSQPRFS
P23368	428	ALSNPTAQAECTAEEAYTLTE
P00966	19	LAYSGGLDTSCILVWLKEQGY
Q4VXU2	132	FGNILSCKVACDEHGSRGFGF
Q9C0B1	456	QNLRREWHARCQSRIARTLPA
O75369	450	SPFVVQVGEACNPNACRASGR
P23396	134	RFIMESGAKGCEVVVSGKLRG
Q13347	76	HVLTGSADNSCRLWDCETGKQ
Q06587	332	GASDTGGPDGCGGEGGGAGGG
Q14315	2369	IAFEDRKDGSCGVSYVVQEPG
Q9P2E9	933	QSSEAEVRSKCEELSGLHGQL
P21817	1685	LGNNRVAHALCSHVDQAQLLH
Q9H2U2	274	ALEVIKSTHQCWKALLMKKCN
P05062	158	WRAVLRIADQCPSSLAIQENA
P63000	81	SYPQTDVFLICFSLVSPASFE
O94874	641	NEKSIEDFISCLDSAAEACDI
P06213	280	PPYYHFQDWRCVNFSFCQDLH
P78527	3001	KDFWELASLDCYNHLAEWKSL
O00468	948	GSDGVTYGNECQLKTIACRQG
Q9UQM7	289	SCMHRQETVDCLKKFNARRKL
P26639	172	GEAMERVYGGCLCYGPPIENG
O75694	917	ISNQVDLSNVCAQYRQVRFYE
P62826	85	LRDGYYIQAQCAIIMFDVTSR
Q04206	105	CRDGFYEAELCPDRCIHSFQN
P21333	210	ALVDSCAPGLCPDWDSWDASK
P15170	464	IARLRTAGTICLETFKDFPQM
P26196	361	AKKISQLGYSCFYIHAKMRQE
Q8TCG1	413	NLDEALTRKKCERIAKAIEVL
P55072	174	KVVETDPSPYCIVAPDTVIHC
P13489	95	CKIQKLSLQNCCLTGAGCGVL
P10599	35	VDFSATWCGPCKMIKPFFHSL
Q9UBF2	516	LPSILVLLQRCMMDTDDEVRD
Q9UHG3	242	NAFVGAVSLSCSDSGLWAVEG
P61962	228	DPQHHPLLRLCWNKQDPNYLA
P15170	419	GYNAVLHIHTCIEEVEITALI
Q14315	1180	VGEAATFTVDCSEAGEAELTI
P14618	358	VANAVLDGADCIMLSGETAKG
O75694	667	IVYSGKHNGICIYFSRIMGNI

P42704	394	NTPVEKLTDYCKKLKEVQMHS
P00533	571	ECLPQAMNITCTGRGPDNCIQ
Q6NXE6	6	XXXXXMSERCCSRYSSGASIG
P53618	635	PLMNDIFNKECRQSLSHMLSA
Q12879	1239	TMRSPFKCDACLRMGNLYDID
Q9Y2Z0	62	AYCHILLGNYCVAVADAKKSL
Q5UIP0	596	IQLIFNNFLECGVSDERFFLS
P12277	141	VRTGRSIRGFCLPPHCSRGER
P00533	19	AALLALLAALCPASRALEEKK
Q9H9A6	54	SGRNLSEVPQCVWRINVDIPE
Q99798	226	VMAGIPWELKCPKVIGVKLTG
Q14690	705	LSQSEGRVLLCRKPALVSTVE
P48200	604	NAVKQGDLVTCGILSGNKNFE
P16615	377	FILDRVEGDTCSLNEFTITGS
Q9BUJ2	421	GTVGPKSKAECEILMMVGLPA
Q92616	2179	QAAAIILNIYCSRSKADYTSH
P10242	542	SPTDKSGNFFCSHHWEGDSLN
P78347	123	RKTVEDYFCFCYGKALGKSTV
Q7KZF4	736	GSYAPRRGEFCIAKFVDGEWY
P53041	240	TLKETEKITVCGDTHGQFYDL
O75694	1276	FIVQFLEQQVCTLNWDVGFVI
Q9BUJ2	391	FNFGQRAEPYCSVLPGFTFIQ
P13489	75	SNELGDVGVHCVLQGLQTPSC
P30048	127	DKANEFHDVNCEVVAVSVDSH
P08133	248	FEKLMLAVVKCIRSTPEYFAE
O00468	1519	RTFVGAGLRGCIRLLDVNNQR
Q92945	296	IGDPYKVQQACEMVMDILRER
Q16658	19	AVQIQFGLINCGNKYLTAEAF
P23368	274	RFLRKYREKYCTFNDDIQGTA
O60443	331	DELLMVLEPVCDDLVSGLSPT
P11172	365	QEVGLPLHRGCLLIAEMSSTG
Q9NX47	97	DLADRLISKACPFAAAGIMVG
O95573	466	SATTQRFMNICFCCPVGQGYG
Q13347	81	SADNSCRLWDCETGKQLALLK
P21817	3278	HVIEITLPMLCSYLPRWWERG
P55072	415	GHVGADLAALCSEAALQAIRK
P60981	163	GGSLIVAFEGCPVXXXXXXXX
Q8WX93	1152	GDLTVQEGKLCRMDCKVSGLP
P21817	1781	LRPPHHFSPPCFVAALPAAGA
P00533	579	ITCTGRGPDNCIQCAHYIDGP
O75694	1326	RMKKPLHLLDCIHVLLIRYVE
O00170	78	FKLPVWETIVCTMREGEIAQF
P22314	444	EDKEVLTEDKCLQRQNRYDGQ
Q92616	414	VHAVSVLALWCNRFTMEVPKK
P78527	1919	NELTKTLIKLCYDAFTENMAG
P49915	523	PIKTVGVQGDCRSYSYVCGIS
P49327	135	ETLVGYSMVGCQRAMMANRLS
P21817	5028	RCWDFFPAGDCFRKQYEDQLS
Q9C0B1	392	FWFQGNRYRKCTDWWCQPMAQ
O43447	128	STNGCQFFITCSKCDWLDGKH
P50579	223	LNAGLAFPTGCSLNNCAAHYT
P21817	3733	MAYADIMAKSCHLEEGGENGE
O43175	48	KEELIAELQDCEGLIVRSATK
O95372	135	GALSLYTALTCPHPLAGIVAL
P49327	1558	CSSLRHAQPTCPGAQLCTVYY

Q00610	292	IHLYDLETGTCIYMNRISGET
P78527	1455	RSRLAAVVSACKQLHRAGLLH
Q9BXP5	441	NISRAEIISLCKRYPGFMRVA
Q14315	1066	GVLPPDPSKVCAYGPGLKGGL
Q8WX93	344	AFEDDTGRYTCLATNPSGSDT
Q5UIP0	718	WSELYRAFARCAALVATAEEN
Q09666	5382	GDLAVSGDIKCPKVSVGAPDL
Q9C0B1	346	QRCQLALQNVCDDVDNDDVSL
P17987	125	TSVISGYRLACKEAVRYINEN
Q9NQC3	597	QESLYPAAQLCPSFEESEATP
Q14139	286	LLGRIKDLELCQILLYAYLDI
P49411	222	ETPVIVGSALCALEGRDPELG
P17405	385	LRLISLNMNFCSRENFWLLIN
Q8TCG1	445	HIAKILTTVKCTTLIEQQFTY
P49327	1548	RGDLSSIRWVCSSLRHAQPTC
P37268	43	QDSLSSSLKTCYKYLNQTSRS
P49327	779	AVLKRGLKPSCTIIPLMKKDH
Q9HC38	171	SDLQKSLNYWCNLLGMKIYEK
P15121	187	KYKPAVNQIECHPYLTQEKLI
Q14315	618	IEGPSQAKIECDDKGDGSCDV
P60842	134	GDYMGASCHACIGGTNVRAEV
P27635	105	HVIRINKMLSCAGADRLQTGM
Q99798	332	FKDHLVPDPGCHYDQLIEINL
Q9UHB9	586	HFPPGFQPIPCKPLFFDLALN
P30101	57	LMLVEFFAPWCGHCKRLAPEY
P78527	3403	EEEAQPPSWSCGPAAGVIDAY
P63244	207	TVTVSPDGSLCASGGKDGQAM
P43243	803	DYVIPKTGFYCKLCSLFYTNE
P13489	266	SRLRTLWIWECGITAKGCGDL
P23975	44	LVVKERNGVQCLLAPRDGDAQ
P18206	422	LAEARKIAELCDDPKERDDIL
Q14690	505	KKYHIGDEVKCRVLLCDPEAK
P78527	1176	LDLVKWLLAHCGRPQTECRHK
P00750	492	LNRTVTDNMLCAGDTRSGGPQ
Q7Z6M4	142	ILLGLNPEPVCVVLKKSPQLL
P68104	363	ISAGYAPVLDCHTAHIACKFA
Q7L8L6	689	MEMAGLCPAACMQTPRMKLAV
P22460	26	VRGGDEARAGCGQATGGELQC
P07814	359	GCMRDPTLYRCKIQPHPRTGN
Q13907	80	RSDAKITFPGCFTNTCCSHPL
P23975	131	REGAATVWKICPFFKGVGYAV
O00299	178	LDGNELTLADCNLLPKLHIVQ
Q9UL62	307	HQKEFVAQPNCQQLLATLWYD
P00750	78	YCWCNSGRAQCHSVPVKSCSE
O75694	1201	EFADPFKLAECKLAIIHCAGY
P40227	499	AAEVGVWDNYCVKKQLLHSCT
O95801	174	LKHFAEAVNWCDEGLQIDAKE
Q29RF7	956	PLEYMAIFALCAKDPVKERRA
P22460	500	TVGGKIVGSLCAIAGVLTIAL
P48039	100	NNGWNLGYLHCQVSGFLMGLS
P29350	171	PLRVTHIKVMCEGGRYTVGGL
Q99700	152	RPAPGCPRPACEPVYGPLTMS
P60981	46	VIFCLSADKKCIIVEEGKEIL
Q16665	337	TKNSQPQCIVCVNYVVSGIIQ
P20073	327	GHFERLLVSMCQGNRDENQSI

P49327	1403	KSFYGSTLFLCRRPTPQDSPI
Q14315	2088	IEGPSKVDINCEDMEDGTCKV
P21817	1673	SHTLRLYRAVCALGNNRVAHA
P48444	380	EESFIPLTINCWPSESGNGCD
Q8TAT6	556	SEQWATIEQLCSTVGGQLPGL
Q13557	428	HVHLVGDDAACIAYIRLTQYM
Q99615	116	HLSLGNAMAACRSFQRALELD
P22102	62	ISDHTALAQFCKEKKIEFVVV
Q9BSK1	216	RTHTGEKPHECSECGKAFSRK
P62879	166	QIITSSGDTTCALWDIETGQQ
O60502	631	VMGMFTRLSNCANRTILYDMY
P16615	420	LVELATICALCNDSALDYNEA
Q5UIP0	2068	MLTAEMDNFVCDTVEMSTEEG
P21817	4959	EQVKEDMETKCFICGIGSDYF
Q9C0B1	77	EAFLTLHKHGCLFRDLVRIQG
P53618	189	DFLVNEKDASCKRNAFMMLIH
P36873	39	QLQENEIRGLCLKSREIFLSQ
P11926	217	TFVQAISDARCVFDMGAEVGF
O75369	178	GKALGALVDSCAPGLCPDWES
P21333	2582	VGQKSSFTVDCSKAGNNMLLV
P30101	409	IEFYAPWCGHCKNLEPKYKEL
Q13907	86	TFPGCFTNTCCSHPLSNPAEL
P50579	380	GKGVVHDDMECSHYMKNFDVG
Q9NP79	276	PEDFARAQKYCKYAGSALQYE
P26641	166	RVTLADITVVCTLLWLYKQVL
P00533	248	AGCTGPRESDCLVCRKFRDEA
O15519	298	GISQILGQFACMPEHRDYDSF
P55060	853	AVGITKLLTECPPMMDTEYTK
P78527	4061	LAGANPAVITCDELLLGHEKA
Q8TCG1	358	LSQPLDGSENCSVLALELFKE
O00468	812	GGTCDPATGQCSCRPGVGGLR
P55072	77	AVCIVLSDDTCSDEKIRMNRV
P22102	466	PLAKATSRSGCKVDLGGFAGL
Q15149	965	VEVTVHKGDECQLVGPAQPSH
O95219	318	LFYAEALRAVCRKHELMQYDL
O43390	226	KEAAQEAVKLCDSYEIRPGKH
Q9NYY3	96	KVLGKGGFAKCYEMTDLTNNK
O00468	488	TCAVKNGQAACECLQACSSLY
Q7Z6M4	57	SNGGVIEELSCVRSNNYVQEP
Q06203	496	MIQENGNGLECFEKSGHCTAC
P68366	200	TTHTTLEHSDCAFMVDNEAIY
P35579	468	IFDLNSFEQLCINYTNEKLQQ
P29350	382	RAYGPYSVTNCGEHDTTEYKL
P06213	331	MNSSNLLCTPCLGPCPKVCHL
Q5UIP0	613	FFLSLESLVGCVLSGPTSPLA
P21817	3240	LGLPNSVEEMCPDIPVLERLM
Q06203	280	SRSEGNPVAFCIFEYVYFARP
P22314	413	GLAAQEVMKACSGKFMPIMQW
O15382	22	RKLLSVPWLLCGPRRYASSSF
Q5UIP0	1630	EKQDESNTVICQDSTVTSDLL
Q99714	5	XXXXXXMAAACRSVKGLVAVI
P17812	299	ADRYDRLLETCSIALVGKYTK
Q9GZT4	128	IQAYGASIVYCEPSDESRENV
O60502	215	YLGEPETFLFCPTEYCGTFCY
P49915	554	IFLARLIPRMCHNVNRVVYIF

P29474	618	SYKIRFNSISCSDPLVSSWRR
Q8N0X7	166	PASLSLPSQSCPAEAPPAYTP
P04350	211	CIDNEALYDICFRTLKLTTPT
P12814	370	MVSDINNAWGCLEQVEKGYEE
P23975	339	FASYNKFDNNCYRDALLTSSI
O15523	221	IIKGKRDLMACAQTGSGKTAA
Q99439	215	TISLQMGTNKCASQVGMTAPG
Q5JTH9	326	PCFPEGLVKSCSETLLRVMTL
Q5VYK3	540	VHGEAQRVLRCLPGRNRKEST
Q00839	295	DTVVCLDTYNCDLHFKISRDR
O95801	160	HLKAIIRGALCHLELKHFAEA
Q08211	612	EDDDANCNLICGDEYGPETRL
Q5UIP0	1077	HQKEVLKTKRCDIPAMYNNLD
Q14684	61	LLKIWKGLFYCMWVQDEPLLQ
O75390	101	IRFRGFSIPECQKLLPKAKGG
Q9UBF2	446	LAHLCEFIEDCEHTVLATKIL
Q15417	273	LGRQVYDPKYCAAPTEPVIHN
P21817	2305	LEKVVSYLAGCGLQSCPMLVA
P43235	269	YSKGVYYDESCNSDNLNHAVL
Q14003	69	RGPGDRRAEPCPGLPAAAMGR
Q9BY44	132	SFIQKKMQNWCPSWSEDETLC
P17987	385	IILRGANDFMCDEMERSLHDA
P32322	120	AFRPAPRVIRCMTNTPVVVRE
P23368	481	FPGVALAVILCNTRHISDSVF
P40227	343	LNSFDDLSPDCLGHAGLVYEY
Q5JTH9	865	ITALIPEVILCTKEVSVGARK
O60716	429	HPKKEVHLGACGALKNISFGR
O14980	920	AAQSFYQTYFCDILQHIFSVV
Q16658	456	DTPVDFFFEFCDYNKVAIKVG
O15382	249	LVQQEALKRGCEQVLWLYGPD
O75369	416	VEDKGNQVYRCVYKPMQPGPH
P55263	140	YEQNEQPTGTCAACITGDNRS
P30153	504	NYLHRMTTLFCINVLSEVCGQ
Q92600	167	FLLTTEIIPLCLRIMESGSEL
P09382	131	MAADGDFKIKCVAFDXXXXXX
O94979	458	AVQSQGFINYCQKKIDASQTE
P41091	269	IRSFDVNKPGCEVDDLKGGVA
Q9BSK1	191	RGEKPHGCGECGKTFMRKIQL
Q9BQ04	165	GMGDQSGCYRCGKEGHWSKEC
P30153	310	AAASHKVKEFCENLSADCREN
Q08752	296	MSNWQGAIDSCLEALELDPSN
Q16665	139	GHSVFDFTHPCDHEEMREMLT
P13489	305	ELGDEGARLLCETLLEPGCQL
O14983	268	EQLSKVISLICVAVWLINIGH
P53618	143	LEPLMPAIRACLEHRHSYVRR
Q9NVM4	294	IEMDPEGKIKCTMAPFWAHSD
P78527	1791	SFRRIARRGSCVTQVGLLESV
Q00839	648	GNFTLPEVAECFDEITYVELQ
O94927	192	PVVLRDVRTACTLRAQFLQNL
P13489	427	QLVESVRQPGCLLEQLVLYDI
Q9NPH0	23	PVGVLTSLAYCLHQRRVALAE
Q9NYY8	44	LVSTSRTMRLCCLGLCKPKIV
P50995	501	GDYRKILLKICGGNDXXXXXX
Q7L8L6	425	AASLPPRVAHCRSKDVAKILW
Q5UIP0	1865	PNENFKTVGPCLGDSKNVSQE

O43143	307	AGKFQIYFDNCPLLTIPGRTH
P98170	453	RLQEEKLCKICMDRNIAIVFV
Q9UBF2	230	KSGLKSQFAYCMLIRIASRLL
P78527	931	ASDRQTKVAACELLHSMVMFM
Q9UL62	918	EVQGAAQSSECPLACSSSLHC
Q3LXA3	155	VLKKAGRRGLCGTVLIHKVAG
Q4VXU2	497	QTTGPSGVGCCTPGRPLLPCK
Q9H2U2	283	QCWKALLMKKCNGGAINCTNV
Q9NYY8	269	QERINECDEICLSVLSTVLEA
O14744	196	WMWWHNFRTLCDYSKRIAVAL
P78527	10	XMAGSGAGVRCSLLRLQETLS
Q9Y3I1	252	LCEGSSATLTCVPLGNLIVVN
O60443	489	LLLCITLNGLCALGREHSXXX
P40937	169	SKIIPALQSRCTRFRFGPLTP
P62280	60	AIEGTYIDKKCPFTGNVSIRG
Q29RF7	430	MGLAQLYKKYCLHGEAGKEAA
P48200	720	PWDLKSTYIRCPSFFDKLTKE
Q14003	575	RPPQPGSPNYCKPDPPPPPPP
O75153	602	GFPRAHRHKLCCLRQELVDAF
P21817	4658	SLLHTLVAFLCIIGYNCLKVP
P19367	581	GEELFDHIVSCISDFLDYMGI
P22314	481	YFLVGAGAIGCELLKNFAMIG
Q9UQM7	373	NGDFESYTKMCDPGMTAFEPE
P33992	207	CNTDQAGRPKCPLDPYFIMPD
P00750	71	LRSNRVEYCWCNSGRAQCHSV
Q99683	391	SEGQVASDMYCLVGRIYKDMF
Q6NXE6	118	QEVSAYLTRFCDQCKQDKACR
Q5UIP0	2450	QLFEMHEKLSCMANSVIKNLQ
Q14181	31	EALIEKLVELCVQYGQNEEGM
O95573	342	HVLELSAELVCLSHGCRIGYS
Q8TAT6	208	LPWPNGICTKCQPSAITLNRQ
P49327	2318	SYGACVAFEMCSQLQAQQSPA
P31749	77	RPRPNTFIIRCLQWTTVIERT
Q9Y696	189	LDGNEMTLADCNLLPKLHIVK
Q86VP6	802	HKQSYYSIAKCVAALTRACPK
P13639	751	MEPIYLVEIQCPEQVVGGIYG
P16615	595	KYETNLTFVGCVGMLDPPRIE
Q06203	17	GIREECGVFGCIASGEWPTQL
O00233	59	GIGMNEPLVDCEGYPRSDVDL
Q16555	334	GDLQVTGSAHCTFNTAQKAVG
O94874	649	ISCLDSAAEACDIMVKRGDKK
O94927	609	PGQAALSEELCQGLSLPQWRL
P27635	49	KKAKVDEFPLCGHMVSDEYEQ
Q9HC38	197	RALLGYADNQCKLELQGVKGG
P52272	709	FESPEVAERACRMMNGMKLSG
P26599	23	KRGSDELFSTCVTNGPFIMSS
P43235	210	AYPYVGQEESCMYNPTGKAAK
Q12879	1217	YRQNSTHCRSCLSNMPTYSGH
P49411	387	VMFSLTWDMACRIILPPEKEL
P48200	375	IVDRTTIANMCPEYGAILSFF
Q14684	155	FLDVLMKEVLCPESQSPNGVR
Q14003	646	LPPLPAPGEPCPLAQEEVIEI
O75369	1868	IEGPSKAEISCIDNKDGTCTV
O15355	351	LIVANAGDSRCVVSEAGKALD
P13804	159	YAGNALCTVKCDEKVKVFSVR

Q9NVM4	171	HAHRHLVEENCEAVPHRATVY
P23229	535	KTACGAPSGICLQVKSCFEYT
O14744	449	SFADNELSPECLDGAQHFLKD
O60502	618	RSRAAKFEEMCGLVMGMFTRL
P78527	392	DFMYVELIQRCKQMFLTQTDT
O00468	285	TCRGAPEGTVCGSDGADYPGE
O95372	213	KTYPGVMHSSCPQEMAAVKEF
O14744	536	RDPMIDNNRYCTLEFPVEVNT
P19367	224	MMTCGYDDQHCEVGLIIGTGT
O14983	377	FIIDKVDGDICLLNEFSITGS
Q13347	57	TYMGHTGAVWCVDADWDTKHV
Q13144	414	AGAQIHQSLLCDNAEVKERVT
Q9BQG0	890	THHLCRARRYCHDLGERAGAL
P26639	174	AMERVYGGCLCYGPPIENGFY
O00429	446	HEEMQRIIQHCSNYSTQELLR
P35579	1834	DNETKERQAACKQVRRTEKKL
P62736	2	XXXXXXXXXMCEEEDSTALVC
O00170	121	GKDPLEGQRHCCGVAQMREHS
O95433	301	FIDKNGETELCMEGRGIPAPE
O00468	349	RPEMLLRPESCPARQAPVCGD
P45983	426	LEAAAGPLGCCRXXXXXXXXX
O00468	1576	NLEAGRFHCQCPPGRVGPTCA
P00750	179	IRLGLGNHNYCRNPDRDSKPW
Q4VXU2	128	TFSTFGNILSCKVACDEHGSR
P21817	4646	STGYMEPALRCLSLLHTLVAF
O00468	877	SCKPGVAGPKCGQCPDGRALG
Q16665	780	TIILIPSDLACRLLGQSMDES
Q9NPH0	416	TLSPEKYHALCSQTQVMEVGN
Q9BXJ9	465	ANLIKEAEEMCSKFTREGTSA
O75694	102	ELVEQFGHMQCNCMMGVFPPI
P17405	227	TDPDCADPLCCRRGSGLPPAS
P23975	176	SFTLNLPWTDCGHTWNSPNCT
O14980	369	EVEETEIFKICLEYWNHLAAE
P07814	660	KHEELMLGDPCLKDLKKGDII
P61106	215	EPQPQREGCGCXXXXXXXXXX
Q14181	19	AEELQIFGLDCEEALIEKLVE
Q08752	181	KPAKLCVIAECGELKEGDDGG
P49327	2202	SKADEASELACPTPKEDGLAQ
Q7Z6M4	183	EGKLKRVLYCCPEIFTMRQQD
P22102	540	EPLFFLDYFSCGKLDLSVTEA
P55060	630	EAICLSIRITCKANPAAVVNF
O15355	495	APDTSGDGTGCDNMTCIIICF
Q5UIP0	731	LVATAEENLCCEELSSKIMSS
O00231	257	AITSLKYMLLCKIMLNTPEDV
O00468	317	ENVFKKFDGPCDPCQGALPDP
O00468	414	VCLSRRGRPRCSCDRVTCDGA
Q6NXE6	127	FCDQCKQDKACRFLAAQKGAY
P21817	603	GRNHKVLDVLCSLCVCNGVAV
P21980	211	PKFLKNAGRDCSRRSSPVYVG
P07355	335	GDYQKALLYLCGGDDXXXXXX
O00468	197	AEGPGRASCVCKKSPCPSVVA
P53041	343	EVFEWLPLAQCINGKVLIMHG
Q14181	392	LDAKHEQVENCLLTSPFEDIF
O43390	99	SHVQNKSAFLCGVMKTYRQRE
P00750	509	GGPQANLHDACQGDSGGPLVC

P40926	275	AMNGKEGVVECSFVKSQETEC
P00533	260	VCRKFRDEATCKDTCPPLMLY
Q14139	490	VHMRGLDKETCLIPAVQEPKF
P17405	584	YHKGHPPSEPCGTPCRLATLC
Q8TAT6	355	GDFQNKHPNMCRLSPDGHFGS
O75369	1617	RATQTGDASKCLATGPGIAST
P53396	893	PKYSCQFIEMCLMVTADHGPA
Q9NYY8	366	NHRSLILLDECSKVVLDNIHG
O00232	244	DQHEGSYLSICKHYRAIYDTP
O43707	495	YYDSHNVNTRCQKICDQWDAL
Q16543	183	KYLSDNVHLVCEETANYLVIW
Q9BUJ2	331	DKFAENDVIGCFADFECGNDV
P00533	236	KSPSDCCHNQCAAGCTGPRES
P30153	329	ENVIMSQILPCIKELVSDANQ
O00468	172	TPVPPTPPDACRGMLCGFGAV
P35557	434	HASVRRLTPSCEITFIESEEG
P21817	2656	NHYERCWKYYCLPTGWANFGV
P21817	606	HKVLDVLCSLCVCNGVAVRSN
P53621	140	VLTGHNHYVMCAQFHPTEDLV
P49327	1564	AQPTCPGAQLCTVYYASLNFR
P13639	131	VTDGALVVVDCVSGVCVQTET
Q06587	48	SPRSLHSELMCPICLDMLKNT
Q9BQ04	89	KLHVGNISPTCTNQELRAKFE
P49368	40	AKTIADIIRTCLGPKSMMKML
Q9BXP5	628	IVHSLDYYNTCEYPNEDEMPN
P30556	76	FLLNLALADLCFLLTLPLWAV
P42704	722	GGYAALINLCCRHDKVEDALN
Q9Y3C8	165	QKGVIQHKEKCNQXXXXXXXX
O00220	229	KDCTPWSDIECVHKESGNGHN
Q14139	710	SSVFHRKRVFCNFQYAPQLAE
P45984	116	LVMELMDANLCQVIHMELDHE
P31327	225	GNPTKVVAVDCGIKNNVIRLL
Q9NYY8	645	AVLCVSRSAYCLGSSHPRGFL
P53396	728	EIGGTEEYKICRGIKEGRLTK
O75369	991	ILSPSRKVVPCLVTPVTGREN
P78527	1032	DSTLRDFCGRCIREFLKWSIK
P07814	1076	AEIKKLGVENCYFPMFVSQSA
Q99439	240	IYDTKLGTDKCDNSSMSLQMG
Q99683	622	VSISKFEERCCFLYVLHNSDD
O14920	99	NDLPLLAMEYCQGGDLRKYLN
Q15233	208	KPAARKALDRCSEGSFLLTTF
P98170	474	PCGHLVTCKQCAEAVDKCPMC
P07814	1046	EMIEYHDISGCYILRPWAYAI
O75153	333	TAPQAEHAMDCVRAEDAYTSR
P48200	512	GSVVIAAVISCTNNCNPSVML
Q00610	909	YYDSRVVGKYCEKRDPHLACV
Q09161	503	NSLPGHSVALCLAVAFKSKAT
P05062	202	IPDGDHDLEHCQYVTEKVLAA
P37268	374	STIRTQNLPNCQLISRSHYSP
P13010	157	LDIIIHSLKKCDISLQFFLPF
P21817	2555	EMALALNRYLCLAVLPLITKC
O00468	664	TQIEEARAGPCEQAECGSGGS
P53618	235	LVIVELIYKVCHANPSERARF
P06213	911	SRKHFALERGCRLRGLSPGNY
Q6P1X6	130	ADHGPPRLSYCGGGEALAVPF

O75153	1196	KTKESSEYLKCLTQQAVALQR
P11926	202	VGVSFHVGSGCTDPETFVQAI
P21980	505	ITNNTAEEYVCRLLLCARTVS
O00468	923	VEESGSAHCVCPMLTCPEANA
Q14003	185	GIDETDVEACCWMTYRQHRDA
Q00610	1528	NNRWKQSVELCKKDSLYKDAM
P07237	8	XXXMLRRALLCLAVAALVRAD
Q86VP6	286	IQAFESFVRRCPKEVYPHVST
O43143	644	FKQNHESVQWCYDNFINYRSL
P62829	125	SAITGPVAKECADLWPRIASN
Q86VP6	71	NGEVQNLAVKCLGPLVSKVKE
Q9GZT4	2	XXXXXXXXXMCAQYCISFADV
Q06587	279	HPLLVEKGEYCQTRYVKTTGN
P47712	220	KALYESGILDCATYVAGLSGS
O00468	1843	VPREAAYVCLCPGGFSGPHCE
O14983	636	GDNKGTAIAICRRIGIFGENE
P06213	235	SHGCTAEGLCCHSECLGNCSQ
P78527	1954	AYNCAISVICCVFNELKFYQG
Q5VYK3	1390	ASVIVSLTTQCPQDLTPYSGK
