# Supplementary material for: iSNO-PseAAC: Predict Cysteine S-Nitrosylation Sites in Proteins by Incorporating Position Specific Amino Acid Propensity into Pseudo Amino Acid Composition
Source: PLoS One. 2013 Feb 7;8(2):e55844. doi: 10.1371/journal.pone.0055844 (PMC3567014; doi:10.1371/journal.pone.0055844)
Supplement: Supporting Information S2 — Predicted results by iSNO-PseAAC on an independent dataset of 461 proteins, which have been verified by experiments as nitrosylated proteins but none of which occurs in the 438 proteins used to train the current predictor. The overall success rate was (PDF) [file pone.0055844.s002.pdf]

Online Supporting Information S2. Predicted results by iSNO-PseAAC on an independent dataset of 461 proteins, which have been verified by experiments as nitrosylated proteins but none of which occurs in the 438 proteins used to train the current predictor. The overall success rate was $416/461 = 92.24\%$.

UniProt ID of the nitrosylated proteins	The SNO site was detected?
A2AUS0	Yes
A8MRZ7	Yes
B1AVT9	Yes
B1AXT2	Yes
B4DX73	Yes
B9DGD1	Yes
O03042	Yes
O08759	Yes
O14556	Yes
O14645	Yes
O14715	Yes
O15027	Yes
O23255	Yes
O43374	Yes
O43719	Yes
O43933	Yes
O48646	Yes
O49485	Yes
O50008	Yes
O60309	Yes
O60437	Yes
O65396	Yes
O75037	Yes
O75179	Yes
O75369	Yes
O75390	Yes
O75952	Yes
O75969	Yes
O82660	Yes
O88307	Yes
O94769	Yes
O95271	Yes
O95861	Yes
O95886	Yes
P00338	Yes
P00367	Yes
P00505	Yes

P00558	Yes
P00568	Yes
P00747	Yes
P00918	Yes
P02768	Yes
P02787	Yes
P02788	Yes
P04075	Yes
P04264	Yes
P04406	Yes
P04637	Yes
P05091	Yes
P06396	Yes
P06733	Yes
P06744	Yes
P06748	Yes
P07195	Yes
P07205	Yes
P07237	Yes
P07288	Yes
P07310	Yes
P07724	Yes
P07864	Yes
P07900	Yes
P07947	Yes
P07954	Yes
P08107	Yes
P08238	Yes
P08670	Yes
P08752	Yes
P09542	Yes
P09622	Yes
P09972	Yes
P10323	Yes
P10515	Yes
P10795	Yes
P10796	Yes
P10797	Yes
P10896	Yes
P11021	Yes
P11142	Yes
P11169	Yes
P11177	Yes
P12110	Yes

P12270	Yes
P12532	Yes
P12883	Yes
P13639	Yes
P13861	Yes
P14136	Yes
P14152	Yes
P14618	Yes
P14625	Yes
P15104	Yes
P15259	Yes
P15313	Yes
P15924	Yes
P16152	Yes
P17066	Yes
P17174	Yes
P17612	Yes
P17661	Yes
P17745	Yes
P17987	Yes
P18669	Yes
P19171	Yes
P19366	Yes
P19367	Yes
P21266	Yes
P21333	Yes
P21796	Yes
P22953	Yes
P22954	Yes
P23368	Yes
P23458	Yes
P24539	Yes
P24704	Yes
P24752	Yes
P25696	Yes
P25697	Yes
P25705	Yes
P25787	Yes
P25788	Yes
P25856	Yes
P26232	Yes
P26358	Yes
P26641	Yes
P27140	Yes

P27323	Yes
P27612	Yes
P27708	Yes
P28066	Yes
P28161	Yes
P28838	Yes
P29197	Yes
P29511	Yes
P29803	Yes
P30042	Yes
P30101	Yes
P31040	Yes
P31146	Yes
P31937	Yes
P31946	Yes
P31948	Yes
P34791	Yes
P34931	Yes
P35052	Yes
P35235	Yes
P35499	Yes
P36873	Yes
P37040	Yes
P37837	Yes
P38117	Yes
P38646	Yes
P39207	Yes
P40925	Yes
P40926	Yes
P40939	Yes
P42345	Yes
P42357	Yes
P42813	Yes
P43155	Yes
P43490	Yes
P45880	Yes
P45952	Yes
P47998	Yes
P48036	Yes
P48047	Yes
P48347	Yes
P48491	Yes
P48643	Yes
P49189	Yes

P49327	Yes
P49454	Yes
P49720	Yes
P50851	Yes
P50883	Yes
P50990	Yes
P51818	Yes
P53492	Yes
P53814	Yes
P54265	Yes
P54609	Yes
P54652	Yes
P55072	Yes
P56597	Yes
P56777	Yes
P60174	Yes
P60709	Yes
P60900	Yes
P61106	Yes
P61163	Yes
P61981	Yes
P62191	Yes
P62258	Yes
P62736	Yes
P62873	Yes
P63101	Yes
P63104	Yes
P63261	Yes
P68033	Yes
P68363	Yes
P68371	Yes
P78527	Yes
P78559	Yes
P81605	Yes
P93819	Yes
P94072	Yes
P98064	Yes
P98156	Yes
Q01813	Yes
Q01955	Yes
Q02383	Yes
Q02952	Yes
Q03265	Yes
Q06830	Yes

Q0WL92	Yes
Q0WR60	Yes
Q0WRR9	Yes
Q0WUV6	Yes
Q12931	Yes
Q12955	Yes
Q13011	Yes
Q13136	Yes
Q13283	Yes
Q13509	Yes
Q13535	Yes
Q13576	Yes
Q13618	Yes
Q13625	Yes
Q13748	Yes
Q13885	Yes
Q13939	Yes
Q14008	Yes
Q14315	Yes
Q14524	Yes
Q14566	Yes
Q14990	Yes
Q15149	Yes
Q15424	Yes
Q16555	Yes
Q16658	Yes
Q16698	Yes
Q2HIV2	Yes
Q33557	Yes
Q38946	Yes
Q3LXA3	Yes
Q3TJ94	Yes
Q3TRK3	Yes
Q41088	Yes
Q42029	Yes
Q43746	Yes
Q460N5	Yes
Q540M5	Yes
Q543S2	Yes
Q547G3	Yes
Q56ZK3	Yes
Q593N9	Yes
Q5JQC9	Yes
Q5JRA6	Yes

Q5SS40	Yes
Q5TZA2	Yes
Q5VST9	Yes
Q61344	Yes
Q64521	Yes
Q6AWV3	Yes
Q6GU14	Yes
Q6IA69	Yes
Q6JEL2	Yes
Q6P8J7	Yes
Q6PKC3	Yes
Q6UB99	Yes
Q6UVJ0	Yes
Q6Z8D9	Yes
Q6ZMR3	Yes
Q71U36	Yes
Q7Z4H7	Yes
Q84WT8	Yes
Q86UR5	Yes
Q86YZ3	Yes
Q8H0B7	Yes
Q8IV32	Yes
Q8IWV7	Yes
Q8N1C8	Yes
Q8N427	Yes
Q8NBX0	Yes
Q8NDH3	Yes
Q8NDX6	Yes
Q8NEB7	Yes
Q8NEZ4	Yes
Q8R081	Yes
Q8R4I4	Yes
Q8S9L5	Yes
Q8TAA3	Yes
Q8TD31	Yes
Q8TDR2	Yes
Q8TDY3	Yes
Q8TE82	Yes
Q8VXH1	Yes
Q8VXW1	Yes
Q8VY03	Yes
Q8W4H7	Yes
Q8WWI5	Yes
Q8WWK9	Yes

Q8WXH0	Yes
Q8WYR1	Yes
Q8WZ42	Yes
Q8WZ75	Yes
Q91VD9	Yes
Q91X86	Yes
Q92526	Yes
Q92736	Yes
Q92777	Yes
Q92781	Yes
Q92793	Yes
Q92820	Yes
Q92922	Yes
Q92945	Yes
Q93008	Yes
Q949U7	Yes
Q94BS2	Yes
Q94BT9	Yes
Q96FJ0	Yes
Q96HH9	Yes
Q96LI6	Yes
Q96ME7	Yes
Q96Q15	Yes
Q96QE4	Yes
Q96RW7	Yes
Q99250	Yes
Q99447	Yes
Q99666	Yes
Q99798	Yes
Q99996	Yes
Q99K10	Yes
Q99KD4	Yes
Q99LC5	Yes
Q99M47	Yes
Q9ASR0	Yes
Q9BS86	Yes
Q9BUF5	Yes
Q9BXM0	Yes
Q9BYJ4	Yes
Q9BYZ2	Yes
Q9CWS0	Yes
Q9D8L4	Yes
Q9EQN8	Yes
Q9EQV1	Yes

Q9FNE2	Yes
Q9FPF0	Yes
Q9FVT2	Yes
Q9FZ06	Yes
Q9H3G5	Yes
Q9H489	Yes
Q9H4A3	Yes
Q9H4B7	Yes
Q9H7X3	Yes
Q9H8N7	Yes
Q9LD57	Yes
Q9LF98	Yes
Q9LHA8	Yes
Q9LJE4	Yes
Q9LJX4	Yes
Q9LKA3	Yes
Q9LPW0	Yes
Q9LR30	Yes
Q9LUT2	Yes
Q9LZY8	Yes
Q9M7T0	Yes
Q9NQ38	Yes
Q9NS25	Yes
Q9NVA2	Yes
Q9NYC9	Yes
Q9P1W8	Yes
Q9P2E9	Yes
Q9P2Q2	Yes
Q9R158	Yes
Q9S7E7	Yes
Q9S7I3	Yes
Q9S841	Yes
Q9SA52	Yes
Q9SA56	Yes
Q9SF85	Yes
Q9SGT4	Yes
Q9SIB9	Yes
Q9SJU4	Yes
Q9SKP6	Yes
Q9SRV5	Yes
Q9SU69	Yes
Q9SUR0	Yes
Q9SW21	Yes
Q9SXJ7	Yes

Q9SYT0	Yes
Q9UBX3	Yes
Q9UI46	Yes
Q9UJ83	Yes
Q9UKU0	Yes
Q9ULB1	Yes
Q9ULW0	Yes
Q9UPA5	Yes
Q9UPN3	Yes
Q9UQ13	Yes
Q9WV42	Yes
Q9XEX2	Yes
Q9XFH8	Yes
Q9Y230	Yes
Q9Y265	Yes
Q9Y277	Yes
Q9Y2Q0	Yes
Q9Y5R2	Yes
Q9Y6L6	Yes
Q9Y6V0	Yes
Q9Y6Z4	Yes
Q9Z0L3	Yes
Q9ZP05	Yes
Q9ZP06	Yes
Q9ZR03	Yes
Q9ZRW8	Yes
Q9ZSK4	Yes
B5UB70	No
O22773	No
O75190	No
P04279	No
P06753	No
P07197	No
P10809	No
P21281	No
P25707	No
P28072	No
P28074	No
P29692	No
P31265	No
P36542	No
P42747	No
P46660	No
P55084	No

P56757	No
P58340	No
P61019	No
P63038	No
Q0WVE7	No
Q15631	No
Q16352	No
Q16772	No
Q1A7T7	No
Q3L1H0	No
Q3TDI5	No
Q3YAA6	No
Q8HT12	No
Q8LAS0	No
Q8LFV7	No
Q8N4E7	No
Q8VCI5	No
Q93045	No
Q9FX54	No
Q9LEV3	No
Q9NS26	No
Q9QUQ3	No
Q9S834	No
Q9SAJ4	No
Q9SCX3	No
Q9SIH0	No
Q9UHG2	No
Q9ULK5	No
