# Supplementary material for: iSNO-PseAAC: Predict Cysteine S-Nitrosylation Sites in Proteins by Incorporating Position Specific Amino Acid Propensity into Pseudo Amino Acid Composition
Source: PLoS One. 2013 Feb 7;8(2):e55844. doi: 10.1371/journal.pone.0055844 (PMC3567014; doi:10.1371/journal.pone.0055844)
Supplement: Supporting Information S3 — The detailed SNO sites detected by iSNO-PseAAC on an independent dataset with 461 nitrosylated proteins, of which 416 were predicted containing at least one SNO site. (PDF) [file pone.0055844.s003.pdf]

Online Supporting Information S3. The detailed SNO sites detected by iSNO-PseAAC on an independent dataset with 461 nitrosylated proteins, of which 416 were predicted containing at least one SNO site.

UniProt ID of the nitrosylated proteins	Predicted SNO site	Sequence (cf. Eq.1 of the paper)
A8MRZ7	84	QSGTGKTATFCSGVLQQLDFS
A8MRZ7	131	GDYLGVKVHACVGGTSVREDQ
A8MRZ7	260	EKEEWKLETLCDLYETLAITQ
O23255	86	ALGAEVRWCSCNIFSTQDHAA
O23255	244	TKSKFDNLYGCRHSLPDGLMR
O23255	268	VMIAGKVAVICGYGDVGKGCA
O23255	346	MRKMKNNAIVCNIGHFDNEID
O49485	86	KLLEDVANVDCSYNMTPEELN
O49485	102	PEELNIKISLCDALIVRSGTK
O49485	148	VDLSAATEFGCLVVNAPTANT
O50008	328	GKDKLVVSTSCSLLHTAVDLI
O50008	733	EQNILWVNPDCGLKTRKYTEV
O82660	7	XXXXMASLQLCDGYLLFKPSV
P10896	223	AADLIKKGKMCCLFINDLDAG
P10896	316	APTREDRIGVCKGIFRTDKIK
P22953	483	APRGVPQITVCFDIDANGILN
P25696	107	LDGTQNEWGWCKQKLGANAIL
P25696	124	NAILAVSLAVCKAGAVVSGIP
P25696	346	RVAKAIAEKSCNALLLKVNQI
P25697	46	TNRRFNTLITCAQETIVIGLA
P25856	79	FGRIGRNFLRCWHGRKDSPLD
P25856	348	DEPLVSVDFRCSDFSTTIDSS
P27140	230	ENIVVIGHSACGGIKGLMSFP
P27140	257	TDFIEDWVKICLPAKSKVISE
P29197	243	YFITNQKTQKCELDDPLILIH
P29197	304	LNKLRAGIKVCAIKAPGFGEN
P29511	118	YTIGKEIVDLCLDRIRKLADN
P29511	129	LDRIRKLADNCTGLQGFLVFN
P29511	347	TKRTIQFVDWCPTGFKCGINY
P29511	353	FVDWCPTGFKCGINYQPPTVV
P34791	75	YATTKHQRTACVKSMAAEEEE
P34791	131	PKTVENFRALCTGEKKYGYKG
P34791	206	PNTNGSQFFICTVKTSWLDNK
P34791	253	VPKKGCRIYACGELPLDAXXX
P42813	44	YFVQQWPGSYCDTQKKCCYPN
P42813	80	NYKDGTYPSNCDASKPFDSST
P42813	107	SMKKSWPTLACPSGSGEAFWE
P47998	28	LVYLNNVAEGCVGRVAAKLEM
P47998	42	VAAKLEMMEPCSSVKDRIGFS
P48347	98	KRVEDELAKVCNDILSVIDKH
P48347	193	YEILNSPESACQLAKQAFDDA
P48491	13	RKFFVGGNWKCNGTAEEVKKI
P48491	67	RSDFFVAAQNCWVKKGGAFTG
P48491	127	ALAQGLKVIACVGETLEEREA
P51818	345	YVRRVFIMDNCEDIIPEYLGF
P51818	391	KVIRKNLVKKCLELFFEIAEN
P51818	543	EELKEKFEGLCKVIKDVLGDK

P51818	569	VSDRVVDSPCCLVTGEYGWTA
P53492	12	ADGEDIQPLVCDNGTGMVKAG
P53492	259	VITIGAERFRCPEVLFQPSLI
P54609	73	IKGKKRKDTVCIALADETCEE
P54609	81	TVCIALADETCEEPKIRMNKV
P54609	109	RLGDVISVHQCPDVKYGKRVH
P54609	188	CVVAPDTEIFCEGEPVKREDE
P54609	271	VANETGAFFFCINGPEIMSKL
P54609	525	KGVLFYGPPGCGKTLLAKAIA
P54609	538	TLLAKAIANECQANFISVKGP
P54609	575	IFDKARQSAPCVLFFDELDSI
P54609	699	ADITEICQRACKYAIRENIEK
P56777	107	AGAHIVFSGLCFLAAIWHWVY
P56777	124	HWVYWDLEIFCDERTGKPSLD
Q0WL92	31	KSAIHSFPAQCSSKRLEVAEF
Q0WL92	99	FGRIGRNFLRCWHGRKDSPLE
Q0WL92	360	NGPMKGILDVCDAPLVSVDFR
Q0WR60	33	SHLSPFSFSRCFSTVLEGLKY
Q0WRR9	40	PNTAENFRALCTGEKGMGKLG
Q0WRR9	69	IFHRVIPGFMCQGGDFTAKNG
P22954	483	APRGVPQITVCFDIDANGILN
Q42029	236	ATVNGGKLYICKAQAGDKRWF
Q547G3	39	GGHIVIKNRPCKVVEVSTSKT
Q547G3	80	LEDIVPSSHNCDVPHVNRVDY
Q547G3	150	VMSSMGEEQICAVKEVGGGKX
Q84WT8	8	XXXMMMKSLICLSLILLPLVS
P17745	149	TENRHYAHVDCPGHADYVKNM
P27323	346	YVRRVFIMDNCEELIPEYLSF
P27323	545	EEKKKSFENLCKTIKEILGDK
P27323	570	VVSDRIVDSPCCLVTGEYGWT
P27323	571	VSDRIVDSPCCLVTGEYGWTA
Q9SIB9	146	RILLESAIRNCDNFQVTKEDV
Q9SIB9	553	LGAGLVAKKACDLGLQVKPWI
Q9SIB9	602	FNIVGYGCTTCIGNSGEINES
Q9SIB9	914	NLVGMGIIPLCFKSGEDADTL
Q9SIB9	961	VTTDNGKSFTCTVRFDTEVEL
Q8S9L5	246	LGDEIDAKKACERQLRQKYKS
Q8S9L5	377	DSLADKLLPGCTTLKEVEETL
Q8VY03	95	FWMDKNGLPPCKVILKERPAH
Q8VY03	329	YKAGDPIVVWCGPQPNAKLLL
Q8VY03	486	VQLEKKILVACLTTTVDLLNQ
Q8VY03	505	NQLPDTAISPCPAPYAPSLKX
Q949U7	121	FAVPGAFTPTCSQKHVPGFVS
Q949U7	146	LRSKGIDVIACISVNDAFVME
Q94BS2	262	EASVASYNVACCYSKLNQVQA
Q94BT9	46	VLRVAMTCEGCVGAVKRVLGK
Q9ASR0	12	REILHIQGGQCGNQIGAKFWE
Q9ASR0	129	VVRKEAENCDCLQGFQVCHSL
Q9ASR0	211	VLDNEALYDICFRTLKLTTPS
Q9ASR0	354	WIPNNVKSTVCDIPPTGLKMA
Q9FPF0	49	SVETQVGVDACHGIKMVADTL
Q9FPF0	107	DSDGRLNAAICCAPALALGTW
Q9FZ06	772	RMVAGAIANLCGNDKLQTKLR
Q9FZ06	796	GIAALLGMVRCGHPDVLAQVA
Q9FZ06	815	VARGIANFAKCESRASTQGTK

Q9FZ06	860	AIRRHIELALCHLAQHEGNAK
Q9LF98	326	KAAQEALYVRCKANSEATLGT
Q9LJE4	50	CVRRSRPAIVCAAKELHFNKD
Q9LJE4	467	VEEGIVVGGGCTLLRLASKVD
Q9LJX4	715	MVSLSLQMYGCRVIQKALEVI
Q9LJX4	805	EHCSDDEETHCIIDEILESAF
Q9LKA3	137	KNLSIAIAKYCPQALVNMISN
Q8W4H7	111	NMITGTSQADCAVLIIDSTTG
Q8W4H7	151	FTLGVKQMICCCNKMDATTPK
Q9LPW0	82	FGRIGRNFLRCWHGRKDSPLD
Q9LPW0	216	HEDTIISNASCTTNCLAPFVK
Q9LZY8	372	PFQVPDDVKSCLDDLLFDLHF
Q9LZY8	508	GVREHGMGAICNGIALHSPGL
Q9S7E7	183	IGMTRQELVDCLGTIAQSGTA
Q9S7E7	515	DNFGKHLKLGCIEDRENHKRI
Q9S7E7	640	AAVKKEFGQTCDWIKKRLGDK
Q9S7I3	53	LTRGAPLTQGCCNGVTNLKNM
Q9S7I3	54	TRGAPLTQGCCNGVTNLKNMA
Q9S7I3	100	TARAAGLPSACKVNIPYKISA
Q9S841	48	GLDSSQARLTCSLHSDLKDFA
Q9S841	136	KAGKYTGKKFCFEPTSFTVKA
Q9SGT4	139	LVVVDCIEGVCVQTETVLRQA
Q9SGT4	166	PVLTVNKMDRCFLELQVDGEE
Q9SGT4	278	PTCKRGFVQFCYEPIKQIIAT
Q9SGT4	289	YEPIKQIIATCMNDQKDKLWP
Q9SGT4	373	DDQYANAIRNCDPNGPLMLYV
Q9SGT4	498	VSPVVRVAVQCKVASDLPKLV
Q9SGT4	572	DPVVSFRETVCDRSTRTVMSK
Q9SKP6	74	GKFFVGGNWKCNGTKDSIAKL
Q9SKP6	148	ISVEQLKDLGCKWVILGHSER
Q9SKP6	186	ALSEGLGVIACIGEKLEEREA
Q9SKP6	203	EREAGKTFDVCFAQLKAFADA
Q9SRV5	328	GKDKLVVSTSCSLLHTAVDLI
Q9SRV5	733	EQNILWVNPDCGLKTRKYTEV
Q9SU69	56	KDGRKISVGDCALFKPPQDCP
Q9SU69	65	DCALFKPPQDCPPFIGIIRLI
Q9SU69	181	DRQLEVDKLLCKTRSEMHTTL
Q9SU69	315	GFVAATNRFDCLSRFVQLRGL
Q9SU69	377	LPVNLNALQTCNIGKSVNHLR
Q9SU69	468	VKQQVENNLKCVATSPGSTRS
Q9SU69	662	SPVHTVKQELCDNNWREKNHS
Q9SU69	928	IKTDVKSEADCTSDLTKRVAS
Q9SU69	1281	ASQRSGNPTNCTSGITNNFDQ
Q9SUR0	112	YEFIPKSEDSCVCKITMIWEK
Q9SUR0	114	FIPKSEDSCVCKITMIWEKRN
Q9SW21	49	STKSLRLQISCAAKAETVQKV
Q9SXJ7	109	KSSGREKASRCVPKAMFERFT
Q9SXJ7	320	ILARRTKNNPCLIGEPGVGKT
Q9SXJ7	426	KPALARGELQCIGATTIDEYR
Q9SYT0	111	SSNQVLMEVACTRTSTQLLHA
Q9SYT0	239	FLALLRSTIQCLTRPELYFVD
Q9XFH8	126	YDDVVFLKLDCNPDNRPLAKE
Q9ZP05	143	KINAGIVKTLCEGVAKCCPNA
Q9ZP05	150	KTLCEGVAKCCPNAIVNLISN
Q9ZR03	49	RKEKIGLRISCQASSIPADRV

Q9ZR03	157	TLATYGINAVCTHLGCVVPWN
Q9ZR03	162	GINAVCTHLGCVVPWNKAENK
Q9ZR03	177	NKAENKFLCPCHGSQYNAQGR
Q9LHA8	483	APRGVPQITVCFDIDANGILN
Q9ZSK4	14	AASGMAVHDDCKLKFMELKTK
P48643	253	VEDAKIAILTCPFEPPKPKTK
P48643	302	IKETGANLAICQWGFDDEANH
P48643	407	EAKRSLHDALCVIRNLIRDNR
P31146	40	VSQTTWDSGFCAVNPKFVALI
P31146	78	GRVDKNAPTVCGHTAPVLDIA
P31146	195	SRDGGLICTSCRDKRVRIIEP
P31146	332	MPKRGLEVNKCEIARFYKLHE
B4DX73	33	LTQKKVLRAPCGAPSVTVTKS
B4DX73	88	VGCKWENPPHCLEITPPSSEK
B4DX73	136	KIDSLMNAVGCLKSEVKMQKG
B4DX73	243	GKLKTEKQMTCTDINTLTRQK
B4DX73	567	ADEVAAQLERCDKENKILKDE
O14556	21	VTVVQLLRQPCPVTRAPPPPE
O14556	150	VDNHEISVYQCKEPKQIPWRA
O14645	57	PPKTKLPSTPCVPDPTKQAEE
O14645	121	QQRQARETGICPVRRELYSQC
O14645	214	ERQVNEQKAKCEATEKRESER
O14715	50	KEYDLAKKYICTYINVQERDP
O14715	105	DLVLKIAELLCKNDVTDGRAK
O14715	521	YQPLCLPFPVCKQLCTERQKS
O14715	537	ERQKSWWDAVCTLIHRKAVPG
O14715	1108	LMRREQVLKVCANHWITTTMN
O14715	1405	VMRRDQVLKLCANHRITPDMS
O14715	1431	GTERVWVWTACDFADGERKVE
O15027	497	NMETLCAPQVCPLPLNSTTEA
O15027	540	PSARTQGPVKCESPATTLWAQ
O15027	1115	DPRTYDRRYWCDAEYDAYRRE
O15027	1514	MSGRMPAASTCCGDEKWGDWR
O15027	1726	WHQDGALPQQCPGTPSSEMEQ
O43374	30	KDITGSSDPYCIVKVDNEPII
O43374	88	LSRDDVIGKVCLTRDTIASHP
O43374	176	TRETSIVKKSCYPRWNETFEF
O43374	396	PSKVEVKDVGCSGLHRPQTEA
O43374	655	AGRPQTAYLQCKCVNELNQWL
O43374	694	PGVFRGDKWSCCHQKEKTGQG
O43374	695	GVFRGDKWSCCHQKEKTGQGC
O43374	705	CHQKEKTGQGCDKTRSRVTLQ
O43933	27	VTVAFTNARDCFLHLPRRLVA
O43933	629	KLDAHVERVDCKALRGKRLEN
O43933	733	VSAQGVHIFQCVQHIQPPNQE
O43933	750	PNQEQRCEILCNVIKNKLDCD
O43933	759	LCNVIKNKLDCDINKFTDLDL
O43933	1005	RPGRLDKCVYCPPPDQVSRLE
O60309	946	LPFLKFINLSCNVITELSFGT
O60309	1039	CQFKNSIEAVCKTVKLHCNSA
O60309	1050	KTVKLHCNSACLTNTTHCPEE
O60309	1600	GILTILIILLCLIEICCHRRS
O60437	311	DWKEYLNLLICEESHLKYMED
O60437	449	AGNKLIAPAVCFVIPPTDPEA
O60437	1265	IVDKTRLIERCDLEIYQLKKE

O75037	67	DTWQEQIYSTCVSKLIEGCFE
O75037	641	NFQADLADLTCEIEIKQKLID
O75037	999	IDYINDGITDCQATIVQLEET
O75037	1382	KVWDIRDSAKCIRTLTSSGQV
O75037	1398	SSGQVISGDACAATSTRAITS
O75037	1488	HYVKMFELGECVTGTIGPTHN
O75037	1621	LWNYVPGLTPCLPRRVLAIKG
O75179	58	ASSPRGMVRVCDLLLKKKPPQ
O75179	210	VLRRLTSSVSCALDEAAAALT
O75179	409	EFKESALTLACYKGHLEMVRF
O75179	476	SFESPLTLAACGGHVELAALL
O75179	542	ETQETALTLACCGGFLEVADF
O75179	543	TQETALTLACCGGFLEVADFL
O75179	564	IKAGADIELGCSTPLMEAAQE
O75179	605	ATGDTALTYACENGHTDVADV
O75179	644	LMKAARAGHVCTVQFLISKGA
O75179	672	NNDHTVLSLACAGGHLAVVEL
O75179	714	AAKGGHTSVVCYLLDYPNNLL
O75179	1091	SNHDTALTLACAGGHEELVQT
O75179	1158	RTKDTPLSLACSGGRQEVVEL
O75179	1260	TNRNTALTLACFQGRTEVVSL
O75179	1336	IAADKGHYKFCELLIGRGAHI
O75179	1437	DKEMLKKCHLCMESIVQAKDR
O75179	1746	VISRVIGRGGCNINAIREFTG
O75369	26	KIQQNTFTRWCNEHLKCVNKR
O75369	416	VEDKGNQVYRCVYKPMQPGPH
O75369	604	IEYNDQNDGSCDVKYWPKEPG
O75369	660	AYGPGLEKSGCIVNNLAEFTV
O75369	706	MKNRMDGTYACSYTPVKAIKH
O75369	991	ILSPSRKVVPCLVTPVTGREN
O75369	1081	GGLGLTVEGPCEAKIECSDNG
O75369	1326	SPFKVAVTEGCQPSRVQAQGP
O75369	1650	AKTAGKGKVTCTVLTPDGTEA
O75369	1720	TAVEEAPVNACPPGFRPWVTE
O75369	1876	ISCIDNKDGTCTVTYLPTLPG
O75369	2333	VHSPSGAVEECHVSELEPDKY
O75952	82	EGTTPQKKLECLKEPGKTSVE
O75952	226	EDVMVAAPLVCSGKVLEVQVV
O75952	326	IVIPFTDQVACLKENEQSKEN
O75969	15	VDWLQSQNGVCKVDVYSPGDN
O75969	108	HFEMTHKEIPCQGPRAQLGNG
O75969	154	NEKIDGSENKCVYQSLYMGNE
O75969	688	MKLCVIIAKSCDASLAELGDD
O75969	717	TSAFPDSLYECLPAKGTGSAE
O75969	815	QLSAAAVDKGCSVGEVLQSVL
O94769	103	YNVLPGKKGHCLVKGITMYNK
O94769	504	NNQIEEITEICFNHTRKINVI
O95271	270	GHAEVVSLLLCQGADPNARDN
O95271	298	EAAIKGKIDVCIVLLQHGADP
O95271	362	MALLTPLNVNCHASDGRKSTP
O95271	528	QPQSHETALHCAVASLHPKRK
O95271	766	EAAAKGKYEICKLLLKHGADP
O95271	815	AALLDAAKKGCLARVQKLCTP
O95271	1163	VVNKKLRERFCHRQKEVSEEN
O95271	1234	YVYGIGGGTGCPTHKDRSCYI

O95271	1252	CYICHRQMLFCRVTLGKSFLQ
O95861	42	EGDLGIVEKTCATDLQTKADR
O95861	59	KADRLAQMSICSSLARKFPKL
O95861	243	ASAYVFASPGCKKWDTCAPEV
P00338	35	VVGVGAVGMACAISILMKDLA
P00338	131	IPNVVKYSPNCKLLIVSNPVD
P00367	112	VRGILRIIKPCNHVLSLSFPI
P00367	146	RAQHSQHRTPCKGGIRYSTDV
P00367	376	PYEGSILEADCDILIPAASEK
P00558	99	LGKDVLFLKDCVGPEVEKACA
P00558	367	DEVVKATSRGCITIIGGGDTA
P00558	379	TIIGGGDTATCCAKWNTEDKV
P00558	380	IIGGGDTATCCAKWNTEDKVS
P00747	49	KQLGAGSIEECAAKCEEDEEF
P00747	61	AKCEEDEEFTCRAFQYHSKEQ
P00747	73	AFQYHSKEQQCVIMAENRKSS
P00747	103	LFEKKVYLSECKTGNGKNYRG
P00747	124	TMSKTKNGITCQKWSSTSPHR
P00747	164	NPDNDPQGPWCYTTDPEKRYD
P00747	176	TTDPEKRYDYCDILECEEECM
P00747	206	KISKTMSGLECQAWDSQSPHA
P00747	234	FPNKNLKKNYCRNPDRELRPW
P00747	245	RNPDRELRPWCFTTDPNKRWE
P00747	316	THNRTPENFPCKNLDENYCRN
P00747	324	FPCKNLDENYCRNPDGKRAPW
P00747	335	RNPDGKRAPWCHTTNSQVRWE
P00747	426	YPNAGLTMNYCRNPDADKGPW
P00747	449	TTDPSVRWEYCNLKKCSGTEA
P00747	502	KRATTVTGTPCQDWAAQEPHR
P00747	543	NPDGDVGGPWCYTTNPRKLYD
P00747	555	TTNPRKLYDYCDVPQCAAPSF
P00747	560	KLYDYCDVPQCAAPSFDCGKP
P00747	567	VPQCAAPSFDCGKPQVEPKKC
P00747	607	SLRTRFGMHFCGGTLISPEWV
P02768	114	LRETYGEMADCCAKQEPERNE
P02768	125	CAKQEPERNECFLQHKDDNPN
P02768	148	RLVRPEVDVMCTAFHDNEETF
P02768	192	AKRYKAAFTECCQAADKAACL
P02768	193	KRYKAAFTECCQAADKAACLL
P02768	201	ECCQAADKAACLLPKLDELRD
P02768	224	KASSAKQRLKCASLQKFGERA
P02768	302	QDSISSKLKECCEKPLLEKSH
P02768	340	AADFVESKDVCKNYAEAKDVF
P02768	393	KCCAAADPHECYAKVFDEFKP
P02768	416	EEPQNLIKQNCELFEQLGEYK
P02768	538	AETFTFHADICTLSEKERQIK
P02768	583	DDFAAFVEKCCKADDKETCFA
P02768	591	KCCKADDKETCFAEEGKKLVA
P02787	58	VIPSDGPSVACVKKASYLDCI
P02787	67	ACVKKASYLDCIRAIAANEAD
P02787	156	GWNIPIGLLYCDLPEPRKPLE
P02787	246	KADRDQYELLCLDNTRKPVDE
P02787	350	TAIRNLREGTCPEAPTDECKP
P02787	387	WSVNSVGKIECVSAETTEDCI
P02787	421	DGGFVYIAGKCGLVPVLAENY

P02787	503	CRFDEFFSEGCAPGSKKDSSL
P02787	542	GYYGYTGAFRCLVEKGDVAFV
P02787	582	NLNEKDYELLCLDGTRKPVEE
P02787	615	AVVTRKDKEACVHKILRQQQH
P02787	639	SNVTDCSGNFCLFRSETKDLL
P02787	656	KDLLFRDDTVCLAKLHDRNTY
P02787	684	YVKAVGNLRKCSTSSLLEACT
P02788	17	VLLFLGALGLCLAGRRRSVQW
P02788	64	SCIKRDSPIQCIQAIAENRAD
P02788	250	EAERDEYELLCPDNTRKPVDK
P02788	367	VAARRARVVWCAVGEQELRKC
P02788	390	WSGLSEGSVTCSSASTTEDCI
P02788	424	DGGYVYTAGKCGLVPVLAENY
P02788	594	DLKLADFALLCLDGKRKPVTE
P02788	646	QAKFGRNGSDCPDKFCLFQSE
P02788	668	KNLLFNDNTECLARLHGKTTY
P02788	696	YVAGITNLKKCSTSPLLEACE
P04075	150	KDGADFAKWRCVLKIGEHTPS
P04075	290	ASINLNAINKCPLLKPWALTF
P04075	339	VKRALANSLACQGKYTPSGQA
P04406	152	NSLKIISNASCTTNCLAPLAK
P04406	247	TANVSVVDLTCRLEKPAKYDD
P04637	124	LHSGTAKSVTCTYSPALNKMF
P04637	277	RNSFEVRVCACPGRDRRTEEE
P05091	66	TVNPSTGEVICQVAEGDKEDV
P05091	320	ALFFNQGQCCCAGSRTFVQED
P05091	386	TGKQEGAKLLCGGGIAADRGY
P06396	12	APHRPAPALLCALSLALCALS
P06396	215	VSWESFNNGDCFILDLGNNIH
P06396	672	MDAHPPRLFACSNKIGRFVIE
P06733	119	NAILGVSLAVCKAGAVEKGVP
P06733	337	RIAKAVNEKSCNCLLLKVNQI
P06733	389	TFIADLVVGLCTGQIKTGAPC
P07195	36	VVGVGQVGMACAISILGKSLA
P07195	132	IPQIVKYSPDCIIIVVSNPVD
P07205	99	LGKDVLFLKDCVGAEVEKACA
P07205	367	DEIVKATSKGCITVIGGGDTA
P07205	379	TVIGGGDTATCCAKWNTEDKV
P07205	380	VIGGGDTATCCAKWNTEDKVS
P07237	53	YLLVEFYAPWCGHCKALAPEY
P07237	56	VEFYAPWCGHCKALAPEYAKA
P07237	397	NVFVEFYAPWCGHCKQLAPIW
P07864	21	KLIEDDENSQCKITIVGTGAV
P07864	35	IVGTGAVGMACAISILLKDLA
P07900	374	YVRRVFIMDNCEELIPEYLNF
P07900	420	KVIRKNLVKKCLELFTELAED
P07900	572	EEKKTKFENLCKIMKDILEKK
P07900	598	VSNRLVTSPCCIVTSTYGWTA
P07947	248	KHYTEHADGLCHKLTTVCPTV
P07947	255	DGLCHKLTTVCPTVKPQTQGL
P07947	410	ANILVGENLVCKIADFGLARL
P07947	497	RGYRMPCPQGCPESLHELMNL
P07954	333	ELSGAMNTTACSLMKIANDIR
P07954	434	GDASVSFTENCVVGIQANTER
P08107	603	EHKRKELEQVCNPIISGLYQG

P08238	366	YVRRVFIMDSCDELIPEYLNF
P08238	412	KVIRKNIVKKCLELFSELAED
P08238	564	EESKAKFENLCKLMKEILDKK
P08238	590	ISNRLVSSPCCIVTSTYGWTA
P08670	328	EYRRQVQSLTCEVDALKGTNE
P09622	9	XXMQSWSRVYCSLAKRGHFNR
P09622	69	KAAQLGFKTVCIEKNETLGGT
P09622	85	TLGGTCLNVGCIPSKALLNNS
P09622	312	EVITCDVLLVCIGRRPFTKNL
P09622	484	GASCEDIARVCHAHPTLSEAF
P10323	25	VSVVAKDNATCDGPCGLRFRQ
P10323	89	NSRWVLTAAHCFVGKNNVHDW
P10323	162	ISCGRFIGPGCLPHFKAGLPR
P10323	209	ARVDLIDLDLCNSTQWYNGRV
P10323	225	YNGRVQPTNVCAGYPVGKIDT
P10515	291	TRDVPLGTPLCIIVEKEADIS
P10515	488	DFIIKASALACLKVPEANSSW
P10515	586	FSAIINPPQACILAIGASEDK
P11142	17	VGIDLGTTYSCVGVFQHGKVE
P11142	574	DEDKQKILDKCNEIINWLDKN
P11142	603	EHQQKELEKVCNPIITKLYQS
P11169	131	ILGRLVIGLFCGLCTGFVPMY
P11169	205	AILQSAALPFCPESPRFLLIN
P11169	366	KDNYNGMSFVCIGAILVFVAF
P12110	37	SPDTTERNNNCPEKTDCPIHV
P12110	43	RNNNCPEKTDCPIHVYFVLDT
P12110	170	ITDGHVTGSPCGGIKLQAERA
P12110	777	HESENLYSIACDKPQQVRNMT
P12110	806	KFIDDMEDVLCPDPQIVCPDL
P12532	62	EYPDLRKHNNCMASHLTPAVY
P12532	76	HLTPAVYARLCDKTTPTGWTL
P12532	287	GNMKRVFERFCRGLKEVERLI
P12532	316	WNERLGYILTCPSNLGTGLRA
P12883	122	WMIYTYSGLFCVTVNPYKWLP
P12883	400	LNSADLLKGLCHPRVKVGNEY
P12883	520	FIDFGMDLQACIDLIEKPMGI
P12883	672	LRSTHPHFVRCIIPNETKSPG
P12883	905	QDNLADAEERCDQLIKNKIQL
P12883	947	TAKKRKLEDECSELKRDIDDL
P13639	41	GKSTLTDSLVCKAGIIASARA
P13639	136	LVVVDCVSGVCVQTETVLRQA
P13639	290	PEGKKLPRTFCQLILDPIFKV
P13639	388	DDEAAMGIKSCDPKGPLMMYI
P13639	536	RLAKSDPMVQCIIEESGEHII
P13639	567	CLKDLEEDHACIPIKKSDPVV
P13639	591	ETVSEESNVLCLSKSPNKHNR
P13639	651	WDVAEARKIWCFGPDGTGPNI
P13639	728	GGQIIPTARRCLYASVLTAQP
P14136	294	DYRRQLQSLTCDLESLRGTNE
P14618	165	NILWLDYKNICKVVEVGSKIY
P14618	326	RCNRAGKPVICATQMLESMIK
P14618	424	VGAVEASFKCCSGAIIVLTKS
P14618	474	HLYRGIFPVLCKDPVQEAWAE
P14625	10	XMRALWVLGLCCVLLTFGSVR
P14625	138	GNEELTVKIKCDKEKNLLHVT

P15104	42	WIDGTGEGLRCKTRTLDSEPK
P15104	53	KTRTLDSEPKCVEELPEWNFD
P15104	99	FRKDPNKLVLCEVFKYNRRPA
P15104	229	WVARFILHRVCEDFGVIATFD
P15104	252	PIPGNWNGAGCHTNFSTKAMR
P15104	346	YFEDRRPSANCDPFSVTEALI
P15104	359	FSVTEALIRTCLLNETGDEPF
P15259	153	AGLKPGELPTCESLKDTIARA
P15924	405	LQDSIRKKYPCDKNMPLQHLL
P15924	467	SNKPIILRALCDYKQDQKIVH
P15924	482	DQKIVHKGDECILKDNNERSK
P15924	524	PPNPLAVDLSCKIEQYYEAIL
P15924	553	NMKSLVSWHYCMIDIEKIRAM
P15924	682	LQKIRRQIEHCEGRMTLKNLP
P15924	768	GVTDGYLNSLCTVRALLQAIL
P15924	798	EARLTEEETVCLDLDKVEAYR
P15924	809	LDLDKVEAYRCGLKKIKNDLN
P15924	899	RNYRDNYQAFCKWLYDAKRRQ
P15924	1052	RLARDANSENCNKNKFLDQNL
P15924	1069	DQNLQKYQAECSQFKAKLASL
P15924	1100	GKSAKQNLDKCYGQIKELNEK
P15924	1148	DYDQLQKARQCEKENLGWQKL
P15924	1280	AEENALQQKACGSEIMQKKQH
P15924	1589	RLKRTASEDSCKRKKLEEELE
P15924	1855	AETRVKQRLECEKQQIQNDLN
P15924	1957	YGSHRETQTECEWTVDTSKLV
P15924	1983	KKVTAMQLYECQLIDKTTLDK
P15924	2259	RIKDFLQGSSCIAGIYNETTK
P15924	2442	RCIKDEETGLCLLPLKEKKKQ
P15924	2656	TGQRLLEAQACTGGIIHPTTG
P15924	2776	DTSSYAKILTCPKTKLKISYK
P16152	226	RKGDKILLNACCPGWVRTDMA
P16152	227	KGDKILLNACCPGWVRTDMAG
P17066	19	VGIDLGTTYSCVGVFQQGRVE
P17174	253	YFVSEGFEFFCAQSFSKNFGL
P17661	333	EYRHQIQSYTCEIDALKGTND
P17987	76	EVEHPAAKVLCELADLQDKEV
P17987	125	TSVISGYRLACKEAVRYINEN
P17987	218	SMLISGYALNCVVGSQGMPKR
P17987	236	PKRIVNAKIACLDFSLQKTKM
P17987	357	QAEEVVQERICDDELILIKNT
P17987	397	EMERSLHDALCVVKRVLESKS
P18669	153	ADLTEDQLPSCESLKDTIARA
P19367	133	GSQLFDHVAECLGDFMEKRKI
P19367	158	LPVGFTFSFPCQQSKIDEAIL
P19367	375	DDDCVSVQHVCTIVSFRSANL
P19367	606	MPLGFTFSFPCQQTSLDAGIL
P19367	628	TWTKGFKATDCVGHDVVTLLR
P19367	823	CDDSILVKTVCGVVSRRAAQL
P21266	91	LRYIARKHNMCGETEEEKIRV
P21266	119	MDFRTQLIRLCYSSDHEKLKP
P21333	53	KIQQNTFTRWCNEHLKCVSKR
P21333	59	FTRWCNEHLKCVSKRIANLQT
P21333	444	LEARGDSTYRCSYQPTMEGVH
P21333	574	SPFEVKVGTECGNQKVRAWGP

P21333	717	LRVQVQDNEGCPVEALVKDNG
P21333	1157	GSPFKAHVVPCFDASKVKCSG
P21333	1165	VPCFDASKVKCSGPGLERATA
P21333	1225	GTHTITYIPLCPGAYTVTIKY
P21333	1353	SPFQVPVTEGCDPSRVRVHGP
P21333	1453	HDVTDASKVKCSGPGLSPGMV
P21333	1997	VVPPSGREEPCLLKRLRNGHV
P21333	2378	VHSPSGALEECYVTEIDQDKY
P21333	2543	VFVDSLTKATCAPQHGAPGPG
P23368	190	PVGKLCLYTACAGIRPDRCLP
P23368	481	FPGVALAVILCNTRHISDSVF
P23458	74	AAQACRISPLCHNLFALYDEN
P23458	169	FAQGQYDLVKCLAPIRDPKTE
P23458	189	EQDGHDIENECLGMAVLAISH
P23458	257	FLKEFNNKTICDSSVSTHDLK
P23458	440	PLIVHNIQNGCHGPICTEYAI
P23458	481	DFDNILMTVTCFEKSEQVQGA
P23458	543	TDNISFMLKRCCQPKPREISN
P23458	544	DNISFMLKRCCQPKPREISNL
P23458	716	EDKDLVHGNVCTKNLLLAREG
P23458	731	LLAREGIDSECGPFIKLSDPG
P23458	763	IERIPWIAPECVEDSKNLSVA
P23458	944	HENIVKYKGICTEDGGNGIKL
P23458	1116	NTLKEGKRLPCPPNCPDEVYQ
P23458	1120	EGKRLPCPPNCPDEVYQLMRK
P24539	239	TQQEKETIAKCIADLKLLAKK
P24752	196	DVYNKIHMGSCAENTAKKLNI
P25788	42	ENSSTAIGIRCKDGVVFGVEK
P26232	335	DDRRERIVAECNAVRQALQDL
P26232	436	NKLVEVANLACSISNNEEGVK
P26232	459	RMAATQIDSLCPQVINAALTL
P26232	766	DKLARAVADQCPDSACKQDLL
P26232	825	STFTTFYEVDCDVIDGGRASQ
P26358	41	LERDSLTEKECVKEKLNLLHE
P26358	62	FLQTEIKNQLCDLETKLRKEE
P26358	409	YEALPQHKLTCFSVYCKHGHL
P26358	420	FSVYCKHGHLCPIDTGLIEKN
P26358	656	NAFKRRRCGVCEVCQQPECGK
P26358	659	KRRRCGVCEVCQQPECGKCKA
P26358	751	DGKKSYYKKVCIDAETLEVGD
P26358	798	NGQMFHAHWFCAGTDTVLGAT
P26358	896	EDNKFKFCVSCARLAEMRQKE
P26358	1001	YRIGRIKEIFCPKKSNGRPNE
P26358	1060	VVDFKAVQGRCTVEYGEDLPE
P26358	1125	KGKGKPKSQACEPSEPEIEIK
P26358	1148	KLRTLDVFSGCGGLSEGFHQA
P26358	1286	RSMVLKLTLRCLVRMGYQCTF
P26358	1499	ARQFNTLIPWCLPHTGNRHNH
P26358	1553	EQHRVVSVRECARSQGFPDTY
P26641	166	RVTLADITVVCTLLWLYKQVL
P26641	194	FPNTNRWFLTCINQPQFRAVL
P26641	210	FRAVLGEVKLCEKMAQFDAKK
P27708	183	GGAPRILALDCGLKYNQIRCL
P27708	252	EPNPRPVFGICLGHQLLALAI
P27708	288	QPCLLVGSGRCFLTSQNHGFA

P27708	379	TVRERLTERLCPPGIPTPGSG
P27708	484	LTFGGQTALNCGVELTKAGVL
P27708	665	VTQHLGIVGECNVQYALNPES
P27708	736	TAAFEPSVDYCVVKIPRWDLS
P27708	897	VRKLRQELGICPAVKQIDTVA
P27708	1215	AKDDQLKVIECNVRVSRSFPF
P27708	1374	WHFEEAVDGECPPQRSILEQL
P27708	1432	FSVPLIIDIKCTKLFVEALGQ
P27708	1613	AQLTQRSVHICHVARKEEILL
P27708	2092	KHGRTVHSLACLLTQYRVSLR
P28066	47	AIGIQTSEGVCLAVEKRITSP
P28066	165	HMDPSGTFVQCDARAIGSASE
P29803	143	LAELTGRRGGCAKGKGGSMHM
P29803	179	GPLGAGIALACKYKGNDEICL
P29803	220	ALWKLPCVFICENNLYGMGTS
P30042	153	LSTFAVDGKDCKVNKEVERVL
P30042	176	FHQAGKPIGLCCIAPVLAAKV
P30042	177	HQAGKPIGLCCIAPVLAAKVL
P30101	57	LMLVEFFAPWCGHCKRLAPEY
P30101	85	KGIVPLAKVDCTANTNTCNKY
P30101	244	KFIQENIFGICPHMTEDNKDL
P30101	406	DVLIEFYAPWCGHCKNLEPKY
P31040	89	GLSEAGFNTACVTKLFPTRSH
P31040	189	KFGKGGQAHRCCCVADRTGHS
P31040	231	ALDLLMENGECRGVIALCIED
P31040	266	TGGYGRTYFSCTSAHTSTGDG
P31040	305	FHPTGIYGAGCLITEGCRGEG
P31040	357	MTLEIREGRGCGPEKDHVYLQ
P31040	467	LLDLVVFGRACALSIEESCRP
P31040	536	FRVGSVLQEGCGKISKLYGDL
P31937	211	AVGTGQAAKICNNMLLAISMI
P31937	251	AKILNMSSGRCWSSDTYNPVP
P31946	96	EKIEAELQDICNDVLELLDKY
P31946	191	YEILNSPEKACSLAKTAFDEA
P31948	62	GDYQKAYEDGCKTVDLKPDWG
P31948	282	KGDYNKCRELCEKAIEVGREN
P31948	339	EHRTPDVLKKCQQAEKILKEQ
P31948	403	DAKLYSNRAACYTKLLEFQLA
P34931	605	DHKRKELEQMCNPIITKLYQG
P34931	617	PIITKLYQGGCTGPACGTGYV
P35052	191	QLLLPDDYLDCLGKQAEALRP
P35052	259	AVMKLVYCAHCLGVPGARPCP
P35052	343	DTLTAKVIQGCGNPKVNPQGP
P35052	534	QEGQKTSAASCPQPPTFLLPL
P35499	182	SLIKILARGFCVDDFTFLRDP
P35499	560	KCAHKVLIWNCCAPWLKFKNI
P35499	561	CAHKVLIWNCCAPWLKFKNII
P35499	587	DPFVDLGITICIVLNTLFMAM
P35499	729	MQLFGKSYKECVCKIALDCNL
P35499	731	LFGKSYKECVCKIALDCNLPR
P35499	737	KECVCKIALDCNLPRWHMHDF
P35499	1189	VNLFAGKFYYCINTTTSERFD
P35499	1400	MIFIIIFTGECVLKMLALRQY
P35499	1528	NFETFGNSIICLFEITTSAGW
P35499	1553	NPILNSGPPDCDPNLENPGTS

P35499	1730	TTLKRKHEEVCAIKIQRAYRR
P36873	105	DRGKQSLETICLLLAYKIKYP
P36873	172	AAIVDEKIFCCHGGLSPDLQS
P36873	202	PTDVPDQGLLCDLLWSDPDKD
P36873	245	FLHKHDLDLICRAHQVVEDGY
P38117	42	DGVKHSMNPFCEIAVEEAVRL
P38117	66	KLVKEVIAVSCGPAQCQETIR
P38117	131	LLGKQAIDDDCNQTGQMTAGF
P38646	66	VGIDLGTTNSCVAVMEGKQAK
P38646	487	DGQTQVEIKVCQGEREMAGDN
P38646	608	EFKDQLPADECNKLKEEISKM
P40925	154	APSIPKENFSCLTRLDHNRAK
P40926	212	GKTIIPLISQCTPKVDFPQDQ
P40939	97	SAVLISSKPGCFIAGADINML
P40939	145	KPIVAAINGSCLGGGLEVAIS
P40939	156	LGGGLEVAISCQYRIATKDRK
P40939	747	EAAYGKQFTPCQLLADHANSP
P42345	216	REGAVAALRACLILTTQREPK
P42345	300	TQQQLVHDKYCKDLMGFGTKP
P42345	362	AKSTLVESRCCRDLMEEKFDQ
P42345	374	DLMEEKFDQVCQWVLKCRNSK
P42345	423	LQDTMNHVLSCVKKEKERTAA
P42345	713	QVFEIRELAICTVGRLSSMNP
P42345	1004	MPTFLNVIRVCDGAIREFLFQ
P42345	1750	KQELHKLMARCFLKLGEWQLN
P42345	1999	NAANKILKNMCEHSNTLVQQA
P42345	2243	NSGLIGWVPHCDTLHALIRDY
P42345	2361	GKILHIDFGDCFEVAMTREKF
P42357	58	VDDAHFLVRRCKGLGLLDNED
P42357	241	KQVIEMFNASCLPYVPEKGTV
P42357	317	NGTQMITSLGCEAVERASAIA
P42357	475	AISERRIERLCNPSLSELPAF
P42357	561	QVLAIELLAACQGIEFLRPLK
P43155	337	LQFIVAEDGSCGLVYEHAAAE
P43155	577	GPVVPDGYGVCYNPMEAHINF
P45880	76	TGTLETKYKWCEYGLTFTEKW
P45880	103	GTEIAIEDQICQGLKLTFDTT
P45880	138	SYKRECINLGCDVDFDFAGPA
P48047	15	AVSGLSRQVRCFSTSVVRPFA
P48047	141	MMSVHRGEVPCTVTSASPLEE
P49189	45	ATGRVIATFTCSGEKEVNLAV
P49189	173	ASWKSAPALACGNAMVFKPSP
P49189	289	ANFLTQGQVCCNGTRVFVQKE
P49189	355	VAKEQGAKVLCGGDIYVPEDP
P49189	376	KLKDGYYMRPCVLTNCRDDMT
P49189	443	RVVAELQAGTCFINNYNVSPV
P49327	180	NAYQAIHSGQCPAAIVGGINV
P49327	212	RLGMLSPEGTCKAFDTAGNGY
P49327	779	AVLKRGLKPSCTIIPLMKKDH
P49327	1118	EQQVPILEKFCFTPHTEEGCL
P49327	1127	FCFTPHTEEGCLSERAALQEE
P49327	1693	AAIAIALSLGCRVFTTVGSAE
P49327	1759	AEEKLQASVRCLATHGRFLEI
P49327	1828	IRDGVVRPLKCTVFHGAQVED
P49327	1881	KLMSAISKTFCPAHKSYIIAG

P49327	1992	NQTPEFFQDVCKPKYSGTLNL
P49327	2202	SKADEASELACPTPKEDGLAQ
P49454	76	KRENQRLMEICESLEKTKQKI
P49454	383	CQRQNAESARCSLEQKIKEKE
P49454	503	SHSEQKAREVCHLEAELKNIK
P49454	515	LEAELKNIKQCLNQSQNFAEE
P49454	569	AVADLEKQRDCSQDLLKKREH
P49454	621	ELKEEKTLFSCWKSENEKLLT
P49454	650	LQSKINHLETCLKTQQIKSHE
P49454	722	QKHQKEIENMCLKTSQLTGQV
P49454	1055	AAQEKNSKLECLLNECTSLCE
P49454	1188	VKERESERNQCNFKPQMDLEV
P49454	1264	QSQEISGLKDCEIDAEEKYIS
P49454	1291	TSQNDNAHLQCSLQTTMNKLN
P49454	1307	MNKLNELEKICEILQAEKYEL
P49454	1521	NLEGAVSANQCSVDEVFCSSL
P49454	1528	ANQCSVDEVFCSSLQTYVDSL
P49454	1617	NLEGVVSANQCSVDEVFCSSL
P49454	1624	ANQCSVDEVFCSSLQEENLTR
P49454	1693	LLSSERQELDCLRKQYLSENE
P49454	1767	EDAIQGRNESCDISKEHTSET
P49454	1790	RTPKHDVHQICDKDAQQDLNL
P49454	1947	DLSEKLEYFSCDHQELLQRVE
P49454	2030	DLEVVQTEKLCLEKDNENKQK
P49454	2148	KDSQALSLTKCELENQIAQLN
P49454	2415	RLEADEKKQLCVLQQLKESEH
P49454	2560	VAALHNDQEACKAKEQNLSSQ
P49454	2776	ENSELKKSLDCMHKDQVEKEG
P49454	2834	YREKLTSKEECLSSQKLEIDL
P49454	2898	QGKMKLLIKSCKQLEEEKEIL
P50851	94	FIIQEGESINCMVDLLEKCDI
P50851	256	NVDKDKPYLYCFRTSKGLGYS
P50851	273	LGYSAHFVGGCLIVTSIKSKG
P50851	289	IKSKGKGFQHCVKFDFKPQKW
P50851	318	YNRWKNSELRCYVNGELASYG
P50851	343	FVNTSDTFDKCFLGSSETADA
P50851	498	YLSDEIDLTICSTLLAFIMEL
P50851	548	SHVSRAVLELCLAFSKYLSNL
P50851	950	QGKVDEEIGLCSSTSVQAASG
P50851	982	QQPDTKDSPVCPHFTTNGNEN
P50851	1458	CAVAVRNCLECQQHSQLKTRG
P50851	1704	TVDPALLPPACLGALGDLSVE
P50851	1935	SADKREDEKMCDHLIRAAKYR
P50851	2017	ATLKTAVEHVCIFKLRENSKA
P50851	2643	VVFGHWDVVTCLARSESYIGG
P50851	2740	LLRTLEGPENCLKPKLIQASR
P50851	2820	DLKQLFAYPGCDAGIRAMALS
P53814	541	SHAPPSSRGGCSIKMEAEPAE
P53814	809	NSIKQMLLDWCRAKTRGYEHV
P54652	191	AIAYGLDKKGCAGGEKNVLIF
P54652	577	EQDKNKILDKCQEVINWLDRN
P54652	606	EHKQKELERVCNPIISKLYQG
P55072	69	LKGKKRREAVCIVLSDDTCSD
P55072	105	RLGDVISIQPCPDVKYGKRIH
P55072	209	NEVGYDDIGGCRKQLAQIKEM

P55072	522	KGVLFYGPPGCGKTLLAKAIA
P55072	535	TLLAKAIANECQANFISIKGP
P55072	572	IFDKARQAAPCVLFFDELDSI
P55072	695	ADLTEICQRACKLAIRESIES
P60174	79	AKVPADTEVVCAPPTAYIDFA
P60174	164	ALAEGLGVIACIGEKLDEREA
P60709	217	EIVRDIKEKLCYVALDFEQEM
P60709	257	VITIGNERFRCPEALFQPSFL
P60900	47	LTSVAVRGKDCAVIVTQKKVP
P60900	161	VYKCDPAGYYCGFKATAAGVK
P60900	201	FEQTVETAITCLSTVLSIDFK
P61163	34	GFAGDQIPKYCFPNYVGRPKH
P61163	222	EIVKAIKERACYLSINPQKDE
P61981	194	YEIQNAPEQACHLAKTAFDDA
P62191	58	KLPLVTPHTQCRLKLLKLERI
P62191	148	FVDKDLLEPGCSVLLNHKVHA
P62736	219	EIVRDIKEKLCYVALDFENEM
P62736	259	VITIGNERFRCPETLFQPSFI
P62736	287	HETTYNSIMKCDIDIRKDLYA
P62873	121	YVACGGLDNICSIYNLKTREG
P62873	204	PDTRLFVSGACDASAKLWDVR
P62873	271	LMTYSHDNIICGITSVSFSKS
P62873	294	LLLAGYDDFNCNVWDALKADR
P62873	317	VLAGHDNRVSCLGVTDDGMAV
P63104	25	QAERYDDMAACMKSVTEQGAE
P63104	189	YEILNSPEKACSLAKTAFDEA
P63261	217	EIVRDIKEKLCYVALDFEQEM
P63261	257	VITIGNERFRCPEALFQPSFL
P68363	129	LDRIRKLADQCTGLQGFLVFH
P68363	347	TKRSIQFVDWCPTGFKVGINY
P68363	376	GDLAKVQRAVCMLSNTTAIAE
P68371	12	REIVHLQAGQCGNQIGAKFWE
P68371	129	VVRKEAESCDCLQGFQLTHSL
P68371	303	MFDAKNMMAACDPRHGRYLTV
P68371	354	WIPNNVKTAVCDIPPRGLKMS
P78527	90	ECREEILKFLCIFLEKMGQKI
P78527	111	APYSVEIKNTCTSVYTKDRAA
P78527	123	SVYTKDRAAKCKIPALDLLIK
P78527	223	REPKLPVLAGCLKGLSSLLCN
P78527	232	GCLKGLSSLLCNFTKSMEEDP
P78527	301	VSLFEVLLKWCAHTNVELKKA
P78527	457	PQYSPKMQLVCCRAIVKVFLA
P78527	478	LAAKGPVLRNCISTVVHQGLI
P78527	491	TVVHQGLIRICSKPVVLPKGP
P78527	729	KQYKDELLASCLTFLLSLPHN
P78527	795	MQPYYKDILPCLDGYLKTSAL
P78527	974	RTFPVLLRLACDVDQVTRQLY
P78527	1127	DEKSLGTIQQCCDAIDHLCRI
P78527	1135	QQCCDAIDHLCRIIEKKHVSL
P78527	1266	WLDLLLAALECYNTFIGERTV
P78527	1499	KGIAPGDERQCLPSLDLSCKQ
P78527	1742	GTPRFNNYVDCMKKFLDALEL
P78527	1791	SFRRIARRGSCVTQVGLLESV
P78527	1919	NELTKTLIKLCYDAFTENMAG
P78527	1953	AAYNCAISVICCVFNELKFYQ

P78527	2093	LEMDELNRHECMAPLTALVKH
P78527	2244	NLEIIKTLVECWKDCLSIPYR
P78527	2292	ANDLPPYDPQCGIQSSEYFQA
P78527	2342	ERKNILEESLCELVAKQLKQH
P78527	2363	QNTMEDKFIVCLNKVTKSFPP
P78527	2435	RHRDDERQKVCLDIIYKMMPK
P78527	3187	NIWDDIITNRCFFLSKIEEKL
P78527	3347	IANALSSEPACLAEIEEDKAR
P78527	3492	LMTKEISSVPCWQFISWISHM
P78527	3781	MNGILAQDSACSQRALQLRTY
P78527	3837	AYLSDPRAPPCEYKDWLTKMS
P78527	4045	EKNWYPRQKICYAKRKLAGAN
P78559	71	LVDGGSDRKSCFWKLVRHLDR
P78559	912	EEDKGFKSPPCEDFSVTGESE
P78559	1086	PVNIDEGLTGCTIQLLPAQDK
P78559	1174	ANQEPTPKSPCGLTEQYLHKD
P78559	1702	EQDTYWRELSCERKVWFPHEL
P78559	1999	PLGAAGDWPPCLSTKEAAAGR
P78559	2339	GDGILPCHLECSEAATEKPSP
P78559	2492	VGGPGTTGGPCPVTDETPPTS
P78559	2702	YVDLAYIPNHCSGKTADLDFF
Q01813	98	VGGTIIGSARCQAFRTREGRL
Q01813	232	GYLALVSALACGADWVFLPES
Q01813	718	GKKFTTDDSICVLGISKRNVI
Q01955	41	CVCKDKGQCFCDGAKGEKGEK
Q01955	125	GPYGLVGVPGCSGSKGEQGFP
Q01955	422	DAMGTPGSPGCAGSPGLPGSP
Q01955	722	GFPGTKGSLGCPGKMGEPGLP
Q01955	1387	PPGPPGNLGPCGPRGKPGKDG
Q01955	1493	HGQDLGTLGSCLQRFTTMPFL
Q01955	1551	LEPYISRCTVCEGPAIAIAVH
Q01955	1570	VHSQTTDIPPCPHGWISLWKG
Q01955	1616	EEFRASPFLECHGRGTCNYYS
Q02952	684	DTSVSWEALICVGSSKKRARR
Q02952	1399	VSSLEGSPPPCLGQEEAVCTK
Q02952	1407	PPCLGQEEAVCTKIQVQSSEA
Q02952	1479	GEDAVPTGPDCQAKSTPVIVS
Q06830	52	FFYPLDFTFVCPTEIIAFSDR
Q06830	83	VIGASVDSHFCHLAWVNTPKK
Q06830	173	FQFTDKHGEVCPAGWKPGSDT
Q12931	35	AAVPGGKPILCPRRTTAQLGP
Q12931	501	RAGTRNIYYLCAPNRHLAEHS
Q12931	527	MKKKDTEVLFCFEQFDELTLL
Q12955	70	YIKNGVDINICNQNGLNALHL
Q12955	307	KTRDGLTPLHCGARSGHEQVV
Q12955	377	YLTALHVAAHCGHYKVAKVLL
Q12955	738	KMGYTPLHVGCHYGNIKIVNF
Q12955	1014	GMRIIIPPRKCTAPTRITCRL
Q12955	1022	RKCTAPTRITCRLVKRHKLAN
Q12955	1123	SPEELGKKRICRIITKDFPQY
Q12955	1369	VLEGKPIYVDCYGNLAPLTKG
Q12955	1430	TTKGLPQTAVCNLNITLPAHK
Q12955	1735	YPSSSTLINGCKATATLQEKI
Q12955	1967	KVSEILKKDVCVDNKGSPKSP
Q12955	2835	FSEQQAKDLACHITSDLATRG

Q12955	3035	VSKHQSYVGLCPPLEETETSP
Q12955	3122	GGVKKIISQECKTVQETRGTF
Q12955	3395	NGNNDQSITECSIATTAEFSH
Q12955	3752	TDKIEAVMTSCQGLENETITM
Q12955	3816	IVLTEHSAPTCTTEKDNPVKV
Q12955	3906	EKTKALTTSSCVDVKSRIPVK
Q12955	3930	RDNIIAVRKACATQKQGQPEK
Q12955	3958	SKLPVKVRSTCVTTTTTTATT
Q12955	3980	TTTTTTTTSCTVKVRKSQLK
Q12955	3993	KVRKSQLKEVCKHSIEYFKGI
Q12955	4321	SKLIIEETKPCVPVSMKKMSR
Q13011	92	NKVFWREMVECFNKISRDADC
Q13011	159	YQETFNVIERCPKPVIAAVHG
Q13011	171	KPVIAAVHGGCIGGGVDLVTA
Q13011	187	DLVTACDIRYCAQDAFFQVKE
Q13136	130	NTRLLLEHLECLVSRHERSLR
Q13136	1019	VDSFHRNSFQCGIMCLRRLNY
Q13283	73	RKVMSQNFTNCHTKIRHVDAH
Q13509	12	REIVHIQAGQCGNQIGAKFWE
Q13509	129	VVRKECENCDCLQGFQLTHSL
Q13509	303	MFDAKNMMAACDPRHGRYLTV
Q13509	354	WIPNNVKVAVCDIPPRGLKMS
Q13535	116	RLLRIAATPSCHLLHKKICEV
Q13535	128	LLHKKICEVICSLLFLFKSKS
Q13535	347	LKSDLLKAALCHLLQYFLKFV
Q13535	376	QVRKVYVRNICKALLDVLGIE
Q13535	520	QNMNCRTFKDCQHKSKKKPSV
Q13535	607	PWIYSHSDDGCLKLTTFAANL
Q13535	763	CSSSQLKASVCKPFLFLLKKK
Q13535	895	VPFALLHLLHCLLSKSASVSG
Q13535	1042	NFKYIFSHLVCSCSKDELERA
Q13535	1193	RFKDDFPELCCRAWDCFVRCL
Q13535	1514	HDLASKIFTCCSIMMKHDFKV
Q13535	1604	HKFQALKAEKCPHSKSNRNKV
Q13535	2031	IMKKYKDVTACLPEWEDGHFY
Q13535	2409	VYMTGKELRQCMLPKSAALSE
Q13535	2497	TGECVHVDFNCLFNKGETFEV
Q13576	42	RRQNIAYEYLCHLEEAKRWME
Q13576	548	WVTLVVDVNQCLEGKKSSDIL
Q13576	575	TSNANDIIPECADKYYDALVK
Q13576	1183	QEFRKYFKEACNVPEPEEKFN
Q13576	1449	INYYDTYIKTCLDNLKRKNTR
Q13618	251	INEEIERVMHCLDKSTEEPIV
Q13618	462	KNMISKLKTECGCQFTSKLEG
Q13618	464	MISKLKTECGCQFTSKLEGMF
Q13618	522	YWPTQSATPKCNIPPAPRHAF
Q13618	636	ELVRALQSLACGKPTQRVLTK
Q13748	129	LDRIRKLADLCTGLQGFLIFH
Q13748	347	TKRTIQFVDWCPTGFKVGINY
Q13748	376	GDLAKVQRAVCMLSNTTAIAE
Q13885	12	REIVHIQAGQCGNQIGAKFWE
Q13885	129	VVRKESESCDCLQGFQLTHSL
Q13885	354	WIPNNVKTAVCDIPPRGLKMS
Q13939	120	YFNTPRLRVHCNDFLIKSICR
Q13939	266	VLMDRKQERPCSLLVYQRKGA

Q13939	404	YRYDERKEVWCLAGKMSIPMD
Q13939	433	DRHLYIVTGRCLVKGYISRVG
Q13939	530	CSIGDSKVFVCGGVTTASDVQ
Q13939	575	PPPEALDCPACCLAKLPCKIL
Q13939	576	PPEALDCPACCLAKLPCKILQ
Q14008	16	EWLKLPVDQKCEHKLWKARLS
Q14008	144	DNKNPKIIVACIETLRKALSE
Q14008	427	SLFIARSFRHCTASTLPKSLL
Q14008	441	TLPKSLLKPFCAALLKHINDS
Q14008	493	DKLKLDKIKECSEKVELIHGK
Q14008	695	GLVDKIGDVKCGNNAKEAMTA
Q14008	1113	FQPASAPAEDCISSSTEPKPD
Q14008	1241	LESEKEGVIGCLDLILKWLTL
Q14008	1360	CLVESYGMNVCQPTPGKALKE
Q14008	1616	EKDEIIKLYSCIIGNMISLFQ
Q14008	1768	RTLKTLLHTLCKLKGPKILDH
Q14008	1946	LERLKILRQRCGLDNTKQDDR
Q14315	46	KIQQNTFTRWCNEHLKCVGKR
Q14315	52	FTRWCNEHLKCVGKRLTDLQR
Q14315	438	LEDKGDSTFRCTYRPAMEGPH
Q14315	665	IAHILPAPPDCFPDKVKAFGP
Q14315	682	AFGPGLEPTGCIVDKPAEFTI
Q14315	712	LKLYAQDADGCPIDIKVIPNG
Q14315	728	VIPNGDGTFRCSYVPTKPIKH
Q14315	1066	GVLPPDPSKVCAYGPGLKGGL
Q14315	1103	GGLGLTVEGPCEAKIECQDNG
Q14315	1348	SPFRVGVTEGCDPTRVRAFGP
Q14315	1448	KDVVDPGKVKCSGPGLGAGVR
Q14315	1653	VSIGGHGLGACLGPRIQIGQE
Q14315	1680	AKAAGEGKVTCTVSTPDGAEL
Q14315	1914	ITCKDNKDGTCTVSYLPTAPG
Q14315	1991	IRAPSGNEEPCLLKRLPNRHI
Q14315	2154	APSIATIGSTCDLNLKIPGNW
Q14315	2660	VGQKNSFTVDCSKAGTNMMMV
Q14524	182	SLVKILARGFCLHAFTFLRDP
Q14524	597	LHGKKNSTVDCNGVVSLLGAG
Q14524	726	DPFTDLTITMCIVLNTLFMAL
Q14524	981	RFVKRTTWDFCCGLLRQRPQK
Q14524	982	FVKRTTWDFCCGLLRQRPQKP
Q14524	1004	ALAAQGQLPSCIATPYSPPPP
Q14524	1128	QWKAEPQAPGCGETPEDSCSE
Q14524	1179	TEGCVRRCPCCAVDTTQAPGK
Q14524	1384	NYTIVNNKSQCESLNLTGELY
Q14524	1575	LLFVAIFTGECIVKLAALRHY
Q14524	1703	NFQTFANSMLCLFQITTSAGW
Q14566	91	EEFYRVYPYLCRALKTFVKDR
Q14566	185	TQPNICRNPVCANRRRFLLDT
Q14566	301	DLSYRLVFLACCVAPTNPRFG
Q14566	351	SQDKNLYHNLCTSLFPTIHGN
Q14566	457	GALMLADNGVCCIDEFDKMDV
Q14990	68	YSKRSRSCGLCDLYPCCLCDY
Q14990	121	TTNRILASSCCSSNILGSVNV
Q14990	149	VKVRVKDGKVCVSAERENRYD
Q14990	160	VSAERENRYDCLGSKKYSYMN
Q14990	172	GSKKYSYMNICKEFSLPPCVD

Q14990	180	NICKEFSLPPCVDEKDVTYSY
Q15149	530	QLRSEFERLECLQRIVTKLQM
Q15149	730	SPATRGAYRDCLGRLDLQYAK
Q15149	849	TQWSWMLQLCCCIEAHLKENA
Q15149	888	LQEALRRKYSCDRSATVTRLE
Q15149	950	MRGRLPLLAVCDYKQVEVTVH
Q15149	1156	LPLDKEPARECAQRIAEQQKA
Q15149	3017	RCVEDPETGLCLLPLTDKAAK
Q15149	3299	VPLDVACARGCLDEETSRALS
Q15149	3493	SVRTLLQGSGCLAGIYLEDTK
Q15149	4071	NLQKFLEGTSCIAGVFVDATK
Q15149	4254	RCITDPQTGLCLLPLKEKKRE
Q15149	4454	TGQRLLEAQACTGGIIDPSTG
Q15149	4574	DVGAYSKYLTCPKTKLKISYK
Q16555	179	AFKDRFQLTDCQIYEVLSVIR
Q16555	248	RAITIANQTNCPLYITKVMSK
Q16555	504	VPRGLYDGPVCEVSVTPKTVT
Q16658	19	AVQIQFGLINCGNKYLTAEAF
Q16658	61	PPDEAGSAAVCLRSHLGRYLA
Q16658	121	YFGGTEDRLSCFAQTVSPAEK
Q16658	226	FRSGKVAFRDCEGRYLAPSGP
Q16658	305	QLEIDRDTKKCAFRTHTGKYW
Q16658	456	DTPVDFFFEFCDYNKVAIKVG
Q3LXA3	24	ADDALAGLVACNPNLQLLQGH
Q3LXA3	155	VLKKAGRRGLCGTVLIHKVAG
Q3LXA3	404	ALDRAAGDGDCGTTHSRAARA
Q460N5	42	QSPKRSGGGECEVRQDPRSPS
Q460N5	208	HIDTIRFVDDCTKHHSIKQLQ
Q460N5	522	GPSADVYKAKCEIQEKVYTMA
Q460N5	650	ETTAEVIITGCVKEVNETYKL
Q460N5	739	DIVKQVWDSVCVKSVHTDKPG
Q460N5	771	YQSEIKRLFGCYIELQENEVM
Q460N5	792	KEGGSPAGQKCFSRTVLAPGV
Q460N5	906	LLRRAVQLSLCLAEKYKYRSI
Q460N5	933	SGVFGFPLGRCVETIVSAIKE
Q460N5	953	ENFQFKKDGHCLKEIYLVDVS
Q460N5	1265	KAGVSKAILECAGQNVERECS
Q460N5	1295	YIITGGGFLRCKNIIHVIGGN
Q460N5	1327	ECEKKNYSSICLPAIGTGNAK
Q460N5	1455	WLQDLIEKEQCPYTSEDECIK
Q460N5	1547	WQYNDNNTSHCFNKMTNLKLE
Q460N5	1640	NTVASKFNQTCSHFRIEKIER
P15313	45	THPRVTYRTVCSVNGPLVVLD
Q5JQC9	516	TIWNQKQGNSCKVATKACSNK
Q5JQC9	523	GNSCKVATKACSNKDEKGEKI
Q5JQC9	587	YMAQSTQYEKCGGGQSAKALS
Q5JQC9	649	NENPFKCEDPCEGENKCSEPR
Q5JQC9	685	EQCQEHQELDCTSGMKQANGQ
Q5JQC9	765	LGHEVIVNNQCSTNSLQKQLQ
Q5JRA6	124	QVPTDETDFVCFDGGRDDFHN
Q5JRA6	1417	NCITQLNLLECESESEGQNKG
Q5JRA6	1486	LKLRASVSTKCNLEDQVKKLE
Q5JRA6	1513	QAAKAGLEDECKTLRQKVEIL
Q5JRA6	1677	FGPSPVSGGECSPPLTVEPPV
Q5TZA2	28	VIQTLESSVLCQEKGLGARDL

Q5TZA2	187	QGKILQYKKRCSELEQQLLER
Q5TZA2	515	RSPRRGPSPACSDSSTLALIH
Q5TZA2	1759	NLHLQKALTACEHDRQVLQER
Q5VST9	247	ASPPSTGTRTCTVTEGKHARL
Q5VST9	259	VTEGKHARLSCYVTGEPKPET
Q5VST9	354	VREKESATFLCEVPQPSTEAA
Q5VST9	404	VSADDDAVYICETPEGSRTVA
Q5VST9	661	LKASSVYEIHCDRTRHTLTIR
Q5VST9	962	AGQADAGEYSCEAGGQRLSFH
Q5VST9	1054	AGKTDAGDYSCEARGQRVSFR
Q5VST9	1330	AGQADAGEYTCEAGGQRLSFH
Q5VST9	1413	CTRRLVVQQACQADTGEYSCE
Q5VST9	1514	AGQAVAGEYSCEAGSQRLSFH
Q5VST9	1606	AGQADAGEYSCKAGDQRLSFH
Q5VST9	1698	AGQAEAGEYSCEAGGQQLSFR
Q5VST9	1843	SPACAEVVWRCGNTQLRVGKR
Q5VST9	1880	LRAEDAGEYVCESRDDHTSAQ
Q5VST9	2059	VGFADRGFFGCETPDDKTQAK
Q5VST9	2187	TTEKEKVTLECELSRPNVDVR
Q5VST9	2219	KTMAIAAQGACRSLTIYRCEF
Q5VST9	2311	AMEKHRGVLECQVSRASAQVR
Q5VST9	2361	VHAEDEDTYTCDAGDVKTSAQ
Q5VST9	2579	ATEEGWASFSCELSHEDEEVE
Q5VST9	2668	AEERGTLALQCEVSDPEAHVV
Q5VST9	2718	VSPEDAGLYTCHVGSEETRAR
Q5VST9	2848	VAPGEDVELRCELSRAGTPVH
Q5VST9	2876	IRKSQKYDVVCEGTMAMLVIR
Q5VST9	2937	VEEKGTAVFTCKTEHPAATVT
Q5VST9	3141	DGKTLRGSARCQLSHEGHRAQ
Q5VST9	3173	GRYKCEAGGACSSSIVRVHAR
Q5VST9	3206	VLEGGAATLRCVLSSVAAPVK
Q5VST9	3218	LSSVAAPVKWCYGNNVLRPGD
Q5VST9	3295	ATEGATATLRCELSKAAPVEW
Q5VST9	3383	SIEGATATLRCELSKAAPVEW
Q5VST9	3471	AVEGATAMLWCELSKVAPVEW
Q5VST9	3559	AREGATAVLQCELNSAAPVEW
Q5VST9	3647	ATEGATAVLRCELSKMAPVEW
Q5VST9	3735	ATEGDTATLWCELSKAAPVEW
Q5VST9	3823	AREGATAVLQCELSKAAPVEW
Q5VST9	3874	VADTGEYSCVCGQERTSATLT
Q5VST9	3942	NGRREPRLQGCTAELVLQDLQ
Q5VST9	4050	VEQEDAGDYTCDTGHTQSMAS
Q5VST9	4180	VTADEDVEFSCEVSRAGATGV
Q5VST9	4328	AGTVSFHVGTCSSEAQLKVTA
Q5VST9	4703	YHCGLAQGSICPAAATFQVAL
Q5VST9	4919	AQVGDALRLECVVASKADVRA
Q5VST9	4971	LDRADAGCYTCQVSNKFGQVT
Q5VST9	5059	QTYREDEHFICIRFEALTEAR
Q5VST9	5101	VEQGPRRVEMCISKETPAPVV
Q5VST9	5449	VGRQHQGTYTCIASNAAGQAL
Q5VST9	5770	QCDTDDDVAMCFIKNQAAFEQ
Q5VST9	5857	IRNKARNRQNCALLEQAYAVV
Q5VST9	5932	VFLFRNHLVICKPRRDSRTDT
Q5VST9	6035	AELGETVKLACRVTGTPKPVI
Q5VST9	6429	VASKDAGVYTCLAQNTGGQVL

Q5VST9	6494	RVQHKGNKILCAAKFIPLRSR
Q5VST9	7035	DSPWGQPAPFCHPKQGSAPQE
Q5VST9	7484	VVLGQSVTLACQVSAQPAAQA
Q5VST9	7610	TTLASDIFDCCYLTSKLSRGG
Q5VST9	7628	RGGTYTFRTACVSKAGMGPYS
Q6IA69	169	GDAVLVTWDTCIGSEICEELW
Q6IA69	235	GIYLLANQKGCDGDRLYYDGC
Q6IA69	309	PRVKVDFALSCHEDLLAPISE
Q6IA69	428	MASKNSSQETCTRARELAQQI
Q6IA69	554	KTDLRAFVQFCIQRFQLPALQ
Q6IA69	627	CKLLGMWRHICTPRQVADKVK
Q6IA69	686	YNTSWPWQFRCIENQVLQLER
Q6JEL2	26	HMERKMSAMACEIFNELRLEG
Q6JEL2	39	FNELRLEGKLCDVVIKVNGFE
Q6JEL2	58	FEFSAHKNILCSCSSYFRALF
Q6JEL2	128	QFNIMGIVRGCCEFLKSELCL
Q6JEL2	129	FNIMGIVRGCCEFLKSELCLD
Q6JEL2	141	FLKSELCLDNCIGICKFTDYY
Q6JEL2	153	GICKFTDYYYCPELRQKAYMF
Q6JEL2	255	MNDYVKDSEECKPVIINALKA
Q6JEL2	377	HQVAPMHSRRCYVSVTVLGNF
Q6JEL2	438	ATTLYGKVYICGGFNGNECLF
Q6JEL2	446	YICGGFNGNECLFTAEVYNTE
Q6JEL2	546	NGFTTTFNVECYDEKTDEWYD
Q6JEL2	569	DMSIYRSALSCCVVPGLANVE
Q6PKC3	67	LMARQRPELLCGAVALGCALL
Q6PKC3	137	VVLLFFYAPWCGQSIAARAEI
Q6PKC3	172	AINCWWNQGKCRKQKHFFYFP
Q6PKC3	450	PDPPTITASPCCNTVVLPQWH
Q6PKC3	601	MTSTNFTGLSCRTNKTLNIYL
Q6PKC3	770	DRLPTVLFFPCNRKDLSVKYP
Q6UB99	501	DDRDSLGSSGCLKGSPLVLKD
Q6UB99	646	HKHKNKEKGQCSISQELKLKS
Q6UB99	818	YREDSAFDEYCNKNQFLENED
Q6UB99	958	GRDRKDALESCKERRDGRAKP
Q6UB99	981	AHREELKECGCESGFKDKSDG
Q6UB99	1046	DKSEKSILEKCQKDKEFDKCF
Q6UB99	1055	KCQKDKEFDKCFKEKKDTKEK
Q6UB99	1339	PGDDKPRESACLPEKLKEKER
Q6UB99	1712	GVPTPTSVLSCPSYEEVMHTP
Q6UB99	1845	LPPVPAEKFACLSPGYYSPDY
Q6UB99	1867	LPSPKVDALHCPPAAVVTVTP
Q6UB99	2456	KKIEEKRKILCCITPQAPQCY
Q6UB99	2457	KIEEKRKILCCITPQAPQCYA
Q6UB99	2530	SIEREKLIVSCEQEILRVHCR
Q6UB99	2539	SCEQEILRVHCRAARTIANQA
Q6UB99	2604	VDDKYDRMKTCLLMRQQHEAA
Q6UVJ0	156	DVEIKKFLAGCLKCSKEEKLS
Q6UVJ0	310	RRENSTLDVECHEKEKHVNQL
Q6UVJ0	444	QSLRIKEQEVCKLQEQLEATV
Q6UVJ0	525	VVDGRLTYPTCGIGYPVSSAF
Q6ZMR3	35	IVGTGSVGVACAISILLKGLS
Q6ZMR3	131	IPNITQYSPHCKLLIVTNPVD
Q6ZMR3	315	KVKLTLEEEACLQKSAETLWE
Q71U36	129	LDRIRKLADQCTGLQGFLVFH

Q71U36	347	TKRTIQFVDWCPTGFKVGINY
Q71U36	376	GDLAKVQRAVCMLSNTTAIAE
Q86UR5	56	EEEKEEAMLKCVVRDMAKPAA
Q86UR5	67	VVRDMAKPAACKTPRNAENQP
Q86UR5	116	QGEHKDDAPTCGICHKTKFAD
Q86UR5	119	HKDDAPTCGICHKTKFADGCG
Q86UR5	143	SYCRTKFCARCGGRVSLRSNN
Q86UR5	162	NNEDKVVMWVCNLCRKQQEIL
Q86UR5	1034	PRAKRGRSAECLHTTRHLVRH
Q86UR5	1190	ASDAERVLPTCLSRRGHAAPR
Q86UR5	1332	YNIHKDQYRSCDNVSAKSSDS
Q86UR5	1587	VKVYLLENGACIAKKKTRIAR
Q86YZ3	94	ARNKIIGKDYCQVSGSKLRDD
Q86YZ3	195	DSHQSSGRGQCGSGSGQSPNY
Q8IWV7	86	WYLFGEDPDICLEKLKHSGAF
Q8IWV7	149	KMHTSTGGGFCDCGDTEAWKT
Q8IWV7	180	RAGTIKENSRCPLNEEVIVQA
Q8IWV7	228	QIREKNERYYCVLFNDEHHSY
Q8IWV7	279	RAVKAGAYAACQEAKEDIKSH
Q8IWV7	350	LREEPDSENPCLISRLMLWDA
Q8IWV7	477	DKLGRVYAVICDLKYILISKP
Q8IWV7	777	VTMREIIHLLCIEPMPHSAIA
Q8IWV7	884	AFSKVINLLNCDIMMYILRTV
Q8IWV7	993	DTVKRLREKSCLIVATTSGSE
Q8IWV7	1101	TEKEVLTCILCQEEQEVKIEN
Q8IWV7	1369	MQFAVAQRITCPQVLIQKHLV
Q8IWV7	1396	LPNIKSEDTPCLLSIDLFHVL
Q8IWV7	1569	DTVRPLLQRWCADPALLNCLK
Q8IWV7	1673	GVCIFLKIRECRVVLVEGKAR
Q8IWV7	1685	VVLVEGKARGCAYPAPYLDEY
Q8IWV7	1726	RKLHLVWQQHCIIEEIARSQE
Q8N1C8	68	VGIDLGTTNSCVAVMEGKRAK
Q8N1C8	489	DGQTQVEIKVCQGEREMAGDN
Q8N1C8	610	EFKDQLPADECNKLKEEISKM
Q8N427	287	YLERQHLAQLCDIEEDAANVA
Q8NBX0	77	TLSSEVGIIICDIANPASLDE
Q8NBX0	98	MAKQATVVLNCVGPYRFYGEP
Q8NBX0	361	TDKNKPNIKICTQVKGPEAGY
Q8NDH3	170	NGPVEVSTLQCLANATDGVRL
Q8NDH3	189	RLAARIVDTPCNEMNTDTFLE
Q8NDH3	311	GFKDNLHAVFCLAENSVGPNA
Q8NDX6	103	FQVKIPKNFVCEHCFGAFRSS
Q8NDX6	106	KIPKNFVCEHCFGAFRSSYHL
Q8NDX6	134	TGEKPFECDICDMRFIQKYHL
Q8NDX6	165	KPYQCERCHQCFSRTDRLLRH
Q8NEB7	60	LTPTWKAETTCRLRATHGCRN
Q8NEB7	68	TTCRLRATHGCRNPTLVQLDQ
Q8NEB7	123	NHVYYAKRVLCSQPVSILSPN
Q8NEB7	337	VLCYSIVENTCIITPTAKAWK
Q8NEB7	477	FQDGDFPTKICDTDYIQYPNY
Q8NEB7	488	DTDYIQYPNYCSFKSQQCLMR
Q8NEB7	509	NRNRKVSRMRCLQNETYSALS
Q8NEZ4	208	ERSPQQNIVSCVSVSTQTASD
Q8NEZ4	298	CKHLGATIKCCEEKCTQMYHY
Q8NEZ4	327	FQDFSHIFLLCPEHIDQAPER

Q8NEZ4	347	RSKEDANCAVCDSPGDLLDQF
Q8NEZ4	473	QDNLCPFCGKCYHPELQKDML
Q8NEZ4	496	NMCKRWVHLECDKPTDHELDT
Q8NEZ4	988	SQCGQCYHPYCVSIKITKVVL
Q8NEZ4	1244	DDSREGELMDCDGKSESSPER
Q8NEZ4	1953	ANRPSPVRDLCSSSTTNNDPY
Q8NEZ4	2789	LPIDDKLDNQCVSVEPKKKEQ
Q8NEZ4	2853	DENKDNVDTPCSQASAHSDLN
Q8NEZ4	2873	NDGEKTSLHPCDPDLFEKRTN
Q8NEZ4	3728	IKLEKAETESCPGQEEPKLEE
Q8NEZ4	3751	GSKVEGNAVACPVSSAQSPPH
Q8NEZ4	3804	PEGSICSEDDCTKDNKLVEKQ
Q8NEZ4	3992	NQEELRIQDHCGDRDTPDSFV
Q8NEZ4	4201	AALRPQWCCHCKVVILGSGVR
Q8NEZ4	4291	SNNISTLDVHCLPQLPEKASP
Q8NEZ4	4375	VIPKGTFKPPCEDEIDEFLKK
Q8NEZ4	4432	LDLDLWVHLNCALWSTEVYET
Q8NEZ4	4487	FRCTNIYHFTCAIKAQCMFFK
Q8NEZ4	4643	VWDKILEPVACVRKKSEMLQL
Q8NEZ4	4901	EDDQHKIPCHCGAVNCRKWMN
Q8TAA3	72	RKICALDDHVCMAFAVLTIFI
Q8TAA3	99	RVVINRARVECQSHKLTVEDP
Q8TD31	765	SEAISKEEAVCQGDNLDRCSS
Q8TDR2	95	GGKRAARKWRCAGQVTIQGPA
Q8TDR2	235	GARVAVKKIRCDAPENVELAL
Q8TDR2	388	KVADFGLSKVCAGLAPRGKEG
Q8TDR2	416	NVNKYWLSSACGSDFYMAPEV
Q8TDY3	165	LVVDSGDAVTCTVPIFEGYSL
Q8TDY3	206	LLLASGHTFPCQLDKGLVDDI
Q8TDY3	221	GLVDDIKKKLCYVALEPEKEL
Q8TE82	345	LGAQVPSLPWCVGRHAASGRV
Q8TE82	390	EEEKSFFSEGCFSEEDARQLL
Q8TE82	456	VKNVLEQCKTCPGCPQEPASW
Q8TE82	721	LLLADIYSRKCLPHLVLSCVK
Q8TE82	801	SLAQLYSHHGCHGPAITFMTQ
Q8TE82	1006	YSAVMPSEAQCVIYHELQLSL
Q8TE82	1155	TGNRKAELRLCNKLVALLATL
Q8TE82	1222	EHFYLKALSLCNSPLEFDEET
Q8WWI5	26	REWKPLEDRSCTDIPWLLLFI
Q8WWI5	69	VSGYDSYGNICGQKNTKLEAI
Q8WWI5	98	QRKYVFFLDPCNLDLINRKIK
Q8WWI5	113	INRKIKSVALCVAACPRQELK
Q8WWI5	159	YTTSPKSSVLCPKLPVPASAP
Q8WWI5	183	FHRCAPVNISCYAKFAEALIT
Q8WWI5	219	MTSKEIILGLCLLSLVLSMIL
Q8WWI5	484	CARCVLKSCICCLWCLEKCLN
Q8WWI5	512	TATAINSTNFCTSAKDAFVIL
Q8WWI5	585	VCLFAFLVAHCFLSIYEMVVD
Q8WWI5	600	YEMVVDVLFLCFAIDTKYNDG
Q8WWK9	466	DAKKLVKYWICLALIEPITSP
Q8WWK9	524	ANLGENMEKSCASKEEVKEVS
Q8WWK9	562	KLHRNLLFQDCEKEQDNKTKD
Q8WWK9	588	KTPNTETRTSCLIKYNVSTTP
Q8WWK9	648	LPDMLKDHYPCVSSLEQLTEL
Q8WWK9	667	ELGRETDAFVCRPNAALCRVY

Q8WXH0	39	QEDTQKKAFTCWINSQLARHT
Q8WXH0	90	PRDKGSNTFQCRINIEHALTF
Q8WXH0	146	HIEKLAQTLSCNYNQPSLDDV
Q8WXH0	587	VKSTLQKVLACWATYVENLRL
Q8WXH0	923	MSLEEKSRDVCAKWESLHHEL
Q8WXH0	1032	YEQKIERLLKCASEIHMTLQP
Q8WXH0	1235	LPVEKASLLLCGSDLPLHKMA
Q8WXH0	1432	FTEENKLLEACIFKNNELLKN
Q8WXH0	1509	DGREKTVNQQCQNTVVLWENT
Q8WXH0	1598	LEVVDKINQVCKNLQFYLNKM
Q8WXH0	1806	SMILLENQIGCLTPELSELKK
Q8WXH0	1861	FSNIKVNLKECFESSETKKSV
Q8WXH0	1931	EFELEKMESICQARAKELEDS
Q8WXH0	1980	EEPGEKTELFCQALARKREQF
Q8WXH0	2337	IIKDDIKSLQCKQKDLENRLA
Q8WXH0	2355	RLASAKQEMECCLNSILKSKR
Q8WXH0	2480	AAIKPLEQTECLNKTETGALV
Q8WXH0	2520	TLKKNKESQYCVLRDFQEYLA
Q8WXH0	2730	HLEAENQIKKCDIRNKMKETI
Q8WXH0	2768	PDDILSQIRKCKVTHDGILAR
Q8WXH0	2993	NLKDRLTAIKCCILQVLKLKK
Q8WXH0	2994	LKDRLTAIKCCILQVLKLKKV
Q8WXH0	3098	SQRIEKAKCLCDEIIKKLNEN
Q8WXH0	3154	LSPKELDEKNCQDKLETSLHV
Q8WXH0	3204	AFQEQVWAEMCSIKAVTAIEK
Q8WXH0	3363	EAERYLENYKCYRKMEEDIYT
Q8WXH0	3438	RLRCTENDGICLLKIVSALWE
Q8WXH0	3564	FQEITSMKERCNKLLQKVQKN
Q8WXH0	4279	QQVLEQQLVGCQAMLTEIEHK
Q8WXH0	4324	ALSLKLKTVKCNLEKVQMMLQ
Q8WXH0	4414	FNAKKMWPQYCQHDNDTTQES
Q8WXH0	4623	CLEHTQAAAVCRSKSLKAGLD
Q8WXH0	4911	EGKQLVASVSCPELEGQIAKL
Q8WXH0	5348	QEVIGKLKGLCPSVAEIIEEK
Q8WXH0	5359	PSVAEIIEEKCQNTHKRWTQV
Q8WXH0	5601	GIGLNEKFLYCCEKWIQLLEK
Q8WXH0	5602	IGLNEKFLYCCEKWIQLLEKI
Q8WXH0	5758	EIHFQRRRTTCALTLEAGEKL
Q8WXH0	5815	FQSTVETWDQCEKKIKELKSR
Q8WXH0	6054	ELSKPVVYDVCDDQEIQKRLA
Q8WXH0	6103	DSDACANETECDSIQQTTRSL
Q8WXH0	6161	WLKSAERTAACPNSSEVLYTS
Q8WXH0	6350	VSRFHRRLTSCTPGLEDEKEA
Q8WYR1	91	LALLFYSTVLCTPHFPPDSDL
Q8WYR1	340	VEEDLETDGHCAERDSLLSTS
Q8WYR1	572	KRSHGTSPGACPPPRSQTPSP
Q8WYR1	751	RSRWSNLEKVCTSVNLNKACR
Q8WYR1	813	DKVQIIGSNSCPFAVCLDQDE
Q8WYR1	833	ERKILQSVVRCEVSPCYKPEK
Q8WYR1	869	QAAPDLCSLLCLPIMTFSGAL
Q8WZ42	1139	KVSYNKQTGECKLVISMTFAD
Q8WZ42	1375	FASNIKGNAICSGKLYVEPAA
Q8WZ42	1468	VFVLKPVSFKCLEGQTARFDL
Q8WZ42	1777	AYSRDSGIITCRATNKYGTDH
Q8WZ42	1902	DGIHYLDIVDCKSYDTGEVKV

Q8WZ42	2196	KEKDTMATFECETSEPFVKVK
Q8WZ42	2347	SFVIDGKKTTCKLKMKPRPIA
Q8WZ42	2368	ILQGLSDQKVCEGDIVQLEVK
Q8WZ42	2526	KLIVGRVETNCNLSVEKIKII
Q8WZ42	2544	KIIRGLRDLTCTETQNVVFEV
Q8WZ42	2873	EYTAVVGQLECKAKLFVETLH
Q8WZ42	2903	VPETKTASFECEVSHFNVPSM
Q8WZ42	3014	NGVEIKSTDKCQMRTKKLTHS
Q8WZ42	3079	VLEKKRAMFECEVSEPDITVQ
Q8WZ42	3311	AFPEDAAVYTCEAKNDYGVAT
Q8WZ42	3366	TSEGQPARFQCRVSGTDLKVS
Q8WZ42	3619	AKSLEKLGGPCPPHFLKELKP
Q8WZ42	3632	HFLKELKPIRCAQGLPAIFEY
Q8WZ42	3662	VTWFKENKQLCTSVYYTIIHN
Q8WZ42	3723	LEDTDMTDTPCKAKSTPEAPE
Q8WZ42	3980	YEPLVPSEHSCTEGGKILIES
Q8WZ42	4161	NLKMELRDALCAIIYEEIDIL
Q8WZ42	4360	NTEDHQGEYVCEALNDSGKTA
Q8WZ42	4455	TQVVDCGEYTCKASNEYGSVS
Q8WZ42	4466	KASNEYGSVSCTATLTVTEAY
Q8WZ42	4499	TFVGKAAKFICTVTGTPVIET
Q8WZ42	4550	LTIQDRGVYSCKASNKFGADI
Q8WZ42	4620	KLPPGKDYKICFEDKIATLEI
Q8WZ42	4643	AKLKDSGTYVCTASNEAGSSS
Q8WZ42	4779	IVRGTNALLQCEVSGTGPFEI
Q8WZ42	4814	YRLFSQKSLVCLEIFSFNSAD
Q8WZ42	4830	FNSADVGEYECVVANEVGKCG
Q8WZ42	4841	VVANEVGKCGCMATHLLKEPP
Q8WZ42	5179	GNKELVSGGSCYITKEALESS
Q8WZ42	5248	LKKGDATQLACKVTGTPPIKI
Q8WZ42	5299	VGIEDSGEYMCEAQNEAGSDH
Q8WZ42	5392	FVAADAGEYQCRVTNEVGSSI
Q8WZ42	5485	IEVKHDGKYVCQAKNDAGIQR
Q8WZ42	5674	LEGTDSGTYTCSATNKAGHNQ
Q8WZ42	5810	LRNGQSTTFECQITGTPKIRV
Q8WZ42	5861	ARVENSGTYVCEARNDAGTAS
Q8WZ42	5903	VVKYSDVELECEVTGTPPFEV
Q8WZ42	6047	VDNGHSGRYTCQAKNESGVER
Q8WZ42	6089	VTEKDPMTLECVVAGTPELKV
Q8WZ42	6236	LELEDTANYTCKVSNVAGDDA
Q8WZ42	6247	KVSNVAGDDACSGILTVKEPP
Q8WZ42	6303	DDVELVSGPKCFIGLEGSTSF
Q8WZ42	6465	TLKNAEVSLECELSGTPPFEV
Q8WZ42	6516	VDTSDIGEYHCKAQNEVGSDT
Q8WZ42	6529	QNEVGSDTCVCTVKLKEPPRF
Q8WZ42	6610	AEPANAGKYICQIKNDGGMRE
Q8WZ42	6782	KYQTTFSDNVCTLQLNSLDSS
Q8WZ42	6866	GARELVKGDRCNIYFEDTVAE
Q8WZ42	6959	DGFNITTSEKCNIVTTEKTCI
Q8WZ42	6968	KCNIVTTEKTCILEILNSTKR
Q8WZ42	6985	STKRDAGQYSCEIENEAGRDV
Q8WZ42	7270	VGKGDSGQYTCQATNDVGKDM
Q8WZ42	7406	ALKGSDVILQCEISGTPPFEV
Q8WZ42	7457	LEASDVGEYHCKATNEVGSDT
Q8WZ42	7470	TNEVGSDTCSCSVKFKEPPRF

Q8WZ42	7551	PEASNSGKYICQIKNDAGMRE
Q8WZ42	7593	VTTGNPFALECVVTGTPELSA
Q8WZ42	7714	DGNEIVSGPKCQSSFSENVCT
Q8WZ42	7723	KCQSSFSENVCTLNLSLLEPS
Q8WZ42	7900	DEYLISQSERCSITMTEKSTI
Q8WZ42	8160	LALGESGTFKCHVTGTAPIKI
Q8WZ42	8211	VGKGDAGQYTCYASNIAGKDS
Q8WZ42	8254	VKQDEFTRYECKIGGSPEIKV
Q8WZ42	8347	TLKGADVHLECELQGTPPFHV
Q8WZ42	8398	VDAADIGEYQCKATNDVGSDT
Q8WZ42	8409	KATNDVGSDTCVGSIALKAPP
Q8WZ42	8492	VEPANAGKYTCQIKNDAGMQE
Q8WZ42	8530	ESIKVTTGDTCTLECTVAGTP
Q8WZ42	8841	NGINVTPSQRCNITTTEKSAI
Q8WZ42	9056	TDRSLAGQYSCTATNPIGSAS
Q8WZ42	9101	AVVGESADFECHVTGTQPIKV
Q8WZ42	9223	NNIEIQPTSNCEITFKNNTLV
Q8WZ42	9345	VAKADSGDYVCKASNVAGSDT
Q8WZ42	9646	GDRHTLRVKNCQLKDQGNYRL
Q8WZ42	9658	LKDQGNYRLVCGPHIASAKLT
Q8WZ42	12117	VQLSDAGEYTCVLRLGNKEKT
Q8WZ42	12161	VVKGQPLYLSCELNKERDVVW
Q8WZ42	12254	VKPKGTAIFACDIAKDTPNIK
Q8WZ42	12369	KGELLRPSPTCEIKAEGGKRF
Q8WZ42	12434	VPERRQARFECVLTREANVIW
Q8WZ42	12522	VKEGETATFVCELSHEKMHVV
Q8WZ42	12611	AVEKDEITLKCEVSKDVPVKW
Q8WZ42	12660	ADLKDKGEYVCDCGTDKTKAN
Q8WZ42	12662	LKDKGEYVCDCGTDKTKANVT
Q8WZ42	12723	KGQPLTASPDCEIIEDGKKHI
Q8WZ42	12788	VFEKDEAKFECEVSREPKTFR
Q8WZ42	12966	GVEKDEVILQCEISKADAPVK
Q8WZ42	13018	KSDIGQYTCDCGTDKTSGKLD
Q8WZ42	13079	KGEALLQTPDCEIKEEGKIHS
Q8WZ42	13144	VTAGETATFDCELSYEDIPVE
Q8WZ42	13233	VEEGATAVLECEVSRENAKVK
Q8WZ42	13283	CTPEDIKTYTCDAKDFKTSCN
Q8WZ42	13322	VREKEMARFECELSRENAKVK
Q8WZ42	13411	VSETDTIKLVCEVSKPGAEVI
Q8WZ42	13500	ILEGEKAEFVCSISKESFPVQ
Q8WZ42	13588	VKEQQEVVFNCEVNTEGAKAK
Q8WZ42	13682	TMEKKSVTFWCKVNRLNVTLK
Q8WZ42	13771	VTEFDDAVFSCQLSREKANVK
Q8WZ42	13898	SEGKIHRLQICDIKPRDQGEY
Q8WZ42	14073	WVLATDRAESCEFTVTGLQKG
Q8WZ42	14356	TGYPRPTATWCFGDKVLETGD
Q8WZ42	14489	VQGKEYLFKVCARNKCGPGEP
Q8WZ42	14494	YLFKVCARNKCGPGEPAYVDE
Q8WZ42	14564	CPRGSDKWVACGEPVAETKME
Q8WZ42	14727	FDITDVTNESCLLTWNPPRDD
Q8WZ42	14859	LDPDTDKWVRCNKMPVKDTTY
Q8WZ42	15069	TVDTTVKDTKCTVTPLTEGSL
Q8WZ42	15168	VKAGKTAGPDCNFRVTDVIEG
Q8WZ42	15312	SENVVAKDPDCKPTIDLETHD
Q8WZ42	15410	NVKVIGLPGPCKDIKASDITK

Q8WZ42	15747	FEDIRKTSVLCKWEPPLDDGG
Q8WZ42	15787	WIVVTSTLRHCKYSVTKLIEG
Q8WZ42	15816	AENRFGPGPPCVSKPLVAKDP
Q8WZ42	16109	GRYVITATNSCGSKFAAARVE
Q8WZ42	16181	RQPIETERSKCDITGLLEGQE
Q8WZ42	16204	FRVIAKNKFGCGPPVEIGPIL
Q8WZ42	16276	PDFERVNKRLCPTTSFLVENL
Q8WZ42	16441	LAVTDIKAESCYLTWDAPLDN
Q8WZ42	16512	AVNKYGISDECKSDKVVIQDP
Q8WZ42	16637	DPIFPPGPPSCPEVKDKTKSS
Q8WZ42	16693	EPDKLITTCECVVPNLKELRK
Q8WZ42	16808	ENSSVIIIPECKRSHTGKYSI
Q8WZ42	16855	LKVSDITRGSCRLSWKMPDDD
Q8WZ42	16892	GKAWTKVNPDCGSTTFVVPDL
Q8WZ42	16993	TGTKKEAWRQCNKRDVEELQF
Q8WZ42	17043	VGPVTVKDQTCPPSIDLKEFM
Q8WZ42	17106	HVNKLVVDDTCTLVIPQSRRS
Q8WZ42	17201	WVHVSSEPKECTYTIPKLLEG
Q8WZ42	17732	NIVGQGKPSFCTKPITCKDEL
Q8WZ42	17817	KAKRSDSGKYCVVVENSTGSR
Q8WZ42	17898	PVTSASAKTTCKVSKLLEGKD
Q8WZ42	18096	WRRANHTPESCPETKYKVTGL
Q8WZ42	18253	LEVSEIRKDSCYLTWKEPLDD
Q8WZ42	18394	IKFKTVTNLECVVTGLQQGKT
Q8WZ42	18500	NFSSVLTIKNCLRRDTGEYQI
Q8WZ42	18599	KLKIPHLQKGCEYVFRVRAEN
Q8WZ42	18769	GSPILGYVVECQKPGTAQWNR
Q8WZ42	18945	LKVTNVTKENCTISWENPLDN
Q8WZ42	19478	DASPDEGWKRCNAAAQLVRKE
Q8WZ42	19549	MRKLVIVRAGCPIRLFAIVRG
Q8WZ42	19857	AHVYGKPHPTCKWKKGEDEVV
Q8WZ42	19959	SNITNYIVEKCDVSRGDWVTA
Q8WZ42	19978	TALASVTKTSCRVGKLIPGQE
Q8WZ42	20034	NARVTKVNKDCIFVAWDRPDS
Q8WZ42	20241	LLTVKAGTNVCLDATVFGKPM
Q8WZ42	20418	AKNPYDPPGRCDPPVISNITK
Q8WZ42	20565	KRADSDNWVRCNLPQNLQKTR
Q8WZ42	20680	KSTDFDTFLRCENVNKYDAGK
Q8WZ42	20699	GKYILTLENSCGKKEYTIVVK
Q8WZ42	20827	LKVRSVSKSSCSIGWKKPHSD
Q8WZ42	20918	DLDLKGLPDLCYLAKENSNFR
Q8WZ42	20972	AVNTTLIVYDCQKSDAGKYTI
Q8WZ42	21053	RDSVNNKWVTCASAVQKTTFR
Q8WZ42	21258	MKANHVNVPECAFTVTDLVEG
Q8WZ42	21456	WTVVSEDIQSCRHVATKLIQG
Q8WZ42	21558	AIKTPVSDLRCKVTGLQEGST
Q8WZ42	21764	ESFTLLIIPECNRYDTGKFVM
Q8WZ42	21851	YSTATTKCHKCTYKVTGLSEG
Q8WZ42	21949	THITTVKGLECVVRNLTEGEE
Q8WZ42	22176	YGVGPGITSACIVANYPFKVP
Q8WZ42	22631	RDKAGQRWIKCNKKTLTDLRY
Q8WZ42	22802	DLRKVVTIRACCTLRLFVPIK
Q8WZ42	22803	LRKVVTIRACCTLRLFVPIKG
Q8WZ42	22932	RKAYSTVATNCHKTSWKVDQL
Q8WZ42	23025	QTKGSDKWATCATVKVTEATI

Q8WZ42	23230	ATVARTTIKACRLKTGCEYQF
Q8WZ42	23236	TIKACRLKTGCEYQFRIAAEN
Q8WZ42	23623	VVDANVQTLSCKVTKLLEGNE
Q8WZ42	23713	RDKEGIRWTRCHKRLIGELRL
Q8WZ42	23759	PSPPSAYQKACDPIYKPGPPN
Q8WZ42	23794	SWSKPIYDGGCEIQGYIVEKC
Q8WZ42	23814	CDVSVGEWTMCTPPTGINKTN
Q8WZ42	23841	LEKHEYNFRICAINKAGVGEH
Q8WZ42	24014	RKSYAAVVTNCHKNSWKIDQL
Q8WZ42	24280	SWNPPLYTGGCQITNYIVQKR
Q8WZ42	24448	NSECYVARDPCDPPGTPEPIM
Q8WZ42	24599	GDKEIEESARCEIKNTDFKAL
Q8WZ42	24795	RDRSGIRWIKCNKRRITDLRL
Q8WZ42	24923	TEHQEYKIRVCALNKVGLGEA
Q8WZ42	25195	KWSIVAESKVCNAVVTGLSSG
Q8WZ42	25362	SWEPPAYTGGCQISNYIVEKR
Q8WZ42	25878	RDKEGVRWTKCNKKTLTDLRL
Q8WZ42	25979	KEAAADEWTTCTPPTGLQGKQ
Q8WZ42	26006	KENTEYNFRICAINSEGVGEP
Q8WZ42	26179	RKAYATITNNCTKTTFRIENL
Q8WZ42	26272	QTKGSEKWSTCTQVKTLEATI
Q8WZ42	26428	PGPIRIDEVSCDSITISWNPP
Q8WZ42	26444	SWNPPEYDGGCQISNYIVEKK
Q8WZ42	26661	RELPDGRWLKCNYTNIQETYF
Q8WZ42	26779	DNHTLLTVKDCIRRDTGQYVL
Q8WZ42	26869	ICEGELQMTSCKVTKLLKGNE
Q8WZ42	27351	LEKGQKNWVKCAVAKSTHHVV
Q8WZ42	27847	DKELQTNALVCVENTTDLASI
Q8WZ42	27950	WSMVSEHLEECIITTTKIIKG
Q8WZ42	28174	NYYFRVSAVNCAGQGEPIEMN
Q8WZ42	29033	RLAWALIEDKCEAQSYTAIKL
Q8WZ42	29235	GDWHKVNAEACVKTRYTVTDL
Q8WZ42	29267	AINGAGKGDSCEVTGTIKAVD
Q8WZ42	29586	LTGITNQLITCKAGSPFTIDV
Q8WZ42	29825	VKENKVPCLECNYKVTGLVEG
Q8WZ42	29918	SEVGDGRWLKCNYTIVSDNFF
Q8WZ42	30018	WTKGDKELDLCEKVSLQYTGK
Q8WZ42	30037	GKRATAVIKFCDRSDSGKYTL
Q8WZ42	30121	RLNWVIVEGECPTLSYVVTRL
Q8WZ42	30468	LVKVYDTPGPCPSVKVKEVSR
Q8WZ42	31009	QKVDQHEWTKCNTTPTKIREY
Q8WZ42	31095	IPIKGKPFPICKWTKEGQDIS
Q8WZ42	31140	GTYDLVLENKCGKKAVYIKVR
Q8WZ42	31383	SVTLQWEKPECDGGKEILGYW
Q8WZ42	31690	SWITNYVVEKCEAKEGAEWQL
Q8WZ42	31768	PTITAVTKDSCVVAWKPPASD
Q8WZ42	31876	VRYQSNATLVCKVTGHPKPIV
Q8WZ42	32029	ERKDAGFYVVCAKNRFGIDQK
Q8WZ42	32195	GRGEFGIVHRCVETSSKKTYM
Q8WZ42	32283	REIVSYVHQVCEALQFLHSHN
Q8WZ42	32472	NMVVSAARISCGGAIRSQKGV
Q8WZ42	32516	GEEGGHVKYVCKIENYDQSTQ
Q8WZ42	32596	KGVREVYDYYCRRTMKKIKRR
Q8WZ42	32703	VARNKYGEDSCKAKLTVTLHP
Q8WZ42	33375	TDDSGTYRAVCTNYKGEASDY

Q8WZ42	33664	EASKEIAKLTCVVESSVLRAK
Q8WZ42	33851	TDTSDSGLYTCTVKNSAGSVS
Q8WZ42	33864	KNSAGSVSSSCKLTIKAIKDT
Q8WZ42	34036	ASHRDEGILTCISKTKEGIVK
Q8WZ42	34047	ISKTKEGIVKCQYDLTLSKEL
Q8WZ42	34142	TIKAKNFRGQCSATASLMVLP
Q8WZ42	34291	GEPTPEVTWSCGGRKIHSQEQ
Q8WZ75	158	AVVGEQFTLECGPPWGHPEPT
Q8WZ75	436	AGAGEPSRPVCLLLEQAMERA
Q8WZ75	474	TLKRPEVIATCGVALWLLLLG
Q8WZ75	488	LWLLLLGTAVCIHRRRRARVH
Q8WZ75	858	GVGPKGGVLLCPPRPCLTPTP
Q92526	25	RARAALAVNICAARGLQDVLR
Q92526	233	KRVEDAFILICNVSLEYEKTE
Q92526	282	QKIIDLKDKVCAQSNKGFVVI
Q92526	343	VNSFEDLTVDCLGHAGLVYEY
Q92526	370	FTFIEECVNPCSVTLLVKGPN
Q92526	406	RAIKNAIEDGCMVPGAGAIEV
Q92526	499	AADAGVWDNYCVKKQLLHSCT
Q92526	508	YCVKKQLLHSCTVIATNILLV
Q92736	24	LRTDDEVVLQCTATIHKEQQK
Q92736	36	ATIHKEQQKLCLAAEGFGNRL
Q92736	47	LAAEGFGNRLCFLESTSNSKN
Q92736	361	TSEIKYGDSVCYIQHVDTGLW
Q92736	548	AALIRGNRKNCAQFSGSLDWL
Q92736	633	VAVRSNQHLICDNLLPGRDLL
Q92736	917	VRDDNKRQHPCLVEFSKLPEQ
Q92736	948	ETLKTLLALGCHVGISDEHAE
Q92736	1078	DQDHAARAEVCSGTGERFRIF
Q92736	1122	DMRVGWSRPGCQPDQELGSDE
Q92736	1230	DVSTLKYFTICGLQEGYEPFA
Q92736	1582	KSEHKNPVPQCPPRLHVQFLS
Q92736	1621	ISERQGWLVQCLDPLQFMSLH
Q92736	1665	YHTLRLYSAVCALGNHRVAHA
Q92736	1988	NFKDDKSECPCPEEIRDQLLD
Q92736	2308	YLDFLRFAVFCNGESVEENAN
Q92736	2330	VVRLLIRRPECFGPALRGEGG
Q92736	2461	VVEPDMSAGFCPDHKAAMVLF
Q92736	2559	LHTVYRLSKGCSLTKAQRDSI
Q92736	2991	YFLSAASRPLCSGGHASNKEK
Q92736	3009	KEKEMVTSLFCKLGVLVRHRI
Q92736	3032	FGNDATSIVNCLHILGQTLDA
Q92736	3481	KRLLPIGLNICAPGDQELIAL
Q92736	3574	KSKRVGRRHYCLVEHPQRSKK
Q92736	3680	LFSRTALTEKCKLEEDFLYMA
Q92736	3928	NTLTEYIQGPCTGNQQSLAHS
Q92736	4193	KEKMELFVNFCEDTIFEMQLA
Q92736	4586	AILHTVISFFCIIGYYCLKVP
Q92736	4891	KEDMETKCFICGIGNDYFDTV
Q92736	4957	RCWEFFPAGDCFRKQYEDQLN
Q92781	36	ASNAFVFITGCDSGFGRLLAL
Q92781	176	GRLAANGGGYCVSKFGLEAFS
Q92781	257	KMQQRIMNLICDPDLTKVSRC
Q92793	367	QLVLLLHAHKCQRREQANGEV
Q92793	380	REQANGEVRACSLPHCRTMKN

Q92793	409	QAGKACQVAHCASSRQIISHW
Q92793	427	SHWKNCTRHDCPVCLPLKNAS
Q92793	430	KNCTRHDCPVCLPLKNASDKR
Q92793	845	MLGPQASQLPCPPVTQSPLHP
Q92793	1178	NRKTSRVYKFCSKLAEVFEQE
Q92793	1199	IDPVMQSLGYCCGRKYEFSPQ
Q92793	1212	RKYEFSPQTLCCYGKQLCTIP
Q92793	1213	KYEFSPQTLCCYGKQLCTIPR
Q92793	1219	QTLCCYGKQLCTIPRDAAYYS
Q92793	1237	YYSYQNRYHFCEKCFTEIQGE
Q92793	1308	YDIIWPSGFVCDNCLKKTGRP
Q92793	1311	IWPSGFVCDNCLKKTGRPRKE
Q92793	1421	GMHVQEYGSDCPPPNTRRVYI
Q92793	1474	LGYVTGHIWACPPSEGDDYIF
Q92793	1486	PSEGDDYIFHCHPPDQKIPKP
Q92793	1690	SLRRSKWSTLCMLVELHTQGQ
Q92793	1732	VCEDYDLCINCYNTKSHAHKM
Q92793	1790	VHACQCRNANCSLPSCQKMKR
Q92793	1816	KGCKRKTNGGCPVCKQLIALC
Q92793	1819	KRKTNGGCPVCKQLIALCCYH
Q92793	1827	PVCKQLIALCCYHAKHCQENK
Q92793	1838	YHAKHCQENKCPVPFCLNIKH
Q92820	134	DGDYFPVWGTCLGFEELSLLI
Q92820	148	EELSLLISGECLLTATDTVDV
Q92922	462	PSYASWFDYNCIHVIERRALP
Q92922	511	NPQEYLTSTACRRNLTGDVCA
Q92922	520	ACRRNLTGDVCAVMRVHAFLE
Q92945	176	QINKIQQDSGCKVQISPDSGG
Q92945	436	EMTFSIPTHKCGLVIGRGGEN
Q93008	147	VSGWKFEIHRCIINNTHRLVE
Q93008	262	IAALIKPFGQCYEFLTLHTVK
Q93008	673	FLLKDGQLWLCAPQAKQIWKC
Q93008	749	CFERFFKAVNCREGKLVAKRR
Q93008	819	VVIHEDFIQSCFDRLKASYDT
Q93008	831	DRLKASYDTLCVLDGDKDSVN
Q93008	918	TNDTIGSVRRCILNRIKANVA
Q93008	1237	DEASRYMPDICVIRAIQKIIW
Q93008	1251	AIQKIIWASGCGSLQLVFSPN
Q93008	1295	CCEALEVMTLCFALIPTALDA
Q93008	1323	QTFIIDLLLHCHSKTVRQVAQ
Q93008	1451	QTSEKKFHIGCEKGGANLIKE
Q93008	1492	ELPAEQAIPVCGSPPTINAGF
Q93008	1512	FELLVALAVGCVRNLKQIVDS
Q93008	1727	SFADQKICQGCPHRYECEESF
Q93008	1771	DLLEGANAYHCEKCNKKVDTV
Q93008	1808	KRFDYDWERECAIKFNDYFEF
Q93008	2019	YFQFMKKLLTCNGVYLNPPPG
Q93008	2107	SNRFSEYLLECPSAEVRGAFA
Q96FJ0	38	ERVRALSKLGCNITISEDITP
Q96FJ0	98	KLPNHRDYQQCAVPEKQDIMK
Q96FJ0	213	SEQIDGSALSCFSTHQNNSLL
Q96FJ0	268	VQNLVVEGLRCVVLPEDLCHK
Q96FJ0	379	LMLPEAIAIVCSPKHKDTGIF
Q96FJ0	420	HTKEPRLFSICKHVLVKDIKI
Q96HH9	131	TEEPLKQSFTCALQKEILYQG

Q96HH9	151	GKLFVSENWICFHSKVFGKDT
Q96HH9	355	HILIFYAIVVCALIISTFYMR
Q96ME7	408	TDIKKYHRIQCPNQGCEAVYS
Q96ME7	430	VSGLKAHLGSCTLGNFVAGKY
Q96ME7	540	NNEELVVSASCKEPEQEPVPA
Q9UPN3	37	RSERSGSLSPCPPGDTLPWNL
Q9UPN3	443	ANKIQNGALNCEEKLTLAKNT
Q9UPN3	480	ESDVIMYIQECEGLIRQLQVD
Q9UPN3	666	ETLGKLETQYCKLKETSSFRM
Q9UPN3	815	LQDSIKRKYSCDHNTSLSRLE
Q9UPN3	877	LKNTISVKAVCDYRQIEITIC
Q9UPN3	887	CDYRQIEITICKNDECVLEDN
Q9UPN3	1025	RLRLEEEVEACKARFQHLMKS
Q9UPN3	1283	IQEVLGDYRACHGTLIKWIEE
Q9UPN3	1522	NALNKAYHDLCDGSANQLQQL
Q9UPN3	1806	RHNLIDQDMACAILIRQLQTG
Q9UPN3	1953	NINPGAAVLPCSKSHPKATAS
Q9UPN3	2062	QNKEYPDREDCTTEKGKKTTV
Q9UPN3	2278	EDSGREIFLSCSHPLELLEEA
Q9UPN3	2346	MFQGFFDSQTCESLTTEEVIN
Q9UPN3	2387	GVLDPRTQTLCSVKDAVTVGL
Q9UPN3	2787	QIEKQEGIEVCALQNEFLGKD
Q9UPN3	2854	SREISLKEFGCKDQRKPRMSS
Q9UPN3	2895	QVSLTHPYSECDFKLKEVARN
Q9UPN3	2956	EVILEVQETYCETSGKLPSEQ
Q9UPN3	3085	NEGKVNNLSLCLTLKPEENLS
Q9UPN3	3173	TKHQISSSNECKEKSYQEVSF
Q9UPN3	3315	SEEKTVSLTVCSAVKTEKTPQ
Q9UPN3	3407	LNLAELQDLLCQAKVLERELK
Q9UPN3	4325	VKEREKDASSCQEQLDEFRKL
Q9UPN3	4388	SKGTLVEEINCKGTSLENLIM
Q9UPN3	4825	DDKQSQQAKNCPISAKLERLQ
Q9UPN3	4906	TADRQSRLKDCMQKAQKYQWH
Q9UPN3	4927	VEDLVPWIEDCKAKMSELRVT
Q9UPN3	5049	EIFDALGSQACSNKNLEKLRA
Q9UPN3	5131	NKLEGIGQFHCRVREMFSQLA
Q9UPN3	5214	KRELEALNKQCGKLTERGKAR
Q9UPN3	5930	IPAEVDKIRECISDNKSATVE
Q9UPN3	6075	RILGADLIFACGETEKPEVRK
Q9UPN3	6646	GEVRDKWDTVCGKSVERQHKL
Q9UPN3	6840	VAMGEVILAVCHPDCITTIKH
Q9UPN3	6844	EVILAVCHPDCITTIKHWITI
Q9UPN3	7133	EDEVTRQVAQCKCAKRFQVEQ
Q96Q15	216	LLQELRQEGACCLGLLCASLS
Q96Q15	1085	MVVEALCELHCPEAIQGIAVW
Q96Q15	1137	EHLCAMTGVDCCISSFDKSVL
Q96Q15	1245	SSFESGKFVECTEQLELLPGE
Q96Q15	1299	IEVQLLRSSVCLATALNPIEQ
Q96Q15	1748	DRIFSLYKLSCSAYFTFLKLN
Q96Q15	1846	YVRQSICNLLCRVAQDSPHLI
Q96Q15	1929	LNEDQAMMQDCYSKIVDKLSS
Q96Q15	2358	GEVVHIDYNVCFEKGKSLRVP
Q96Q15	2673	QWKTWMEELICNTTVERCQEL
Q96Q15	2773	SLASVIISALCTLTRRNLMME
Q96Q15	3072	VNVKTLFRNSCFSEDQMAKPI

Q96Q15	3138	AESKVSVDDLCKKAVEHNIQI
Q96Q15	3303	SQRASQVTFLCSNIIHFESLR
Q96Q15	3401	TEKEQQIETVCETIQNLVDNI
Q96Q15	3587	TVPGTGKSVACSPKKAVRDPK
Q96QE4	636	LPFLQYINLGCNLITKLSLGT
Q96QE4	729	CQFKNSIEAVCKTVKLHCNTA
Q96QE4	740	KTVKLHCNTACLTNSIHCPEE
Q96QE4	924	VILIILIIIFCLIEVNSHKRA
Q96RW7	175	SQVVFVLTGDCDDRTHIGYKV
Q96RW7	541	VTPGERAVLTCLIISAVDYNL
Q96RW7	681	AAPKDAGIYGCLASNSAGTDK
Q96RW7	867	LWASDKGTYICEAENQFGKIQ
Q96RW7	960	VQLQDGGEYTCVASNVAGTNN
Q96RW7	1051	PGGEESGEYVCTATNTAGYAK
Q96RW7	1101	VLAGEEVTLPCEVKSLPPPII
Q96RW7	1192	VQVGQRVDIPCNAQGTPLPVI
Q96RW7	1241	ATPSDAGIYTCVATNIAGTDE
Q96RW7	1288	RVANQRIEFPCPAKGTPKPTI
Q96RW7	1338	VTPYDNGEYICVAVNEAGTTE
Q96RW7	1475	VVLNRDVALECQVKGTPFPDI
Q96RW7	1525	ARRNDKGRYQCTVSNAAGKQA
Q96RW7	1712	AQEIDRGQYICVATSVAGEKE
Q96RW7	1805	AQVSNTGLYRCMAANTAGDHK
Q96RW7	1848	VVKYKPVALQCIANGIPNPSI
Q96RW7	1898	TLLEDAGRYTCVATNAAGETQ
Q96RW7	2033	VVVNNPVRLECEARGIPAPSL
Q96RW7	2174	AQVQDTGRYTCEATNVAGKTE
Q96RW7	2218	VIEGNLISLLCESSGIPPPNL
Q96RW7	2408	VVEKNSVSLTCEASGIPLPSI
Q96RW7	2457	TTMEDAGQYTCVVRNAAGEER
Q96RW7	2501	VKEKQSVTLTCEVTGNPVPEI
Q96RW7	2597	VILNSPTSLVCEAYSYPPATI
Q96RW7	2646	AQEDNAGRYSCVATNEAGEMI
Q96RW7	2848	AKVEDAGRYTCVAVNEAGEDS
Q96RW7	2943	TQITDIGRYVCVAENTAGSAK
Q96RW7	3173	ETISNPVTLTCDATGIPPPTI
Q96RW7	3268	VLLGENVELVCNANGIPTPLI
Q96RW7	3364	TLVDTSINIECRATGTPPPQI
Q96RW7	3413	AQVSDVAVYTCVASNRAGVDN
Q96RW7	3550	VIVNNPLELTCIASGIPAPKM
Q96RW7	3692	ADLGDTANYTCVASNIAGKTT
Q96RW7	3783	AHVTDTGRYLCMATNAAGTDR
Q96RW7	3825	VIVNVQTTLACEATGIPKPSI
Q96RW7	3876	PSVDDTATYECTVTNGAGDDK
Q96RW7	4009	VILNNPILLPCEATGTPSPFI
Q96RW7	4281	LNKGEQLRLSCKATGIPLPKL
Q96RW7	4328	VSKEDSGTYVCTAENSVGFVK
Q96RW7	4371	EPLGGNAILNCEVKGDPTPTI
Q96RW7	4419	TVNEDAGDYTCVATNEAGVVE
Q96RW7	4461	INAGGKIILNCQATGEPQPTI
Q96RW7	4509	AQKEDTSEFECVARNLMGSVL
Q96RW7	4545	WSAWRACSVTCGKGIQKRSRL
Q96RW7	4556	GKGIQKRSRLCNQPLPANGGK
Q96RW7	4697	TQMQVCNERNCPIHGKWATWA
Q96RW7	4716	WASWSACSVSCGGGARQRTRG

Q96RW7	4830	WHSWSQCSASCGGGEKTRKRL
Q96RW7	4841	GGGEKTRKRLCDHPVPVKGGR
Q96RW7	4868	TQVTRCNVQACPGGPQRARGS
Q96RW7	5092	SISKGDRSNQCPSGFTLDSVG
Q96RW7	5217	FNAIGSFHCGCEPGYQLKGRK
Q96RW7	5245	RQNVCRPDQHCKNTRGGYKCI
Q96RW7	5258	TRGGYKCIDLCPNGMTKAENG
Q96RW7	5270	NGMTKAENGTCIDIDECKDGT
Q96RW7	5457	KNTFGSYQCICPPGYQLTHNG
Q96RW7	5503	RGSYQCIDTPCPPNYQRDPVS
Q96RW7	5516	NYQRDPVSGFCLKNCPPNDLE
Q96RW7	5520	DPVSGFCLKNCPPNDLECALS
Q99250	180	SLIKILARGFCLEDFTFLRDP
Q99250	650	MNGKMHSAVDCNGVVSLVGGP
Q99250	742	FANMCLIWDCCKPWLKVKHLV
Q99250	768	DPFVDLAITICIVLNTLFMAM
Q99250	910	MQLFGKSYKECVCKISNDCEL
Q99250	912	LFGKSYKECVCKISNDCELPR
Q99250	918	KECVCKISNDCELPRWHMHDF
Q99250	1181	FTEDCVRKFKCCQISIEEGKG
Q99250	1182	TEDCVRKFKCCQISIEEGKGK
Q99250	1386	DVSVVNNYSECKALIESNQTA
Q99250	1542	VFDISIMILICLNMVTMMVET
Q99250	1578	LVFIVLFTGECVLKLISLRYY
Q99250	1706	NFETFGNSMICLFQITTSAGW
Q99250	1731	APILNSGPPDCDPDKDHPGSS
Q99250	1940	SIYKKDKGKECDGTPIKEDTL
Q99666	50	KEYDLAKKYICTYINVQERDP
Q99666	105	DLVLKIAELLCKNDVTDGRAK
Q99666	521	YQPLCLPFPVCKQLCTERQKS
Q99666	537	ERQKSWWDAVCTLIHRKAVPG
Q99666	1108	LMRREQVLKVCANHWITTTMN
Q99666	1405	VMRRDQVLKLCANHRITPDMS
Q99666	1431	GTERVWVWTACDFADGERKVE
Q99798	27	VRQYHVASVLCQRAKVAMSHF
Q99798	126	SKVAVPSTIHCDHLIEAQVGG
Q99798	226	VMAGIPWELKCPKVIGVKLTG
Q99798	332	FKDHLVPDPGCHYDQLIEINL
Q99798	385	LDIRVGLIGSCTNSSYEDMGR
Q99798	410	AKQALAHGLKCKSQFTITPGS
Q99798	451	GIVLANACGPCIGQWDRKDIK
Q99798	740	KDFTPGKPLKCIIKHPNGTQE
Q99996	882	NYQELQEEYACLLKVKDDLED
Q99996	1156	YMENEKDKALCSLKEELIFAQ
Q99996	1232	LGEYYTPALKCEVNAEDKENS
Q99996	1379	TEQNYEAEIHCLQKRLQAVSE
Q99996	1417	DAQRTMYPGSCVKKNIDGTIE
Q99996	1556	DPHDIPESKDCVLTISEEMFS
Q99996	1966	IIDRLEQELLCASNRLQELEA
Q99996	2022	KLMKEKLEVQCQAEKVRDDLQ
Q99996	2530	VSAKDLELTQCYKQIKDMQEQ
Q99996	2889	ECGTLKAVIQCLRSKEVFGFY
Q99996	3085	QGVEYQAAMECLQKADRRSLL
Q99996	3525	ELEMIRQKLQCVASKLQVLPQ
Q9BS86	207	ILSKLLLDLSCEISLLKSECH

Q9BS86	248	SLDTEKGPKRCTDHNCEPYKR
Q9BS86	253	KGPKRCTDHNCEPYKRLFKAK
Q9BS86	319	NVQQHPKCPECCVICSPGSYN
Q9BS86	339	NPRDGIHCLQCNSSLVYGAKT
Q9BUF5	12	REIVHIQAGQCGNQIGTKFWE
Q9BUF5	129	VVRKECEHCDCLQGFQLTHSL
Q9BUF5	303	MFDARNMMAACDPRHGRYLTV
Q9BUF5	354	WIPNNVKVAVCDIPPRGLKMA
Q9BYJ4	15	ILLNVQEEVTCPICLELLTEP
Q9BYJ4	18	NVQEEVTCPICLELLTEPLSL
Q9BYJ4	97	SPDNGKKRDLCDHHGEKLLLF
Q9BYJ4	108	DHHGEKLLLFCKEDRKVICWL
Q9BYJ4	119	KEDRKVICWLCERSQEHRGHH
Q9BYJ4	140	TVLTEEVFKECQEKLQAVLKR
Q9BYJ4	298	QMFRELTAVRCYWVDVTLNSV
Q9BYJ4	463	GSLIYKFSKCCFSQPVYPYFN
Q9BYJ4	477	PVYPYFNPWNCPAPMTLCPPS
Q9BYZ2	84	IIGTGSVGMACAISILLKGLS
Q9BYZ2	180	ISSIVQYSPHCKLIIVSNPVD
Q9BYZ2	212	FPKNRIIGSGCNLDTARFRFL
Q9H3G5	271	EKQKKYFQKQCHECIEHIRKQ
Q9H3G5	274	KKYFQKQCHECIEHIRKQNWF
Q9H3G5	311	DPSYFQNVTGCSNYYNFLRCT
Q9H3G5	320	GCSNYYNFLRCTEPEDQLYYV
Q9H4A3	86	EHRFFRRSVICDSNATALELP
Q9H4A3	250	DTETTVEVAWCELQDRKLTKS
Q9H4A3	296	SWESTVKGKKCIVLVTELMTS
Q9H4A3	547	AQEMVESGYVCEGDHKTMAKA
Q9H4A3	1135	VSNKGDRVVECQLETHNRKMV
Q9H4B7	12	REIVHIQIGQCGNQIGAKFWE
Q9H4B7	303	MFDARNTMAACDLRRGRYLTV
Q9H4B7	354	WIPNNVKVAVCDIPPRGLSMA
Q9H7X3	130	WKGRPFPCGACGRSFKCSSDA
Q9H7X3	214	TGEKPYACADCGKAFGQRSDA
Q9H7X3	242	TGERLYACGECGKRFLHSSNV
Q9H7X3	326	TGEKPHQCGHCGRAFRALSGF
Q9H7X3	354	TGEKPFRCTECGRAFRLSFHL
Q9H8N7	54	QPFTTSDDTPCQEQPKEVLKA
Q9H8N7	117	WLLEQKLQVCCRVEEVWLAEL
Q9H8N7	200	EANFSASRAACDPWKESGDIS
Q9H8N7	282	RKNSVKVMYKCLWPNCGKVLR
Q9NQ38	44	QAFMKNGKLFCPQDKKFFQSL
Q9NQ38	97	PKATAPTELNCDDFKKGERDG
Q9NQ38	225	RIRRNAEKDFCKEYEKQVRNG
Q9NQ38	632	KVKREAEKETCDEFRRLLQNG
Q9NQ38	743	GKSYNNQCTMCKAKLEREAER
Q9NQ38	1006	YRVLPRIGYLCPKDLKPVCGD
Q9NQ38	1046	TNTHIRSTGKCEESSTPGTTA
Q9NS25	16	SVRRLKRSVPCESNEANEANE
Q9NYC9	171	DVRRHAHSLQCDLSVILEQVK
Q9NYC9	343	PQLRPLLHVVCLIWATCKSYR
Q9NYC9	366	GRLTVLLQEICNLLIQQASNY
Q9NYC9	787	ETLNWKTEGICDYVTEITSSI
Q9NYC9	901	LLNGFFLAIECSLKYLLENTE
Q9NYC9	912	SLKYLLENTECKAGLTPIFEA

Q9NYC9	946	SLESGVKGGFCDIVEGLITSI
Q9NYC9	1004	ERVQRMMGLCCGYQSTFSQYS
Q9NYC9	1295	NVPDYKQLRQCRKEVCQLKEL
Q9NYC9	1300	KQLRQCRKEVCQLKELWDTIG
Q9NYC9	1336	NINVEAMELECKQFARHIRNL
Q9NYC9	1584	EKLEDIQGRLCLCEKALAEYL
Q9NYC9	1780	KGDRQKIMTICTIDVHARDVV
Q9NYC9	1821	RHRWDDEVKHCFANICDAQFL
Q9NYC9	1826	DEVKHCFANICDAQFLYSYEY
Q9NYC9	1852	RLVITPLTDRCYITLTQSLHL
Q9NYC9	1905	NCSEQMDYKSCGNIYKGLAQT
Q9NYC9	2004	AMVVPDFELICEIMLVAEGFI
Q9NYC9	2325	TILFDKYLPTCLDTLRTRFKK
Q9NYC9	2365	LLTTEDIPADCPKEIYEHYFV
Q9NYC9	2696	FQGILFSSVECVKSTWDLIRL
Q9NYC9	2808	VLFEDAMRHVCHINRILESPR
Q9NYC9	2954	RIRRQLKVTLCFSPVGNKLRV
Q9NYC9	2975	RSRKFPAIVNCTAIHWFHEWP
Q9NYC9	3064	LLHRHRKELKCKTERLENGLL
Q9NYC9	3237	NFNKENIHENCLKAIRPYLQD
Q9NYC9	3280	INIVRFYEVFCDVEPKRQALN
Q9NYC9	3377	QNFKQQERTLCGDILLITAFI
Q9NYC9	3543	GRFIKIGDKECEYNPKFRLIL
Q9NYC9	3749	YQYTIRGLFECDKLTYLAQLT
Q9NYC9	3832	KSWKKFVESECPEKEKLPQEW
Q9NYC9	3852	WKNKTALQRLCMLRAMRPDRM
Q9NYC9	4378	EWPLDQMALQCDMTKKNREEF
Q9NYC9	4448	DKQDCRSVYSCPVYKTSQRGP
Q9P2E9	1323	AEFEEAQTSACRLQEELEKLR
Q9P2Q2	183	ALKEHPSLAYCEDRVIEHYKK
Q9P2Q2	300	SGIAVHTWYACPALIKSIWAM
Q9P2Q2	413	RQRLEELKKLCLREAELTGKL
Q9UBX3	23	GGLASCGAACCTHPLDLLKVH
Q9UBX3	217	FIAGGCATFLCQPLDVLKTRL
Q9UI46	339	KAKRLSVTALCWNPKYRDLFA
Q9UI46	388	YMFSSNSGVMCLDIHVDHPYL
Q9UI46	498	PEGLQLHPVGCGTAFDFHKEI
Q9UI46	559	NPYHTKVFMSCSSDWTVKIWD
Q9UI46	621	DLAINKYEAICNQPVAAKKNR
Q9UJ83	166	RSSIYGRPGACYVDIPADFVN
Q9UJ83	261	KGVVPDNHPYCVGAARSRALQ
Q9UJ83	307	PDVKFIQVDICAEELGNNVKP
Q9UJ83	392	YHVQEQLPRDCFVVSEGANTM
Q9UJ83	511	QDATAVVPPMCLLPNSHYEQV
Q9UKU0	108	RGLSISGNGPCLGFRKPKQPY
Q9UKU0	336	FERVIQSVVYCHGGRVGFFQG
Q9UKU0	427	FNKIQASLGGCVRMIVTGAAP
Q9UKU0	463	QVYEGYGQTECTAGCTFTTPG
Q9UKU0	486	TSGHVGAPLPCNHIKLVDVEE
Q9UKU0	625	RGIEGTYADLCTNKDLKKAIL
Q9ULB1	15	LLQRGGCFLLCLSLLLLGCWA
Q9ULB1	50	WTRFPKWNACCESEMSFQLKT
Q9ULB1	97	GRLQLSFSIFCAEPATLLADT
Q9ULB1	473	MKIHGVVAFKCENVATLDPIT
Q9ULB1	784	GRVKLTVNLDCIRINCNSSKG

Q9ULB1	789	TVNLDCIRINCNSSKGPETLF
Q9ULB1	891	DLCKNGDIDYCELNARFGFRN
Q9ULB1	1072	LPDLISDALFCNGQIERGCEG
Q9ULB1	1080	LFCNGQIERGCEGPSTTCQED
Q9ULB1	1414	VVGIVAAAALCILILLYAMYK
Q9ULW0	198	EKAKGRHTVPCMPPAKQKFLK
Q9ULW0	301	PSSPARVTKGCTIVKPFNLSQ
Q9ULW0	383	QTKHRARAVTCKSTAELEAEE
Q9ULW0	462	DEHFEFHSRPCPTKILEDVVG
Q9ULW0	536	QIPEARTVEICPFSFDSRDKE
Q9ULW0	594	VKNVTQIEPFCLETDRRGALK
Q9UPA5	170	QIAPLPSSTLCPICKTSDLTS
Q9UPA5	468	MPKERAICPLCQAELNVGSKS
Q9UPA5	515	VEKTEWLCLNCQTKRLLEGSL
Q9UPA5	1582	VHASASTSPLCSPTETQPTTH
Q9UPA5	1770	DFGQGGGSPVCLAQVKQVEQA
Q9UPA5	1913	MVYKLPFGSSCTGTFHPAPSV
Q9UPA5	2610	RRRRARRSADCSVQTDDEDSA
Q9UPA5	3283	GVLDGPTLPCCYARGEEESEE
Q9UQ13	144	KLQSLPAEVGCLVNLMTLALS
Q9UQ13	306	LSAIPRSLAKCSALEELNLEN
Q9Y230	227	AMGSQTKFVQCPDGELQKRKE
Q9Y230	375	KDTKQILRIRCEEEDVEMSED
Q9Y230	413	IQLITAASLVCRKRKGTEVQV
Q9Y277	8	XXXMCNTPTYCDLGKAAKDVF
Q9Y277	36	MVKIDLKTKSCSGVEFSTSGH
Q9Y277	65	SGNLETKYKVCNYGLTFTQKW
Q9Y277	229	FGIAAKYMLDCRTSLSAKVNN
Q9Y2Q0	221	SLMRISGRIECESPNRHLYDF
Q9Y2Q0	571	RTPSGKLRLYCKGADTVIYDR
Q9Y2Q0	699	QETAINIGHSCKLLKKNMGMI
Q9Y2Q0	862	YNRVSKCILYCFYKNIVLYII
Q9Y2Q0	945	DFNTKVFWVHCLNGLFHSVIL
Q9Y2Q0	993	FVYTFVVITVCLKAGLETSYW
Q9Y5R2	139	TTIEWMKKPRCGVPDHPHLSR
Q9Y5R2	470	YPHSLGELGSCLPREGIDTAL
Q9Y5R2	569	PRNILRDWMGCNQKEVERRKE
Q9Y6L6	24	QPSENKKTRYCNGLKMFLAAL
Q9Y6L6	430	SFYLLYFFILCENKSVAGLTM
Q9Y6L6	474	NCDESQWEPVCGNNGITYISP
Q9Y6L6	504	GNKKPIVFYNCSCLEVTGLQN
Q9Y6L6	524	NRNYSAHLGECPRDDACTRKF
Q9Y6V0	535	PPSQGLPKTICPLCNTTELLL
Q9Y6V0	538	QGLPKTICPLCNTTELLLHVP
Q9Y6V0	1005	LEKSPKPESTCPLCKTELNIG
Q9Y6V0	1264	KTIKEQPQPPCTAKPDQEKED
Q9Y6V0	2871	VDLTAGRRAVCCDVVYKLPFG
Q9Y6V0	2872	DLTAGRRAVCCDVVYKLPFGR
Q9Y6V0	2884	VVYKLPFGRSCTAQQPATTLP
Q9Y6V0	4995	KKVIKKKTRVCRHDREPSFNE
Q9Y6V0	5039	FMKKTLIGEACIWLDKVDLRK
Q9Y6Z4	24	RLRAVACAPHCFGPRLRCLHH
Q9Y6Z4	69	PSARECCSIVCMAAKEVSAPK
Q9Y6Z4	153	DPSTCHLAKGCSPAWGFLPQA
Q33557	162	LNKYGRPLLGCTIKPKLGLSA

Q33557	211	FMRWRDRFLFCTEAIYKSQAE
Q8VXH1	303	RTAYITTLNVCQAYTLKRIRD
A2AUS0	33	LNQQRQQGQLCDVSIVVQGHI
A2AUS0	59	AVLAASSPYFCDQVLLKNSRR
A2AUS0	375	GFSATDKLYPCQCGKSFTHKS
A2AUS0	377	SATDKLYPCQCGKSFTHKSQR
A2AUS0	405	LGLRPYGCSVCGKKFKMKHHL
A2AUS0	430	KIHTGIKPYECNICAKRFMWR
A2AUS0	433	TGIKPYECNICAKRFMWRDSF
A2AUS0	450	RDSFHRHVTSCTKSYEAAKAE
P27612	12	ASGASRYRLSCSLPGHELDVR
P27612	116	YILKGHKDTVCSLSSGKFGTL
P27612	183	KTIKLWKAGRCERTFLGHEDC
P27612	223	SIRRWQITGECLEVYFGHTNY
P27612	263	RSLRIWKHGECAQTIRLPAQS
P27612	584	VLLEKILSLICNNSSEKPTAQ
B1AVT9	351	DMCIHVCRLGCDRPVKTFQGH
B1AVT9	380	WDPSGMLLASCSDDMTLKIWS
B1AVT9	447	VRLWDVERGVCIHTLTKHQEP
B1AVT9	503	YRGTGGIFEVCWNARGDKVGA
B1AXT2	42	IFHDALKGWSCCRKRTVDFSE
B1AXT2	43	FHDALKGWSCCRKRTVDFSEF
B1AXT2	59	DFSEFLNIKGCTVGLHCAEKL
B1AXT2	65	NIKGCTVGLHCAEKLPEVPPQ
B1AXT2	187	RFHEGMKSWSCCGIQTLDFGA
B1AXT2	204	DFGAFLAQPGCRVGRHDWAKQ
O08759	97	LELYKINAKLCDPHPSKKGAS
O08759	280	ALVYLSPNVECDLTYHNVYTR
O08759	321	EYLEMALPLFCKAMCKLPLEA
O08759	770	FRPEEIELLICGSRNLDFQAL
O08759	853	DTERLPTSHTCFNVLLLPEYS
O88307	362	ADAAEDQVFVCVSHSNNSTNL
O88307	625	VNATDALGVPCTENDYKLWSP
O88307	643	WSPSDERGNECLLGHKTVFKR
O88307	660	VFKRRTPHATCFNGEDFDRPV
O88307	675	DFDRPVVVSNCSCTREDYECD
O88307	684	NCSCTREDYECDFGFKMSEDL
O88307	699	KMSEDLSLEVCVPDPEFSGKP
O88307	716	SGKPYSPPVPCPVGSSYRRTR
O88307	736	RGYRKISGDTCSGGDVEARLE
O88307	752	EARLEGELVPCPLAEENEFIL
O88307	801	VALDFDYERNCLYWSDLALDT
O88307	942	WIYWTDAYLDCIERITFSGQQ
O88307	1021	YKGKNAGSNACVPQPCSLLCL
O88307	1117	SDERNCPTTVCDADTQFRCQE
O88307	1131	TQFRCQESGTCIPLSYKCDLE
O88307	1177	SGMCIRSSWVCDGDNDCRDWS
O88307	1199	EANCTAIYHTCEASNFQCHNG
O88307	1206	YHTCEASNFQCHNGHCIPQRW
O88307	1244	SCEKKCNGFHCPNGTCIPSSK
O88307	1256	NGTCIPSSKHCDGLRDCPDGS
O88307	1271	DCPDGSDEQHCEPFCTRFMDF
O88307	1283	PFCTRFMDFVCKNRQQCLFHS
O88307	1359	DCGDYSDEANCENPTEAPNCS
O88307	1381	YFQFHCENGHCIPNRWKCDRE

O88307	1394	NRWKCDRENDCGDWSDEKDCG
O88307	1478	FGQCDRFEFECHQPKKCIPNW
O88307	1484	FEFECHQPKKCIPNWKRCDGH
O88307	1497	NWKRCDGHQDCQDGQDEANCP
O88307	1506	DCQDGQDEANCPTHSTLTCTS
O88307	1540	LSERCDGFLDCSDESDEKACS
O88307	1631	TTYQVKVQVHCLNKVHNTNDF
O88307	1861	AKAINQTAVECIWTGPKNVVY
O88307	2109	QARCLFGSQICGEPAVLLYDE
P07310	74	PGHPFIMTVGCVAGDEESYTV
P07310	254	GNMKEVFRRFCVGLQKIEEIF
P07310	283	WNEHLGYVLTCPSNLGTGLRG
P07724	77	VQEVTDFAKTCVADESAANCD
P07724	99	SLHTLFGDKLCAIPNLRENYG
P07724	125	CTKQEPERNECFLQHKDDNPS
P07724	148	PFERPEAEAMCTSFKENPTTF
P07724	192	AEQYNEILTQCCAEADKESCL
P07724	201	QCCAEADKESCLTPKLDGVKE
P07724	270	TDLTKVNKECCHGDLLECADD
P07724	277	KECCHGDLLECADDRAELAKY
P07724	289	DDRAELAKYMCENQATISSKL
P07724	302	QATISSKLQTCCDKPLLKKAH
P07724	303	ATISSKLQTCCDKPLLKKAHC
P07724	393	KCCAEANPPACYGTVLAEFQP
P07724	416	EEPKNLVKTNCDLYEKLGEYG
P07724	485	DYLSAILNRVCLLHEKTPVSE
P07724	538	AETFTFHSDICTLPEKEKQIK
P07724	583	DDFAQFLDTCCKAADKDTCFS
P07724	591	TCCKAADKDTCFSTEGPNLVT
P08752	140	RLWADHGVQACFGRSREYQLN
P08752	287	EKITQSSLTICFPEYTGANKY
P08752	326	DTKEIYTHFTCATDTKNVQFV
P14152	154	APSIPKENFSCLTRLDHNRAK
P35235	104	DVIELKYPLNCADPTSERWFH
P35235	318	NIIMPEFETKCNNSKPKKSYI
P35235	333	PKKSYIATQGCLQNTVNDFWR
P35235	463	IVDAGPVVVHCSAGIGRTGTF
P35235	490	IDIIREKGVDCDIDVPKTIQM
P45952	20	RVLRSVSHFECRTQHSKAAHK
P45952	106	GGLGLGTFDACLITEELAYGC
P45952	116	CLITEELAYGCTGVQTAIEAN
P54265	217	GHIRLADFGSCLKLQPDGMVR
P54265	319	EAQDLIRGLLCPAEIRLGRGG
P54265	608	AALAAAATLGCTGLVAYTGGL
P54265	623	AYTGGLTPVWCFPGATFAPXX
P63101	25	QAERYDDMAACMKSVTEQGAE
P63101	189	YEILNSPEKACSLAKTAFDEA
P68033	12	CDDEETTALVCDNGSGLVKAG
P68033	219	EIVRDIKEKLCYVALDFENEM
P68033	259	VITIGNERFRCPETLFQPSFI
P68033	287	HETTYNSIMKCDIDIRKDLYA
P98064	217	YPNPYPKSSECSYTIDLEEGF
P98064	247	FDIEDHPEVPCPYDYIKIKAG
P98064	334	YSFKDQVLVSCDTGYKVLKDN
P98064	372	NKIPTCKIVDCGAPAGLKHGL

P98064	402	TTYKSEIRYSCQQPYYKMLHN
P98064	437	NEVLKRSLPTCLPVCGVPKFS
P98064	441	KRSLPTCLPVCGVPKFSRKQI
P98064	636	LKKKVTKDMICAGEKEGGKDA
Q3TJ94	12	SHVRGLGLPGCLALAALVSLV
Q3TJ94	91	TDVFWAKYTVCDSVRKPRETF
Q3TJ94	236	NLAVTTLGSPCLPWNSLPAKT
Q3TJ94	333	EKTFGLGEADCGLRPLFEKKS
Q3TJ94	388	LFRKSPQELLCGASLISDRWV
Q3TJ94	479	VPFSDYIHPVCLPDKQTVTSL
Q3TJ94	533	VNLPIVERPVCKASTRIRITD
Q3TJ94	547	TRIRITDNMFCAGFKVNDTKR
Q3TRK3	68	FENQKVMYGFCSVKDSQAALP
Q3TRK3	308	ERVASASGGSCDAPAPAPFNH
Q540M5	128	DYARSKEFLMCLKGKVGPPGL
Q540M5	208	VEKEECNETLCELNISVSTLD
Q540M5	243	QVRTEKSKDVCIPPFHDDRKD
Q543S2	108	KERKTDSKMVCDVVSRMEDTE
Q64521	188	KMYDLVAGSQCLKSSYVLSKS
Q64521	309	KMDDKNVVPICQPSAGVHIVM
Q6GU14	176	TIRLKLIYDRCDLNCRIHKKS
Q6GU14	180	KLIYDRCDLNCRIHKKSRNKC
Q6GU14	313	KEVAIRIFQGCQFRSVEAVQE
Q6P8J7	288	GNMKRVFERFCRGLKEVERLI
Q6P8J7	317	WNERLGYILTCPSNLGTGLRA
Q6P8J7	397	VIDGVNYLVDCEKKLERGQDI
Q91VD9	78	RLSVAGNCRMCLVEIEKAPKV
Q91VD9	179	VKTIMTRCIQCTRCIRFASEI
Q91VD9	226	SELSGNVIDICPVGALTSKPY
Q91VD9	367	LLNKVDSDNLCTEEIFPTEGA
Q91VD9	463	QDIASGRHSFCEVLKDAKKPM
Q91VD9	554	MLFLLGADGGCITRQDLPKDC
Q99K10	117	IRNATQFAPVCPQNIIDGRLP
Q99K10	342	YARILATKVGCNVSDTVELVE
Q99K10	353	NVSDTVELVECLQKKPYKELV
Q99K10	732	DKRRHDVHRRCSPQRTTTNDL
Q99K10	778	HPHEVVLRTACPPDYTLAMRR
Q99LC5	53	AAGRLGGEVSCLVAGTKCDKV
Q99LC5	109	TQKQFSYTHICAGASAFGKNL
Q99LC5	155	VRTIYAGNALCTVKCDEKVKV
Q99LC5	159	YAGNALCTVKCDEKVKVFSVR
Q99M47	146	GKPKIFIIQACRGSQHDVPVV
Q99M47	259	PDAIGKKQVPCFASMLTKKLH
Q99M47	271	ASMLTKKLHFCPKPSKXXXXX
Q9CWS0	274	EMEKVDGLLTCCSVFINKKID
Q9CWS0	275	MEKVDGLLTCCSVFINKKIDS
Q9D8L4	153	APSVYPLAPVCGGTTGSSVTL
Q9D8L4	165	GTTGSSVTLGCLVKGYFPEPV
Q9D8L4	246	EPRVPITQNPCPPLKECPPCA
Q9D8L4	347	QDWMSGKEFKCKVNNRALPSP
Q9D8L4	393	EMTKKEFSLTCMITGFLPAEI
Q9EQN8	190	VHIDADKKKQCHILEQLKESK
Q9EQV1	65	KVGISKTTGFCTNRKNSKDPD
Q9EQV1	181	EKKTVTMIVDCKKKITKPLDR
Q9EQV1	242	YDYCDHYSPDCDLTSKAAQAQ

Q9R158	116	DLESPVIVNTCFGSLQGTLEI
Q9R158	218	YVHRNNNITTCIQDMLQIVNG
Q9R158	310	YLADVCTPYNCGVSSVLSDVM
Q9R158	344	NFGMKHDGIGCTCGLKDCLMA
Q9R158	380	EMYSVVTKRSCLYDIPEALVT
Q9R158	395	PEALVTNLTVCGNKVVEEGEQ
Q9R158	420	NSESCLQDPCCSSDCVLKPGA
Q9R158	437	KPGAQCAFGLCCKNCQFLKAG
Q9R158	450	NCQFLKAGTVCRKEKNECDLP
Q9R158	605	DGTAYAPDHICVDRKCVSKSV
Q9R158	639	GICNNKQHCHCGVTWKPPDCQ
Q9R158	682	IVYILIVLDVCIVIIIYLFSF
Q9WV42	55	LREVKAQIHSCISRHLECLRS
Q9WV42	101	QLYWLMGQFNCLIHQLEYTQN
Q9WV42	121	NKDLANQVSVCLERLGSLALK
Q9WV42	360	WLVKPDSCTNCQGNQPRGVEI
Q9WV42	378	VEIENLGNLKCLNDHLEAKKS
Q9WV42	415	HQDTWKVEEVCKANEPCTSFA
Q9WV42	429	EPCTSFAECVCDDNCEKEAMY
Q9WV42	473	KHRESLTLWLCPSRNELTEQA
Q9WV42	607	GLPAVCDLFACMQLKVDKEKW
Q9Z0L3	57	NVESVAEIFDCLGSHFTWLQA
Q9Z0L3	98	LCPRDFEDYGCACRFEMEGMP
Q9Z0L3	144	DPAKLSADVDCTNKQITCESE
Q6Z8D9	392	TTRQASFGSKCELSDDFLKKV
Q6Z8D9	454	NGAGGKDSNNCTLILTEGDSA
Q6Z8D9	721	LKPGQRKILFCSFKRNLVKEI
Q41088	214	EYIVVIGHSACGGIKGLMSFP
Q41088	241	TDFIEDWVKVCYNAKTKILAE
B9DGD1	306	PIEGDWNGAGCHTNYSTKSMR
B9DGD1	371	QFSWGVANRGCSIRVGRDTEA
O03042	172	LNKYGRPLLGCTIKPKLGLSA
O03042	247	GHYLNATAGTCEEMIKRAVFA
O48646	105	VLLIVNVASQCGLTNSNYTEL
O48646	153	GTNEEIVQFACTRFKAEYPIF
O65396	88	NGSLFDVAHMCGLSLKGKDCV
O65396	151	EHIYLVVNAGCRDKDLAHIEE
O65396	221	FQILDINGSTCFLTRTGYTGE
P10795	96	DYLIRNKWIPCVEFELEHGFV
P10795	132	WTMWKLPLFGCTDSAQVLKEV
P10795	167	IGFDNTRQVQCISFIAYKPPS
P10796	132	WTMWKLPLFGCTDSAQVLKEV
P10796	167	IGFDNTRQVQCISFIAYKPPS
P10797	132	WTMWKLPLFGCTDSAQVLKEV
P10797	167	IGFDNTRQVQCISFIAYKPPS
P19171	69	NTEPYCKQPGCQSQCTPGGTP
P19171	107	DMLKHRNDAACPARGFYTYNA
P19171	212	QLSWNYNYGLCGRAIGVDLLN
P19171	307	ADRIGFYQRYCNIFGVNPGGN
P19366	63	DTLGQEINVTCEVQQLLGNNR
P24704	101	DGTATFTITDCQIPLTGPNSI
P50883	142	EILGTCVSVGCTVDGKDPKDI
P93819	79	VVATTDAVEGCTGVNVAVMVG
P93819	155	APSIPEKNISCLTRLDHNRAL
P94072	120	EVLVVIQGTICAGFISSANKV

Q0WUV6	69	GAARLPGFHCCVGSGGEKLLP
Q2HIV2	137	KKRVGITAGLCVVIEHVPEKN
Q38946	39	MIPFREIKVECTIPKDDGTLV
Q38946	107	PYGGAKGGIGCSPRDLSLSEL
Q38946	386	NIKTMCHTHSCNLRMGAFTLG
Q56ZK3	55	PIEGDWNGAGCHTNYSTKSMR
Q56ZK3	120	QFSWGVANRGCSIRVGRDTEA
Q593N9	79	VVATTDAVEGCTGVNVAVMVG
Q6AWV3	59	LTSRATLVVHCVNKEKDLGIQ
Q9SA52	161	ALPKLEQYIYCSSAGVYLKSD
Q9SA52	176	VYLKSDILPHCEEDAVDPKSR
Q8VXW1	269	RPLKTVFISGCGELKVTNPSL
P39207	23	VQRGLIGEVICRFEKKGFTLK
Q9LR30	239	EANIREILKFCYNEKLVLLGD
Q9LR30	382	DGFNSCKNVVCNFTEGAMYSF
Q9LR30	417	QAGKVPDVFYCLKLLEATGIS
Q9FNE2	26	VVFSKTYCPYCVRVKELLQQL
Q9FNE2	80	VFIGGNHIGGCDATSNLHKDG
Q9FVT2	165	SVTLADIVTICNLNLGFATVM
Q9FVT2	303	YDPEGYSLWFCDYKYNDENMV
Q9FVT2	342	RKYSFGKMLICGSEGPFKVKG
Q9LD57	173	LGIEVTKADDCIGPEVESLVA
Q9LUT2	161	KLTEVRKNGTCPWLRPDGKTQ
Q9M7T0	89	FGLPGAYTGVCSQQHVPSYKS
Q9M7T0	114	FKAKGIDSVICVSVNDPFAIN
Q9SA56	131	NLLKLARKEQCLALGTRLRSK
Q9SF85	335	ASNVVIQRSGCTYPEKPDFNX
Q9SJU4	346	FSYARALQNTCLKTWGGKEEN
Q9XEX2	76	LKSKGVDEIICFSVNDPFVMK
Q9ZP06	130	NINAGIVKNLCTAIAKYCPHA
Q9ZP06	137	KNLCTAIAKYCPHALINMISN
Q9ZRW8	189	VPKLIAWVKKCLQRESVAKSL
Q43746	132	WTMWKLPLFGCTDSAQVLKEV
Q43746	167	IGFDNNRQVQCISFIAYKPPS
O43719	186	NQGNLKGDGLCCYLKRESVEL
O43719	187	QGNLKGDGLCCYLKRESVELA
O43719	233	EYDASKKKKKCKDYKKKLSMQ
O43719	295	NEIREDLRVECSKFGQIRKLL
O43719	462	SSPEKEAEEGCPEKESEEGCP
O43719	471	GCPEKESEEGCPKRGFEGSCS
O43719	480	GCPKRGFEGSCSQKESEEGNP
O75390	101	IRFRGFSIPECQKLLPKAKGG
O75390	211	DSMDLIAKLPCVAAKIYRNLY
O75390	359	VLRKTDPRYTCQREFALKHLP
O95886	440	SSVDQARINCCVPPRIHPRSS
P00505	106	YLPIGGLAEFCKASAELALGE
P00505	187	QGYRYYDPKTCGFDFTGAVED
P00505	272	RHFIEQGINVCLCQSYAKNMG
P00505	295	GERVGAFTMVCKDADEAKRVE
P00568	187	GSVDSVFSQVCTHLDALKXXX
P00918	205	GSLTTPPLLECVTWIVLKEPI
P04264	147	GGYGGGYGPVCPPGGIQEVTI
P06744	330	LALLGIWYINCFGCETHAMLP
P06748	21	PLRPQNYLFGCELKADKDYHF
P07288	50	VLVASRGRAVCGGVLVHPQWV

P07288	184	VDLHVISNDVCAQVHPQKVTK
P07288	198	HPQKVTKFMLCAGRWTGGKST
P09972	73	LFSADDRVKKCIGGVIFFHET
P09972	150	KDGADFAKWRCVLKISERTPS
P09972	240	KPNMVTPGHACPIKYTPEEIA
P09972	290	ASFNLNAINRCPLPRPWALTF
P11021	41	VGIDLGTTYSCVGVFKNGRVE
P11021	420	DTGDLVLLDVCPLTLGIETVG
P11177	169	SQCFAAWYGHCPGLKVVSPWN
P11177	263	AAVLSKEGVECEVINMRTIRP
P12270	75	QERLVNETRECQSLRLELEKL
P12270	224	EKGNEILELKCNLENKKEEVS
P12270	1127	TQKAESQLLECKASWEERERM
P12270	1149	KDEVSKCVCRCEDLEKQNRLL
P13861	134	DEQRCRLQEACKDILLFKNLD
P13861	330	GGNQEVEIARCHKGQYFGELA
P13861	359	ASAYAVGDVKCLVMDVQAFER
P17612	200	KRVKGRTWTLCGTPEYLAPEI
P21796	232	AAKYQIDPDACFSAKVNNSSL
P25705	244	DGSDEKKKLYCIYVAIGQKRS
P25787	137	VRPFGVSLLICGWNEGRPYLF
P28161	115	MDSRMQLAKLCYDPDFEKLKP
P28838	462	NIGKYRSAGACTAAAFLKEFV
P37837	250	NTGEIKALAGCDFLTISPKLL
P43490	39	NTSKVYSYFECREKKTENSKL
P43490	287	VSDSYDIYNACEKIWGEDLRH
P49720	122	LDPKTFKPFICSLDLIGCPMV
P50990	136	SEVIEGYEIACRKAHEILPNL
P50990	148	KAHEILPNLVCCSAKNLRDID
P50990	149	AHEILPNLVCCSAKNLRDIDE
P50990	430	AKQITSYGETCPGLEQYAIKK
P56597	203	LKNNPNKPKLCHHPIVEEPYX
P61106	40	QFTEKKFMADCPHTIGVEFGT
P62258	97	QMVETELKLICCDILDVLDKH
P62258	98	MVETELKLICCDILDVLDKHL
P81605	18	FLTALAGALVCAYDPEAASAP
Q02383	360	SSSTEERHLNCGEKGIQKGVS
Q13625	290	QNAKLQQQRECLNKRNSEVAV
Q13625	968	EGITALHNAVCAGHTEIVKFL
Q13625	1083	DDELPMKEGDCMTIIHREDED
Q15424	519	RSTNLKRDDKCDRKDDAKKGD
Q16698	17	VFFTLGSRLPCGLAPRRFFSY
Q9NVA2	150	FNYHDTRIHACLYFIAPTGHS
Q9NVA2	268	GVVQVENENHCDFVKLREMLI
Q7Z4H7	77	QSLTKEVFKFCWPPFDQKSDT
Q7Z4H7	93	QKSDTEFRKHCCEWIKRISGE
Q7Z4H7	165	FNIKPQDLHKCIARCHFARSR
Q7Z4H7	323	EVLKVMKYERCQADQARLTVD
Q7Z4H7	443	AHKQHNQENGCRGDSDTLGAL
Q7Z4H7	664	TEEKVISDCECVPQKHVLTSH
Q7Z4H7	699	KKVICKQDLECLAFTKLSETS
Q7Z4H7	743	MKILDHLEVSCNKPSTNKTML
Q7Z4H7	885	TDDTLNFLDTCDLHTEHIKPS
Q7Z4H7	926	VEQRLRTTIACSLGELPNLKE
Q8IV32	191	ALVPLKTPMPCLGAKHKAQSL

Q8IV32	238	PRKTTSKGPKCLTRKGPGAGP
Q92777	129	VDEPHADWAKCFRGKKVLGDY
Q92777	223	INSLESIYNFCDKPWVFAQLV
Q96LI6	104	KSISWDENGTCIVINEELFKK
Q99447	180	YREYADSFGKCPGGRNPWTGV
Q99447	306	LLSHFKVDLVCHGKTEIIPDR
Q9BXM0	86	KYEDALRLLQCAEPYKVSFCL
Q9BXM0	95	QCAEPYKVSFCLKRTVPTGDL
Q9H489	236	VRELRHSRTGCKFKFRFWSNP
Q9P1W8	53	VTVGKTATLHCTVTSLLPVGP
Q9P1W8	226	DPWDVRSQVICEVAHVTLQGD
Q9P1W8	271	MRVGNQVNVTCQVRKFYPQSL
Q9P1W8	329	SDQRDDVVLTCQVKHDGQLAV
Q9Y265	206	NSGAVKRQGRCDTYATEFDLE
Q9Y265	336	IVIFASNRGNCVIRGTEDITS
P09542	85	KGEMKITYGQCGDVLRALGQN
P37040	363	EESNKKHPFPCPTTYRTALTY
P37040	445	PSLRPPIDHLCELLPRLQARY
P48036	314	GDYKKALLLLCGGEDDXXXXX
P98156	67	DCADGSDEKNCVKKTCAESDF
P98156	79	KKTCAESDFVCKNGQCVPNRW
P98156	97	NRWQCDGDPDCEDGSDESPEQ
P98156	120	MRTCRINEISCGARSTQCIPV
P98156	140	VSWRCDGENDCDNGEDEENCG
P98156	161	NITCSADEFTCSSGRCVSRNF
P98156	188	DCDDGSDELDCAPPTCGAHEF
P98156	251	TSEIQCGSGECIHKKWRCDGD
P98156	264	KKWRCDGDPDCKDGSDEVNCP
P98156	273	DCKDGSDEVNCPSRTCRPDQF
P98156	318	DEVNCKNVNQCLGPGKFKCRS
P98156	360	EPLKECHINECLVNNGGCSHI
P98156	382	KDLVIGYECDCAAGFELIDRK
P98156	434	GYQMDLATGVCKAVGKEPSLI
P98156	715	WCEDDMENGGCEYLCLPAPQI
P98156	749	GYNLEENGRECQSTSTPVTYS
Q03265	244	DGTDEKKKLYCIYVAIGQKRS
Q5SS40	97	QMVETELKLICCDILDVLDKH
Q5SS40	98	MVETELKLICCDILDVLDKHL
Q61344	36	EADKKQAEDRCKQLEEEQQAL
Q61344	190	EERAEVAESKCGDLEEELKIV
Q8R081	148	LVEFEDVLGACNAVNYAADNQ
Q8R081	257	SLNGADIYSGCCTLKIEYAKP
Q8R081	258	LNGADIYSGCCTLKIEYAKPT
Q8R081	401	MNCDRVFNVFCLYGNVEKVKF
Q8R081	449	NFMFGQKMNVCVSKQPAIMPG
Q8R081	578	NGPYPYTLKLCFSTAQHASXX
Q8R4I4	105	FTPEDLNTANCGANFAFAGYP
Q8R4I4	297	LQSLKDSIYGCDSTKQLRKAE
Q8R4I4	429	DLFDTENVIVCQYDKIHRSKN
Q8R4I4	451	WKFYLKDGVMCFGGRDYVFAK
Q91X86	177	SHLVLVTRNACHLTGGLDWID
Q99KD4	157	YRGFDIGNHFCEWMYDYTYEK
Q8H0B7	152	TAYGQPEKTPCVEDSKLSALN
Q8H0B7	282	GCVAYNLGTGCGTTVLEVVKA
Q8H0B7	306	ASGKKIPIKICPRRPGDCTEV

Q8H0B7	339	WSARFGIEDMCRDQWNWAKKN

1. Xue Y, Liu Z, Gao X, Jin C, Wen L, et al. (2010) GPS-SNO: computational prediction of protein S-nitrosylation sites with a modified GPS algorithm. PLoS One 5: e11290.
